# Application of an Improved 2-Dimensional High-Throughput Soybean Root Phenotyping Platform to Identify Novel Genetic Variants Regulating Root Architecture Traits

**DOI:** 10.34133/plantphenomics.0097

**Published:** 2023-09-28

**Authors:** Rahul Chandnani, Tongfei Qin, Heng Ye, Haifei Hu, Karim Panjvani, Mutsutomo Tokizawa, Javier Mora Macias, Alma Armenta Medina, Karine Bernardino, Pierre-Luc Pradier, Pankaj Banik, Ashlyn Mooney, Jurandir V. Magalhaes, Henry T. Nguyen, Leon V. Kochian

**Affiliations:** ^1^ Global Institute for Food Security, University of Saskatchewan, Saskatoon, SK, Canada.; ^2^ NRGene Canada, 110 Research Dr Suite 101, Saskatoon, SK, Canada.; ^3^ Division of Plant Sciences and Technology, University of Missouri, Columbia, MO 65211, USA.; ^4^ School of Biological Sciences, The University of Western Australia, Crawley, WA 6009, Australia.; ^5^ Rice Research Institute, Guangdong Academy of Agricultural Sciences & Key Laboratory of Genetics and Breeding of High Quality Rice in Southern China(Co-construction by Ministry and Province), Ministry of Agriculture and Rural Affairs & Guangdong Key Laboratory of New Technology in Rice Breeding & Guangdong Rice Engineering Laboratory, Guangdong, China.; ^6^ Embrapa Maize and Sorghum, Sete Lagoas, Brazil.

## Abstract

Nutrient-efficient root system architecture (RSA) is becoming an important breeding objective for generating crop varieties with improved nutrient and water acquisition efficiency. Genetic variants shaping soybean RSA is key in improving nutrient and water acquisition. Here, we report on the use of an improved 2-dimensional high-throughput root phenotyping platform that minimizes background noise by imaging pouch-grown root systems submerged in water. We also developed a background image cleaning Python pipeline that computationally removes images of small pieces of debris and filter paper fibers, which can be erroneously quantified as root tips. This platform was used to phenotype root traits in 286 soybean lines genotyped with 5.4 million single-nucleotide polymorphisms. There was a substantially higher correlation in manually counted number of root tips with computationally quantified root tips (95% correlation), when the background was cleaned of nonroot materials compared to root images without the background corrected (79%). Improvements in our RSA phenotyping pipeline significantly reduced overestimation of the root traits influenced by the number of root tips. Genome-wide association studies conducted on the root phenotypic data and quantitative gene expression analysis of candidate genes resulted in the identification of 3 putative positive regulators of root system depth, total root length and surface area, and root system volume and surface area of thicker roots (*DOF1-like* zinc finger transcription factor, protein of unknown function, and C2H2 zinc finger protein). We also identified a putative negative regulator (gibberellin 20 oxidase 3) of the total number of lateral roots.

## Graphical abstract

**Figure:**
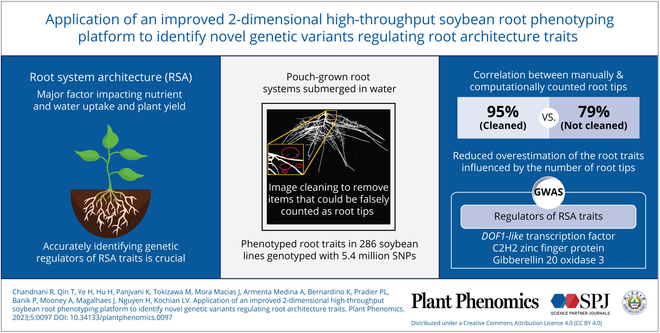

## Introduction

Root system architecture (RSA) is the spatial configuration of the root system that enables and facilitates a number of important agronomic traits, including plant soil anchorage, nutrient and water absorption, and abiotic and biotic stress signaling, and therefore is one of the major factors that can impact plant yield [1–5]. RSA variation has been reported to be shaped by evolutionary and ecological adaptations to different agro-environmental growing conditions [6] for efficient water and nutrient uptake, and to cope with abiotic stresses such as drought and mineral nutrient deficiency [7]. Different RSA ideotypes have been proposed to increase crop yield under different abiotic stresses. For example, deeper root systems in rice, which can access more deeply located water under drought, have been reported to increase grain yield under drought conditions [6], whereas shallow root systems, especially those with a larger number of longer fine lateral roots, have been reported to increase the yield of sorghum and common bean, in low P soils where much of the P (as phosphate) is fixed to clay minerals in the top soil [8–11]. The first P acquisition efficiency gene, *Pstol1* (for *phosphorus starvation tolerance 1*, was first identified in rice by map-based cloning and facilitated increases in root system size and increased rice yields on low P soils [12]. Subsequently, using candidate gene association analysis, we identified multiple *Pstol1* homologs in sorghum that were shown to condition improved grain yield in the field, in low P soils in Brazil, and this was associated with a modified RSA that had more numerous and longer lateral roots [9]. Further, root morphology traits such as total number of lateral roots and fine lateral roots were shown to increase yield of soybean and rice plants under drought conditions, supporting the notion that capturing genetic variation and understanding the genetic control of variation in RSA can be used in root trait breeding to facilitate sustainable increases in agricultural yield [13,14].

Despite its utmost importance, RSA is a complex genetic trait that can also have significant environmental responses, as roots can be plastic in their response to 3-dimensional (3D) spatial variation in the amount and availability of water and nutrients in the soil. Hence, there is much to be learned about the genetic and environmental influence on RSA, and how to use this RSA variation to enhance water and nutrient acquisition efficiency in crop plants. The phenotyping of root traits in situ in the field is limited because roots grow in an opaque and complex soil environment. The primary root phenotyping technique in the field has involved digging up root systems either by hand or by large modified harvesting equipment, washing the soil from the roots, and imaging them [15]. This is a quite useful technique, which provides the opportunity to study roots in their natural environment, but like all phenotyping techniques, it has its limitations. The major limitations for this field approach involve (a) extraction of only the top portion of the root system, (b) resulting damage and mechanical alteration to the native RSA during removal from the soil, and (c) a low throughput. Alternative higher-throughput approaches that allow for imaging of the entire root system in relatively younger plants involve growing the plants in the laboratory in transparent media, like nutrient solution or nutrient gels. A number of laboratories have developed these types of root imaging systems for imaging of root systems in 2D [16–21], and relatively a smaller number of studies have reported 3D high-throughput root phenotyping methods [22–24]. Many of the 2D high-throughput root phenotyping methods use roots growing freely in hydroponic solution to capture root growth and morphological traits [17], gel plates to capture 2D root architectural traits along with morphological traits [16,19], and pouch growth systems using layers of filter paper saturated with nutrient solution and covered with a transparent root viewing plate, or rhizotrons where plastic or glass plates enclose a thin layer of vermiculite or soil watered with nutrient solutions [18,21]. We use both 2D and 3D root phenotyping systems in our laboratory, which have complementary strengths and weaknesses. Our 3D root phenotyping platform, RootReader 3D, allows for visible imaging of roots in transparent gel or nutrient solution [25,26] and is the preferred choice for quantifying root architecture traits from 3D reconstructions of root system models based on computed tomography of a series of 2D images taken as the plant (and root system) is rotated through 360°. 2D root phenotyping has the advantage of allowing for much higher-throughput phenotyping of large mapping populations, enabling the use of genetic approaches aimed at the identification of genes underlying RSA. Some of the RSA genes identified using these approaches are *DRO-1* and *VRN*, which are responsible for deeper rooting in rice and steeper nodal root angle in wheat, respectively [7,27].

Soybean is one of the major seed and oil crop globally [28]. Although it has been explored genetically for various above-ground plant traits that impact soybean yield, only a handful of root growth studies have been conducted and majority of those are focused on root traits in response to abiotic stresses such as phosphorus deficiency and drought [14,21,29–35]. Three of the 5 previous genetic mapping studies in soybean that looked at genetic regulation of RSA traits employed biparental populations and were focused on root morphological traits [30,32,34], such as total number of lateral roots and root volume. These are clearly useful traits for improving crop root systems, but the combination of phenotyping for root growth and root architecture traits provides a much richer set of traits for possible root function improvements. Root developmental genetic networks have been characterized primarily in the model plant, *Arabidopsis thaliana* [36]. To date, only a few major genes influencing RSA with a role in water and nutrient acquisition have been identified in crops species. Examples of such genes are *DRO-1* [7], *VRN-1* [27], and *Pstol1*, with Pstol1 improving P acquisition efficiency via enhanced root surface area in rice [12] and sorghum [9].

Despite the abundance of 2D root phenotyping studies in the literature, we show that improvements in our phenotyping and image preprocessing pipeline can significantly increase the correlation between manual root trait measurements and computationally based root image analysis measurements of root traits. In this study, we employed a large and genetically diverse soybean association panel genotyped with ~5 million single-nucleotide polymorphisms (SNPs) to conduct a detailed analysis of both root growth/morphology and RSA traits. This generated genome-wide association study (GWAS) peaks with high resolution, and based on the location of the SNPs that were in strongest associations with RSA traits, we identified and characterized several candidate genes possibly involved with either improved acquisition of water and/or major nutrients (N and P). These include candidate genes that might condition deeper root systems or those root systems with more lateral roots resulting in greater total root surface area and volume. For some of these candidate genes, an increase or decrease in root trait values along with similar or opposite trends in the gene expression of a specific candidate genes suggested that some of these candidate genes can be positive or negative regulators of specific root traits, respectively.

## Materials and Methods

### Plant material and soybean root growth in 2D imaging pouches

**Fig. 1.**
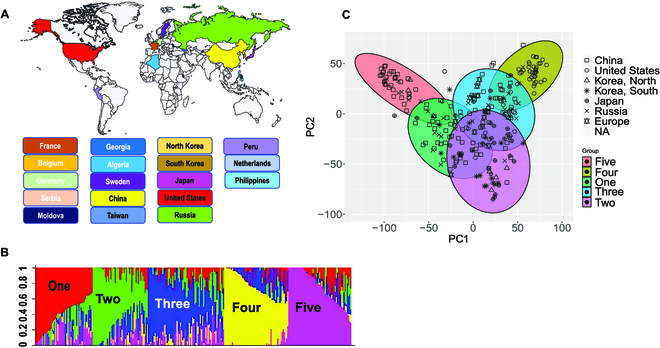
Geographical origin and population structure of soybean association panel. (A) Geographical countries of origin for soybean accessions phenotyped for 2D RSA and root morphology traits. (B) Five subpopulations were identified based on structure analysis of the 286 soybean lines. Structure analysis was conducted by using ~12,000 genome-wide SNPs using the STRUTURE software with 10 iterations of 12,000 burning periods. (C) Principal components analysis was performed with 367,303 randomly selected genome-wide SNPs in R by using the prcomp function. The PCA analysis results are consistent with the existence of 5 subpopulations.

A total of 286 soybean lines from the U.S. Department of Agriculture (USDA) germ plasm collection were obtained from the Soybean Genetics and Genomics Lab, University of Missouri, USA. These lines were selected based on variation in geographic origin (more than 12 countries; Fig. 1), maturity group (described in Table S1), and genotypic diversity, and were employed in this study for evaluation of root system growth and morphological and architectural traits.

**Fig. 2.**
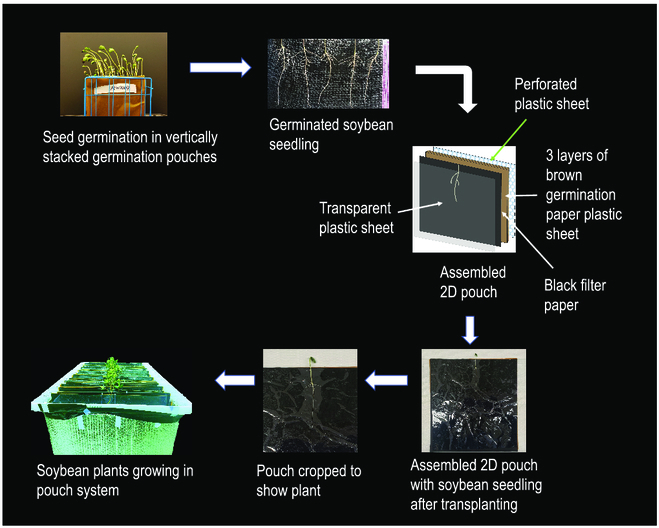
Workflow for growing plants in 2D pouch system. Soybean seeds are germinated in vertically stacked germination paper sitting in water. After germination, 5 uniformly growing seedlings are selected and transplanted into the 2D pouches. The pouch growth system is a 16 × 16 inch sandwich with an outermost layer of moisture-resistant, thin, flexible, and clear polyester film followed by a layer of black filter paper for enhanced contrast between the roots and the background, 3 layers of brown germination paper saturated with nutrient solution, and a bottom layer consisting of a 0.125″ thick perforated polypropylene plastic sheet to provide rigidity to the pouch with perforations, enabling better nutrient and O_2_ provision to the roots. Subsequently, the 2D pouches are placed in hanging folders suspended in a hydroponic tank filled with 125 L of aerated nutrient solution for growth.

A total of 25 seeds per line were germinated in vertically stacked layers of water-moistened germination paper to minimize alterations in root architecture during transplanting to the 2D growth pouches (Fig. 2). Five uniform soybean seedlings were selected for each genotype and transplanted carefully into the 2D pouch growth system 5 days after germination (Fig. 2). The pouch growth system is 16 × 16 inches in size with an outermost layer of moisture-resistant, thin, flexible, and clear polyester film (https://www.mcmaster.com/8567K14/) and under that is a layer of black filter paper (grade 8613, Ahlstrom, distributed by VWR) for enhanced contrast between the roots and the background, followed by 3 layers of brown germination paper and a bottom layer of 0.125″ thick polypropylene plastic perforated sheet (https://www.mcmaster.com/9293T73/). This sheet provides physical structure to the pouch, and we found the perforations greatly enhanced plant growth due to better provision of nutrients and oxygen to the roots from the aerated nutrient solution in which the pouches were immersed. Prior to transplanting the seedling into the pouch, the black filter paper and brown germination paper layers were soaked with hydroponic nutrient solution and placed on the plastic perforated sheet. The nutrient solution used is a modified Magnavaca solution [37] and consisted of 3.53 mM Ca(NO_3_)_2_, 1.3 mM NH_4_NO_3_, 0.59 mM KCl, 0.58 mM K_2_SO_4_, 0.57 mM KNO_3_, 0.86 mM Mg(NO_3_)_2_, 0.2 mM KH_2_PO_4_, 20 μM FeEDDHA, 9.1 μM MnCl_2_, 25 μM H_3_BO_3_, 2.3 μM ZnSO_4_, 0.63 μM CuSO_4_, and 0.83 μM Na_2_MoO_4_. In the next step, each soybean seedling is placed in the middle top of the sheet of black filter paper and then covered with a clear and pliable polyester sheet, which holds the root system in place and prevents drying. Finally, the whole system consisting of the perforated polypropylene sheet as the base, then 3 sheets of germination and 1 sheet of black filter paper, with the seedling covered by the polyester sheet, is held together using 6 plastic shirt clips (Amazon.ca), with 2 clips on the top horizontal side of the pouch, 1 on each side of the seedling, and 2 clips on bottom horizontal sides of the pouch system. One hundred pouches were assembled and placed in a 125-liter (18 × 18 × 24 inch) polyvinyl chloride tank filled with aerated hydroponic solution (Fig. 2). Hydroponic solution pH was adjusted every other day to keep it between 5.6 and 5.8. Nutrient solution was resupplied every 3 days. Soybean seedlings were grown for 8 days after transplanting (DAT) in a growth chamber with growth conditions of 16-hour light period (27 °C), 8-hour dark period (22 °C).

### Acquisition of digital root phenotyping data

**Fig. 3.**
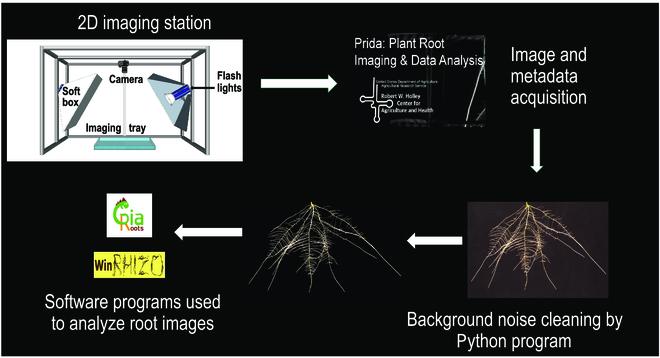
Image acquisition and analysis workflow. Root growth pouches are opened, and then the plastic film is carefully removed from root system. Subsequently, the pouches are placed inside an imaging tray filled with water. Thereafter, a transparent glass plate is placed on the top of the root system. Plant Root Imaging and Data Analysis (PRIDA)—a Python-based image acquisition and data management software—was used for collecting and storing raw images and project meta-data into a single Hierarchical Data Format (HDF5) file. Root images are cropped to the region of interest and passed through a high-throughput image preprocessing pipeline to segment RSA from the background, which improves root image to noise ratio and trait computations. Cleaned images are analyzed to extract root morphological and architectural traits using a combination of WinRhizo, GiaRoots, and other custom software scripts.

**Fig. 4.**
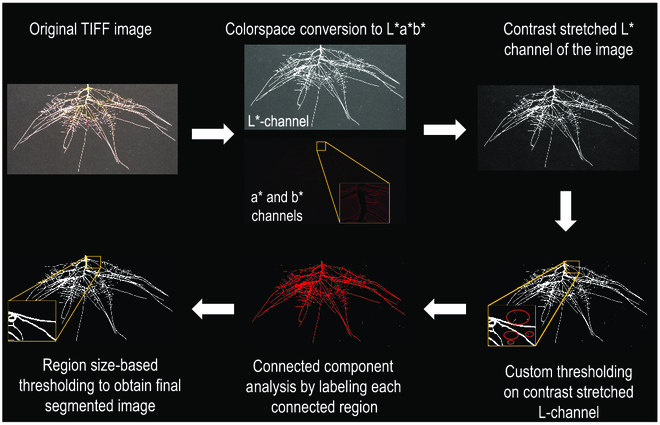
Image segmentation and cleaning pipeline workflow. In the first step, images are converted into LAB color space. In the second step, contrast of the L channel of the image is stretched and then a customized global threshold is applied on the L channel. After thresholding in LAB color space, we performed connected component analysis on the mask image and labeled each of the connected regions with a pseudo-color. Each of these regions is then analyzed to check their projected area in number of pixels. After this processing is completed, the final mask is multiplied with the original image to generate a “cleaned” image.

This digital phenotyping system has the throughput to image the roots of 100 plants a day by a single person. To capture the images, first, the pouches are removed from the hydroponic tank and the clear polyester film is removed, taking care not to move the seedling. Then, the pouch is carefully placed in the imaging tray that is filled with water (Fig. 3). Imaging the root systems submerged in water is advantageous because it removes any reflection from the black filter paper and from water menisci that form between the edge of the root and the filter paper, when the roots are imaged in air. These reflections can result in overestimation of root traits, as the menisci can be quantified as part of the root system. Images are captured using a Nikon D7200 DSLR camera with a 50-mm lens. The imaging camera is attached to a vertical rod and a gear-driven manipulator, which allows for adjustment of the height of the camera according to the size of the root system (Fig. 3). The camera is controlled by our custom-written Python-based software, Plant Root Imaging and Data Analysis (PRIDA), for collecting and storing raw images and meta-data into a single Hierarchical Data Format (HDF5) file. Images are captured and stored in HDF5 file format, and then TIF files are extracted from the HDF5 files. Root system images were captured at 5 and 8 DAT (10- and 13-day-old seedlings) (Fig. S1). TIF images were cropped to only include regions of interest and passed through a high-throughput image preprocessing pipeline to segment RSA from the background, which improves root image to noise ratio and trait computation. We have developed an image segmentation pipeline to extract RSA from the images by rejecting any background objects that are not root (Fig. 4). This preprocessing workflow is implemented In Python programming language and utilizes computer vision software packages such as OpenCV (https://opencv.org/) and scikit-image (https://scikit-image.org/). Our workflow applies a global threshold to the root system images in LAB color space, specifically after analyzing the lightness channel. After thresholding in LAB color-space, we perform connected component analysis on the mask image and label each of the connected regions with a pseudo-color. Each of these regions is then analyzed to check their projected area as the number of pixels. This analysis establishes whether a particular region is part of the root system or not, using a specific shape/size threshold. This process helps in rejecting all the nonroot particles in the image that look like roots but are smaller in size and not connected to the root system. We also calculated the distance between the center of detected objects and the center of the image to identify if the detected object is too far away from the image center. This was done to reject any other objects placed in the image plane such as a ruler. After this processing is completed, the final mask is then multiplied with the original image to generate a “cleaned” image. Cleaned images are analyzed to extract root morphological and root system architectural traits using a combination of WinRhizo (Regent Instrument Inc., Ville de Québec, QC Canada), GiaRoots [38], and custom software scripts we have developed.

### Conversion of digital data into quantitative root trait data

The image preprocessing, segmentation, and cleaning pipelines can be run in batch mode on thousands of images to generate cleaned images, and these images are then analyzed to get the root traits on batch of thousands of images with WinRhizo software in a day. Seven root morphological traits—total root length (TRL), total root volume (TRV), average root diameter (DIM), total number of root tips (TRT), total surface area (TSA), total surface area of thinner roots (diameter class 0 to 0.5 mm diameter [TSA1]), and total surface area of thicker roots (diameter class 1 to 1.5 mm diameter [TSA3])—were obtained from WinRhizo software. Five root system architectural traits—total root system width (TRW), total convex area (TCA), root depth index (RDI), solidity (SOL), and center of projected mass (COPM)—were calculated from root images at 5 and 8 DAT (Table 1). Two of the 5 architectural traits, total root width (TRW) and total convex area (TCA), were obtained using GiaRoots, whereas root depth index (RDI) and solidity (SOL) were calculated from multiple traits determined using WinRhizo and GiaRoots. Center of projected mass (COPM), the vertical distance of the root system center of mass from the root/shoot junction, was calculated using custom-made program in Python. To evaluate root system depth, it is clear that tap root depth/length is not an accurate measure as it is usually <20% of the total root system length. Therefore, to estimate this trait, we derived a root depth index (RDI), which is the ratio of root surface area in the lower two-thirds of the root system divided by the total root system surface area obtained from WinRhizo. Using this index, we estimate the proportion of the total root system surface area in the lower two-thirds of the root system that includes the lateral roots emerging from the tap root. A second trait that we use to estimate root system depth is center of mass (COPM), which is calculated as the vertical distance (or Y coordinate of the center of projected mass or area covered by pixels) from the root-shoot junction. COPM represents distribution of root biomass (cumulative root pixel area) along the *Y* axis of the root system. Higher values of COPM represent a higher proportion of biomass in deeper layers of soil. We had previously found that COPM is a good predictor of root system depth [25]. Solidity (SOL) was calculated as the ratio of area of root pixels divided by the total root convex area (TCA). Higher SOL values indicate that a larger proportion of the convex area (soil area encompassed by roots) is occupied by roots, indicating the roots are more closely spaced than a root system with a smaller solidity value.

**Table 1.** Descriptive statistics of root morphological and architectural traits phenotyped.

Traits	Mean	Maximum	Minimum	SD	SEM	CV	H^2^
TRL (cm)_5DAT	138.77	340.89	39.13	51.63	3.05	37.21	0.89
TSA (cm2)_5DAT	32.00	89.47	8.17	11.73	0.69	36.67	0.88
DIM (mm)_5DAT	0.74	1.13	0.56	0.08	0.00	10.99	0.83
TRV (cm3)_5DAT	0.60	1.91	0.14	0.24	0.01	40.77	0.84
TRT_5DAT	85.24	213	26.5	28.89	1.71	33.89	0.84
TSA1_5DAT	3.80	13.97	0.34	2.56	0.15	67.42	0.89
TSA3_5DAT	4.41	13.1	0.6	2.08	0.12	47.17	0.83
TRW (cm)_5DAT	10.63	20.57	3.64	2.92	0.17	27.50	0.82
TCA (cm2)_5DAT	99.55	239.38	30.86	36.32	2.14	36.48	0.86
RDI_5DAT	0.44	0.74	0.22	0.11	0.01	23.94	0.74
SOL_5DAT	0.11	0.17	0.06	0.02	0.00	19.27	0.76
COPM_5DAT	4.75	7.67	2.84	0.88	0.05	18.58	0.85
TRL (cm)_8DAT	248.38	739.91	71.41	105.93	6.25	42.65	0.92
TSA (cm2)_8DAT	56.61	162.18	14.23	21.70	1.28	38.34	0.9
DIM (mm)_8DAT	0.74	0.99	0.51	0.08	0.00	10.80	0.83
TRV (cm3)_8DAT	1.05	2.85	0.23	0.39	0.02	37.70	0.86
TRT_8DAT	153.78	377.5	50.33	57.79	3.41	37.58	0.89
TSA1_8DAT	10.98	46.1	2.4	7.28	0.43	66.30	0.92
TSA3_8DAT	7.56	22.93	1.19	3.38	0.20	44.77	0.83
TRW (cm)_8DAT	14.41	28.2	6.58	4.01	0.24	27.81	0.85
TCA (cm2)_8DAT	160.74	462.5	40.68	69.57	4.11	43.28	0.92
RDI_8DAT	0.51	0.8	0.23	0.11	0.01	21.49	0.74
SOL_8DAT	0.12	0.19	0.06	0.03	0.00	21.74	0.75
COPM_8DAT	5.79	10.83	3.56	1.26	0.07	21.70	0.9

Note (abbreviations for traits): Total root length (TRL), total root volume (TRV), average root diameter (DIM), total surface area (TSA), total surface area of thinner roots [or diameter class 0 to 0.5 mm diameter (TSA1)] and surface area of thicker roots [or diameter class 1 to 1.5 mm diameter (TSA3)], total root width (TRW), total convex area (TCA), root depth index (RDI), solidity (SOL), and center of projected mass (COPM) were calculated from root images at 5 and 8 DAT.

The scale for image analysis was obtained by placing a ruler in the very first image of the imaging session, and then the camera height was kept constant for that imaging session to maintain the same scale for all root systems.

### Genotyping and population structure analysis

Whole-genome resequencing (15× to 20× coverage) was performed for 286 soybean lines, and SNPs were extracted using the soybean cultivar, William82, as a reference line [39,40]. More than 10 million SNPs were filtered to remove minor allele frequencies of 0.05 or lower, and missing data were less than 10%. A total of 5.4 million SNP markers were used for GWAS. Marker density along each chromosome was plotted with the MVP package in R [41]. Population structure was analyzed using the STRUCTURE software package [42] by selecting 12,000 evenly spaced genome-wide SNPs. A burning period of 12,000 was applied with 10 iterations. Principal components analysis (PCA) was carried out in R using the prcomp function. For PCA analysis, a subset of 367,303 randomly selected SNPs was used.

### Statistical data analysis and GWAS

Descriptive statistics, correlation, and PCA analysis were performed using R statistical analysis software. Mixed model analysis and calculation of variance components were performed using the lme4 package. Broad sense heritability was calculated using the formula Vg/(Vg + (Ve/*r*)), where Vg is the genetic variance, Ve is the residual variance, and *r* is the number of replicates [5]. GWAS was conducted in R using the MVP package [41]. Three types of GWAS models (General Linear Model, Mixed Linear Model, and FarmCPU) were run, and in the MLM model, most of the observed *P* values of associations were below cumulative *P* values in the qq-plot, indicating overcorrection of type I error. Therefore, the FarmCPU model was chosen to identify significant associations. The qq-plots for all 3 models for all traits are presented in Figs. S2 and S3. The Bonferroni correction [43] was applied for multiple test correction by treating each linkage block as one testing marker. The number of linkage blocks was calculated with Plink software by scanning SNPs within 200-kb sliding windows. SNP loci with *R*^2^ value of 0.2 or higher defined a single linkage block. The *P*-value threshold was calculated as −log (0.05/212612) = 6.6, where 212,612 is the number of linkage blocks calculated as described above. Similar to identifying QTLs (quantitative trait loci), a single significantly associated SNP with the highest *P* value was selected from all SNPs significantly associated with a given trait within a linkage block.

### Candidate gene identification and expression analysis

Genome-wide linkage disequilibrium (LD) decay was calculated with PopLDdecay software [44], which involves pairwise correlations among SNPs plotted against distance throughout the genome. Although average LD decay in our population is ~150 kb, we only looked for candidate genes in very tightly linked regions (10 kb) on both sides of significantly associated SNPs. This was followed by exploration of functional annotation information for those genes to shortlist the candidate genes underlying root trait QTLs. Before selecting the most promising gene for relative expression analysis, LD for specific regions surrounding candidate genes and significantly associated SNPs were calculated and visualized with the Haploview software [45]. Then, we conducted Fisher’s exact test for statistical significance of LD between the significantly associated SNP and the genic SNP [46]. The most promising candidate genes were selected for expression analysis only if the whole region from the most significant SNP to the genic region was in LD.

### RNA extraction and real-time quantitative PCR

Three replicates of 6 to 8 genotypes [3 to 4 soybean lines each with contrasting alleles for SNP markers (associated with the traits)] were grown in the pouch system, and root tissues from 3 biological replicates were collected for each genotype to extract RNA. RNA was extracted using the TRIzol (Invitrogen, catalog #15596026) method from root tissue of plants harvested at 8 and 11 days after germination. Five micrograms of total RNA was reverse-transcribed using the Maxima First Strand cDNA Synthesis Kit (Thermo Fisher Science, USA). Synthesized cDNA was diluted 5-fold with nucleotide-free water, and 1 μl of the cDNA solution was used for quantitative real-time polymerase chain reaction (qRT-PCR) analysis with gene-specific primers. These primers for qRT-PCR expression analysis of candidate genes were designed using Primer3 software [47]. Amplification curves for each primer showed only a single peak confirming only target gene amplification. RT-PCR quantification was performed using an Applied Biosystems QuantStudio 6 real-time thermocycler with PowerUp SYBR Green Master Mix (Applied Biosystems, A25776). The ACT11 and ELF1B genes were included as reference genes in all reactions as control for RNA quantity. Relative expression levels were determined using the ΔCt method [48] of relative quantification.

For evaluation of the promotor region of candidate genes for existence of structural variants, 2-kb DNA sequences upstream of genic sequences of the lines selected based on haplotypes were extracted from Bam files after blasting the whole-genome resequencing data to the reference genome for William 82 V4, using Integrative Genomics Viewer [49]. The extracted sequences were aligned with MEGA X to identify sequence variation among the selected lines [50].

## Results

### Improvements in the 2D root growth pouch system, root imaging, and image processing, which enhance root growth, image quality and resolution, and accuracy of root trait computation

Until now, most of the studies have employed the method of rolling the seeds in a roll of wet germination paper to germinate seeds. However, in our experience, this method of germination often alters the root initiation angles of the first lateral roots that can change the RSA as the plant develops. Therefore, to maintain root initiation angle, we modified the germination approach in which seeds were germinated in vertically stacked layers of water-moistened germination paper to minimize alterations in root architecture during transplanting to the 2D growth pouches (Fig. 2). Furthermore, most of the previously published 2D pouch root phenotyping studies have been conducted using blue germination paper, which provides moderate contrast between the white roots and blue background, but the moderate contrast can be a limiting factor when thinner and finer lateral roots are being imaged. This increases the difficulty for the root segmentation program to distinguish between roots and the background. In our phenotyping pipeline, we were able, after much searching, to obtain black filter paper, which significantly enhanced the contrast between the roots and the black background. The high contrast achieved between relatively bright root systems and the background facilitated more accurate detection and extraction of root structures, enhancing the precision of segmentation algorithms.

**Fig. 5.**
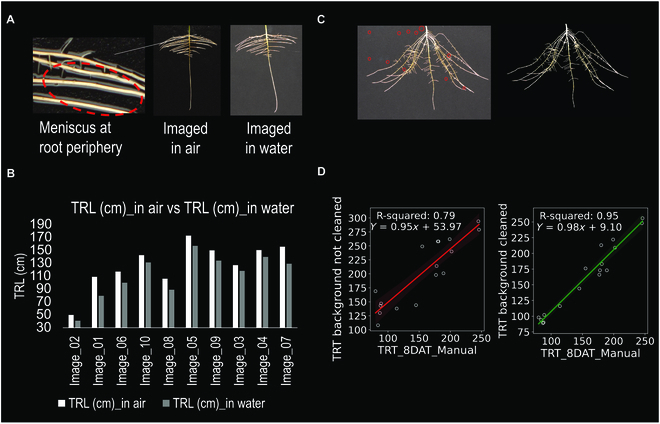
(A) Images of the same root system imaged in air versus submerged in water, side by side, and the zoomed region shows the meniscus around the roots imaged in air. (B) Overestimation of root length for all the root systems imaged in air, compared to those imaged submerged in water. (C) Root systems imaged in water where the background of the image on the left has not been computationally cleaned, and the particles mistakenly read as root tips by the segmentation program are circled in red. The right root image is the same image with the background cleaned of background noise due to root debris, dust, and other nonroot materials. (D) Regression plots and correlation between manual root tip counting and uncleaned (*R*^2^ = 0.79) and cleaned images (*R*^2^ = 0.95).

Also, similar to previous pouch studies, our imaging of the roots was initially performed in air on the photo bench after removing the pouch from the hydroponic solution. However, when using this approach, we encountered the issue of water menisci forming at the root/filter paper junction, which was measured as an “extended” part of the root system due to the reflection of light from the water meniscus (Fig. 5A). Hence, we conducted an experiment where the roots of 10 plants of the same cultivar were imaged in air on the photo bench and another 10 plants of the same cultivar were submerged in a shallow tray of water on the photo bench. In both cases, root images were processed and root traits were quantified using WinRhizo software. Paired *t* test analysis of the results showed significant differences in the mean TRL, TSA, TRV, and TRT values (Table S2) between the 2 types of root imaging. These observations supported the hypothesis that root images captured in air will lead to overestimation of root traits and forced us to fine-tune and use the underwater root imaging approach (Fig. 5B).

When imaging the roots on the black filter paper in pouches inside water to remove image errors due to the root/filter paper meniscus, we noticed that small white particles (combination of root debris and dust) were also being included as pieces of roots and not background, which resulted in each of them being counted as a root tip (Fig. 5C). Therefore, before the analysis of the water submerged root images, to validate the number of root tips (and thus the number of roots), we conducted an experiment to count the root tips manually and compared it with the number of root tips automatically counted for the water submerged root images. The results of this experiment agreed with our hypothesis that these small particles are often counted as false root tips (Table S3). Hence, we applied the image segmentation and cleaning pipeline (Fig. 4), which removed the background noise caused by the small particles. When we compared the manually counted number of root tips of a subset of 16 uncleaned and cleaned submerged in water images, we observed that correlations (*R*^2^) between manually counted root tips and computationally derived root tip number improved from 0.79 (without the background correction) to 0.95, when the background cleaning correction was employed (Fig. 5D).

We also used statistical genetics to estimate the reproducibility of our root trait measurements by calculating the broad-sense heritability of root morphological and architectural traits for multiple replicates of plants. This resulted in moderately high (0.74) to high (0.89) broad-sense heritability for root traits from plants grown for 5 DAT and also for 8 DAT (0.74 to 0.92) (Table 1). Moderate to high heritability is a plant breeding-based measure of reproducibility of our root phenotyping techniques.

Other pouch systems have a solid plastic background to provide the pouch rigidity and a base to place the sheets of germination paper and then the seedling on top of the final sheet of blue filter paper. When we first built our pouches with a solid backing, we noticed the roots grew less vigorously than when grown freely in hydroponics. Hence, we replaced the solid plastic backing with a plastic perforated sheet and vigorously aerated the nutrient solution in which the pouch was immersed in. We found that this significantly increased plant growth and especially root growth compared to plants grown in a pouch with a solid plastic backing (data not shown).

### Population structure aligns with country of origin

Population structure analysis generated a Delta K plot that had 3 peaks based on the top 3 Delta K values, representing 2, 3, and 5 possible subpopulations. Although the top Delta K value was for 2 population subgroups, the second highest Delta K value suggested 3 subpopulations and the smallest Delta K of top 3 values suggested that soybean has 5 subpopulation groups (Fig. S4). However, when grouping into 2 and 3 subpopulations, soybean accessions originating from Russia and European countries (Germany, France, Moldova, and Belgium) were mixed with accessions from China, United States, and eastern Asian countries (Japan and Koreas). While closely examining the grouping as 5 subpopulations, we were able to separate the accessions of European origin into one group. PCA also supported clustering accessions according to country of origin rather than grouping based on maturity groups (Fig. 1). Chinese and Russian accessions were distributed among groups 1, 3, and 5. East Asian accessions (Japan, South Korea, and North Korea) were mainly grouped in subpopulation 2. U.S. accessions were mainly (>95%) grouped within subpopulation 4. Further, more than 90% of the European accessions were grouped only in subpopulation 3.

### Variation among root traits and their distribution

We observed a large amount of phenotypic variation in the panel, a subset of which is shown qualitatively in Fig. S5 using 12 different soybean genotypes. Variation in root system size, number of lateral roots, and distribution of lateral roots along the tap root are readily apparent, and this is supported by the quantitative data for the 7 root morphology and 5 root architecture traits (Table S4). TRL_8DAT was significantly positively correlated with TSA, TRT, TRV, TCA, COPM at 8DAT, and TRW (*R*^2^ > 0.5) and negatively correlated with DIM (Fig. S6 and Table S5). Some of these correlations would be expected as TRL is mathematically associated with the calculation of TSA, TRT, TRV, and TRW, and it would not be surprising to see correlations between TRL and TRT. TCA_8DAT and TRW_8DAT were the architectural root traits most significantly positively correlated with TRL_8DAT and not RDI or SOL (Fig. S6 and Table S5). This suggests that variation in TRL might be mostly due to differences in TCA and TRW among the genotypes, or vice versa. There also was a significant positive correlation between DIM and SOL. Additionally, COPM was positively correlated with all the traits except SOL and DIM_8DAT (Fig. S6 and Table S5). The first 3 principal components from PCA analysis explained a total of 71.6% of the variation among root traits. PC1 accounted for 45.6% of the variation, and PC2 accounted for 17.4% of the variation (Fig. S6). Additionally, PC3 explained 8.6% of the variation (data not shown).

### Variation in root morphological and architectural traits between subpopulations and maturity groups

Apart from the significant overall genetic variation for root morphological and architectural traits, variation in RSA traits was examined among the members of the early (MG 0, MG I, and MG II) and late (MG III, MG IV, MG V, and MG VI) maturity groups at the final growth stage, 8DAT (Figs. S7 and S8). The root morphological traits, TRL, TRT, and TSA of fine roots (TSA1), have larger values in the early maturity soybean accessions compared to the late maturity lines. However, DIM exhibited an opposite trend, and roots were on average thinner (*P* < 0.01) in early maturing soybean varieties (Fig. S7). Among root system architectural traits, COPM and SOL had contrasting trends between early and late maturity groups (Fig. S8). SOL was higher (roots growing closer to each other) in late maturity lines, whereas the early maturity groups had significantly (*P* < 0.01) higher values (deeper root systems) for COPM (Fig. S8). Furthermore, members of the early maturity groups had higher TRW and significantly higher (*P* > 0.01) TCA compared to those in late maturity groups (Fig. S8). These results agreed with previous reports of finer deeper roots in early maturity mung bean lines [51].

### GWAS results, identification of novel loci associations, and hotspots related to root traits

GWAS identified a total of 113 putative loci for all root morphological and architectural traits, except COPM_5DAT, TCA_5DAT, SOL_8DAT, and RDI (Table S6 and Figs. S9 and S10). A total of 15 of these 113 loci are putative pleiotropic loci (PPQ) related to 2 or more root traits (Table S7). The highest number of loci [25] was identified on chromosome 14, and all of them were related to TRT_5DAT, whereas chromosome 3 had only a single locus that was associated with TSA1_8DAT. A total of 8 chromosomes contained putative pleiotropic loci. Chromosome 2 contained the highest [5] number of pleiotropic loci associated with the traits: TRL_8DAT, TSA_8DAT, TSA1_8DAT, TSA3_8DAT, and TRV. A number of significant GWAS peaks were observed in the Manhattan plot for root morphological traits (Fig. S9) and root system architectural traits (Fig. S10). With regard to candidate gene analysis, we identified 5 significant GWAS peaks for root architectural traits SOL_8DAT, TCA_8DAT, and COPM_8DAT (Fig. S11) and 4 significant GWAS peaks for morphological traits TRL_8DAT, TRV_5DAT, TRT_5DAT, and TSA3_5DAT (Fig. S12) that were in close vicinity to putative candidate genes with possible functions in root development, based on their annotation. Candidate genes were examined in more detail below.

We defined a genomic region as a root trait “hotspot” if it was less than 750 kb in size and contained 5 or more putative loci related to one or more root traits. We identified a total of 5 hotspots that contained 5 to 26 loci (Table S8). Chromosomes 14 and 20 harbored major hotspots for TRT and COPM traits, respectively. To differentiate novel identified QTLs (GWAS peaks), we investigated colocalization of our significant associations with previously reported QTL regions related to root morphological and architectural traits. To investigate colocalization of our GWAS peaks with previously identified root trait QTLs in biparental mapping studies, we considered colocalization to occur if the physical position of a specific GWAS peak was within the QTL interval. In previous publications comparing root GWAS peaks and QTL, peaks were considered colocalized if located within 350 kb of reported GWAS peaks. Although average LD decay throughout the genome have been reported to be up to 292 kb per chromosome [40], to account for centromeric regions, we have selected 350 kb of physical map distance between the SNPs as the maximum distance for colocalization. A total of 66 (58%) of our 113 significant GWAS associations colocalized with published root trait QTL regions previously mapped in the soybean genome (Table S9) [14,29,30,32,33,35,52–54]. For our GWAS peaks for TRW_5DAT, TRW_8DAT and TSA_8DAT, TSA1_5DAT, TSA1_8DAT, and TSA3_8DAT, we could not find QTL previously reported for those regions. Since most previous studies concentrated on root morphological traits rather than architectural traits due to the type of phenotyping used, it was intriguing to see which of the architectural trait loci from our study aligned to morphological trait QTLs from other studies. Interestingly, putative GWAS loci for COPM colocalized with root volume, weight, and lateral root density QTLs (Table S9). Also, GWAS peaks for SOL colocalized with root weight, root bushiness, and root length QTLs from previous studies mapped to the same region as our GWAS peaks for TCA (Table S9). Further, TRV GWAS peaks mapped to the physical region aligned with QTL intervals for root length, weight, lateral root density, bushiness, and root width. Finally, putative GWAS loci for TRT colocalized with previously identified root weight QTL.

### Most promising candidate genes within or in close vicinity to GWAS peaks

Selection of candidate genes within or close to GWAS peaks was based on their putative functional relationship with root morphological traits based on gene annotation in other crops or Arabidopsis. We identified 21 candidate genes, and 19 of them were within 10 kb of the most significant SNP associations (Table 2). Five out of the 21 genes were associated with genic SNPs and 10 were found to be within 5 kb next to significant SNPs (Table 2). Candidate genes were related to many families including the gibberellic family, C2H2 zinc finger proteins, serine/threonine protein phosphatases, mitogen-activated protein kinases, transferases, peptidyl-prolyl cis-trans isomerases, DNA polymerases, ZF-HD homeobox like proteins, F-box-like proteins, and members of the WRKY transcription factor family.

**Table 2.** Putative candidate genes in the close vicinity of significantly associated GWAS peaks. Regarding names for QTLs, PPQ stands for potentially pleiotropic QTL, which are the GWAS peaks listed in Table S7 that are significantly associated with 2 or more root traits.

Name of QTL	Locus	Chr	Pos	Distance from putative candidate gene	Putative candidate gene
QDIM_8DAT_03	1_49305802	1	49305802	8,238	Serine/threonine phosphatases (PP2C)
QTRL_8DAT_01	2_8678721	2	8678721	Genic	WRKY transcription factor-like protein (Lazy 1)
PPQ02(TRL_8DAT, TSA_8DAT)	2_9448766	2	9448766	1,061	Eukaryotic aspartyl protease
PPQ04(TRL_8DAT, TSA1_8DAT)	2_8593061	2	8593061	4,307	Uncharacterized protein
PPQ06(TSA_8DAT, TRV_8DAT)	2_9883206	2	9883206	6,277	UDP-glucosyltransferase71c5
QTCA_8DAT_04	4_47068983	4	47068983	3,199	DNA-directed DNA polymerase
QTRL_8DAT_04	4_47283354	4	47283354	Genic	Mitogen-activated protein kinase kinase kinase (MAP3KA)
QCOPM_8DAT_01	4_48983360	4	48983360	2,554	DOF AFFECTING GERMINATION 1
QDIM_8DAT_05	5_34851234	5	34851234	8,287	Serine/threonine protein phosphatase 2A (PP2A-3)
QTCA_8DAT_09	6_6108102	6	6108102	3,832	CALMODULIN LIKE 42
QTRV_5DAT_04	7_4869847	7	4869847	Genic	1-Phosphatidylinositol 4-kinase and ubiquitin-like
QTRT_8DAT_01	9_37109514	9	37109514	1,317	F-box protein Phloem Protein 2-A13
QTRV_8DAT_02	11_7681279	11	7681279	Genic	Peptidyl-prolyl cis-trans isomerases; P-loop containing nucleoside triphosphate hydrolase
QTRL_8DAT_05	11_17101313	11	17101313	2,387	TCP-1/cpn60 chaperonin family protein
PPQ12(TSA3_8DAT, TRV_8DAT)	12_33823378	12	33823378	3,989	ZF-HD homeobox like protein
QTRT_5DAT_28	14_30641451	14	30641451	9,781	Gibberellin 20 oxidase 3-like
QDIM_8DAT_08	19_42651593	19	42651593	319	F-box like 17 protein containing LRR
QSOL_5DAT_02	20_2376802	20	2376802	8,485	O-FUCOSYL TRANSFERASE 1
QCOPM_8DAT_12	20_31095132	20	31095132	7,620	SUGAR WILL EVENTUALLY BE EXPORTED TRANSPORTER (SWEET)
PPQ11(TSA3_8DAT, TRV_5DAT, TSA3_5DAT)	20_42450695	20	42450695	4,424	EDF1
PPQ08(TSA3_5DAT, TRV_5DAT)	20_42464855	20	42464855	Genic	C2H2 zinc finger (DOT-5)

Based on literature review of genes that were in LD with our GWAS peaks, we identified genes or gene families that have been reported to be involved in different aspects of root development in Arabidopsis as well as other crop species. Other criteria that we used to narrow down the number of candidate genes was to test for their variation in gene expression if the candidate genes colocalized or overlapped with the root trait QTL from previous studies in soybean. Both of these criteria allowed us to narrow down our list of candidate genes to a manageable number, especially for those genes whose expression was quantified in lines carrying the favorable or unfavorable alleles for specific root traits.

**Fig. 6.**
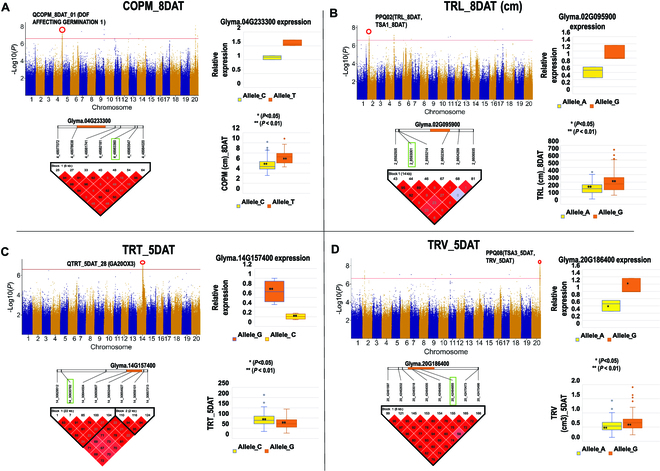
Putative candidate genes. (A) Glyma.04G233300 is a putative candidate gene positively controlling center of mass (COPM)*.* A Manhattan plot showing associations and location of the GWAS peak for COPM_8DAT is enclosed by a red circle along with the putative candidate genes tested for gene expression. The Haploview graph shows the LD block of 6 kb defined by the Hardy–Weinberg equilibrium test for linkage disequilibrium, and the pairwise *R*^2^ values for correlation coefficient are also presented in the figure, which shows the significantly associated SNP (in green box) and the putative candidate gene (orange bar). The level of expression for the candidate gene, *Glyma.04G233300*, in LD with the GWAS peak on chromosome 4 for center of projected mass (COPM_8DAT) was higher in the lines carrying the favorable allele (T) and lower in lines with the unfavorable allele (C). This observation was positively correlated with the associated trait in lines carrying contrasting alleles, and soybean lines with the favorable allele (T) had a higher mean value for COPM_8DAT. (B) Glyma.02G095900 is a putative positive regulator of total root length and surface area of thinner roots. A Manhattan plot depicts associations and location of the GWAS peak for TRL_8DAT enclosed by a red circle with the putative candidate genes whose gene expression was measured. The Haploview graph shows the LD block of 14 kb defined by the Hardy–Weinberg equilibrium test for linkage disequilibrium, and the pairwise *R*^2^ values for correlation coefficient are presented in the figure, which shows the significantly associated SNP (green box) and the putative candidate gene (orange bar). The level of expression for the candidate gene, *Glyma.02G095900*, in LD with the GWAS peak on chromosome 2 for total root length (TRL_8DAT) and surface area of fine roots (TSA1_8DAT) was correlated with phenotypic variation for this trait, with lower gene expression in accessions carrying the unfavorable A allele compared to accessions carrying the favorable G allele. The accessions carrying the A allele had significantly smaller TRL_8DAT and TSA1_8DAT values than the accessions carrying the G allele. (C) Glyma.14G157400 is a putative negative regulator of total number of lateral roots. The Manhattan plot shows associations and location of the GWAS peak of TRT_5DAT enclosed by a red circle with the putative candidate genes whose gene expression was quantified. The Haploview graph shows the LD block of 22 kb defined by the Hardy–Weinberg equilibrium test for linkage disequilibrium, and the pairwise *R*^2^ values for correlation coefficient are presented in the figure, which shows the significantly associated SNP (green box) and the putative candidate gene (orange bar). The level of expression for the candidate gene, *Glyma.14G157400*, in LD with the GWAS peak on chromosome 14 for total number of root tips (TRT_5DAT) was correlated with phenotypic variation for this trait, with lower gene expression in accessions carrying the favorable C allele compared to accessions carrying the unfavorable G allele. The accessions carrying the G allele had a significantly smaller number of root tips in its root system than the accessions carrying the C allele. (D) Glyma.20G186400 is a putative positive regulator of total root volume and surface area of thicker roots. A Manhattan plot depicts associations and location of the GWAS peak of TRV_5DAT enclosed by a red circle with the putative candidate genes quantified or the gene expression. The Haploview graph shows the LD block of 8 kb defined by the Hardy–Weinberg equilibrium test for linkage disequilibrium, and the pairwise *R*^2^ values for correlation coefficient are presented in the figure, which shows the significantly associated SNP (green box) and the putative candidate gene (orange bar). The level of expression for the candidate gene, *Glyma.20G186400*, in LD with the GWAS peak on chromosome 20 for TSA of thicker roots (TSA3_5DAT) and total root system volume (TRV_5DAT) was correlated with phenotypic variation for these 2 traits with higher *Glyma.20G186400* expression in accessions with the favorable G allele, compared to accessions carrying the unfavorable A allele. The accessions carrying the A allele had a significantly lower root volume and surface area of thicker roots.

Finally, qRT-PCR gene expression analysis was conducted on the 4 (2 of 4 potentially pleiotropic) most promising candidate genes that were associated with the root traits COPM_8DAT, TRL_8DAT and TSA1_8DAT, TRV_5DAT and TSA3_5DAT, and TRT_5DAT (see Table S10 for physical position, allelic variation, and the effect of the significantly associated SNPs on these traits). For a major COPM_8DAT GWAS peak, the candidate gene, DOF1-like zinc finger transcription factor, DAG1 (DOF affecting germination 1) (*Glyma.04G233300*), and the significantly associated SNP were in the same linkage block of 6 kb on chromosome 4. The expression of *Glyma.04G233300* was higher in the soybean lines with larger COPM values (distance of the root center of mass from the root/shoot junction, usually associated with deeper root systems), which carry the favorable allele, T, whereas this gene’s expression was lower in lines with the unfavorable allele, C (Fig. 6A). The second candidate gene that was associated with TRL_8DAT and TSA1_8DAT for a GWAS peak on chromosome 2 (*Glyma.02G095900*) was an uncharacterized protein, which was in the same LD block (14 kb) and located 4 kb from the significantly associated SNP. Results showed that its level of gene expression was higher in accessions carrying the favorable G allele (higher trait value) and lower in genotypes with the unfavorable A allele (lower trait values) for the most significant SNP (Fig. 6B). Gene expression of another candidate gene for the trait TRT, *Glyma.14G157400* (GA20OX3), although located 20 kb from the GWAS peak, was in the same LD block (22 kb) as the SNP on chromosome 14. The expression of *Glyma.14G157400* was lower in accessions carrying the favorable C allele (higher TRT_5DAT) compared to those accessions carrying the unfavorable G allele (lower TRT_5DAT) (Fig 6C). This suggests that *Glyma.14G157400* is a negative regulator of lateral root number. Finally, a candidate gene for total TRV_5DAT and TSA3_5DAT of thicker roots, *Glyma.20G186400* (C2H2 zinc finger protein) on chromosome 20, and a genic SNP detected as a pleiotropic GWAS peak, PPQ08, was evaluated for differential expression in genotypes with favorable (G) and unfavorable (A) alleles. Genotypes with accessions carrying the favorable G allele had significantly higher expression than the genotypes with the unfavorable A allele (Fig. 6D).

#### Structural variation in the promotor region of candidate genes

We investigated structural variations in the promotor region of the 4 most promising candidate genes due to the variation in relative expression of these genes in favorable and unfavorable haplotypes. Out of the 4 candidate genes, only the promoter region of one candidate gene had a large indel consisting of 7 pairs of TA repeats [14 base pairs (bp)] at 275 bp upstream of *Glyma.02G095900.* The possibility that this indel is involved in the differential expression of *Glyma.02G095900* may be part of our future research emerging from this study.

#### Distribution of traits of interests among subpopulations based on structural analysis

Apart from the significant overall genetic variation for root morphological and architectural traits, distribution of RSA traits among subpopulations (closely aligned with country of origins) was not random for 3 of the 4 traits of interests (Fig. S13) that harbored our 4 highest priority candidate genes. As described above, these 4 genes had been examined for variation in gene expression between the lines with superior and inferior alleles (Fig. 6). COPM_8DAT and TRL_8DAT had the highest mean values (6.25 and 265 cm, respectively) in subpopulation group 2, which was enriched with soybean accessions belonging to east Asian countries of origin. By comparison, subpopulation group 1 that is enriched with Chinese and Russian accessions had the lowest mean values for these 2 traits. For TRT_8DAT, subpopulation groups 5 and 4 had highest and lowest mean values, respectively. Interestingly for TRV_5DAT, the results did not show statistically significant differences among subpopulations (Fig. S13).

### Phenotypic validation of root COPM in plants grown in potting mix at the R1 stage of soybean plant growth

**Fig. 7.**
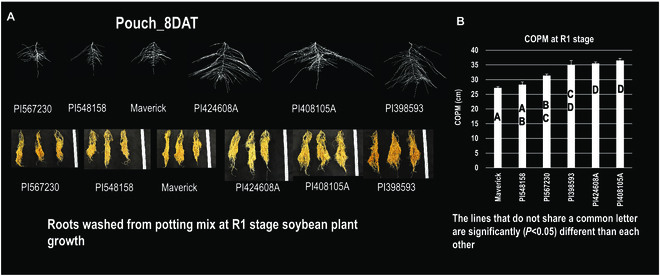
Phenotypic validation of root COPM in plants grown in potting mix at the R1 stage of soybean plant growth. (A) Six soybean lines, 3 with lower and 3 with higher values of root traits associated with root system size and depth (TRV, TRL, and COPM), were grown in the pouch system and imaged for RSA at 8 DAT.(B) Validation of 2D pouch results for root systems of plants grown in potting mix until they reach the reproductive stage R1 for the 6 soybean genotype representing lines with contrasting COPM (3 with smaller COPM and 3 with larger COPM). Tukey’s honestly significant difference test for multiple comparison was conducted to define the statistical significance for the difference in COPM among genotypes. Soybean genotypes that do not share a common letter are statistically significantly different (*P* < 0.05) from each other for COPM trait. The difference in root system size and depth between each set of 3 soybean genotypes correlated closely between pouch grown young plants and potting mix-grown older plants grown to the reproductive stage.

To validate that the root system traits grown in the hydroponic pouch system are representative of plants grown for longer periods in soil or soil-like growth media, we grew 4 replicate plants for each of 6 soybean lines contrasting for COPM [3 lines with low COPM (shallower roots) and 3 lines with the highest COPM (deeper roots)]. These same lines also contrasted for TRV and TRL, which are measures of the size of the entire root system. The plants were grown until the R1 reproductive stage (approximately 45 days of growth) in the greenhouse. At the end of the growth period, the potting mix was carefully washed from the root systems of the contrasting lines and the root systems were imaged and quantified for COPM. As shown in Fig. 7A where the root systems for the 6 lines are depicted after growth in the pouches (13-day-old plants) and also in the potting mix in pots (45-day-old plants), it is clear that the lines categorized as deeper and larger rooted in the pouches exhibited the similar differences compared to the smaller and more shallow rooted genotypes when grown in potting mix for 45 days. Quantification of the COPM trait in the potting mix-grown plants is depicted in Fig. 7B, where the results showed statistically significant differences in COPM between the deeper/larger and shallower/smaller root systems grown for 45 days. Four of these 6 lines had contrasting alleles for COPM (2 lines for each allele), and the difference in COPM in these lines agreed with the difference in expression of the candidate gene for COPM *Glyma.04G233300* for this trait.

## Discussion

Root system traits are often complex and relatively understudied because of their growth in opaque, heterogeneous, and complex soils. But the root system’s importance to the plant is undisputed due to its essential roles in nutrient and water acquisition, abiotic and biotic stress response signaling, and anchoring and stabilizing the plant in the soil. Therefore, a more in-depth understanding of the genetic and molecular regulation of root growth, development, and functional traits is of utmost importance to improve crop adaptation to marginal soils and climate change. This is necessary to develop crop varieties with more efficient root systems that can lead to enhanced yields in both favorable and stressed environments. A major role for root systems that has become apparent over the past several decades is the importance of RSA in more efficient water and fertilizer acquisition. It has been shown that macronutrient P (as the phosphate anion), which moves slowly in the soil because it tend to be fixed to soil particles, is efficiently harvested by the shallow and more extensive root systems, with longer, thinner, and shallower lateral roots, with more root hairs, and in general with greater root surface areas in the topsoil. This trait is often termed P acquisition efficiency. Furthermore, the P acquisition efficiency gene, *PSTOL1*, which was first discovered in rice [12], confers improved performance on low P soil through modification of RSA, leading to larger and more extensive root systems. The link between RSA and *PSTOL* function has been best shown in sorghum, where Hufnagel et al. [9] showed significant statistical associations for grain yield on low P soils in the field (P efficiency) with longer and thinner lateral roots, and several sorghum *PTOL1* genes, validating these genes’ role in sorghum P efficiency. For fast-moving nutrients like water and nitrate, it has been shown that deeper rooting can confer drought avoidance and improve yields under drought and lower N levels, by accessing deeper groundwater and NO_3_^−^. A major gene that helps confer deeper lateral rooting, *DRO-1*, has been identified in rice and shown to confer yield increases under moderate and severe drought in the field [7].

Because of the importance of RSA to nutrient and water acquisition efficiency, there has been considerable interest in developing relatively sensitive root imaging methodologies that have the throughput to conduct genetic analysis of root growth and architecture traits. Certainly, one of the difficulties in studying RSA is the labor and time it takes to phenotype roots in the field. The primary approach, shovelomics, where usually primarily the upper portion of the root system is excavated from the soil, has the strength of providing and quantifying root traits in their in situ soil environment. However, the excavation of the root system is destructive and disruptive to the RSA, often with loss of deeper and finer roots. Furthermore, this approach is labor intensive and has relatively low throughput. The other type of approach is to grow the roots in artificial and transparent growth media, such as hydroponics, gels, or hydroponic-fed 2D pouches that have a transparent root viewing plate. In this study, we made some significant improvements in the often-used root growth pouch systems as described in Results to phenotype root growth and 2D RSA traits in more than thousand plants from a 286 soybean line association panel assembled from the USDA germ plasm collection by the Soybean Genetics and Genomics Lab, University of Missouri, USA. This enabled us to have the throughput to phenotype 5 replicate plants for each of the 286 lines, with the precision and accuracy to reproducibly quantify root traits for subsequent GWAS analysis, and also the identification and characterization of some root trait candidate genes. For example, image segmentation and the cleaning pipeline to count number of root tips more precisely by removing the background noise caused by the small particles might have played an important role directly in the identification of an important candidate gene for a number of lateral roots (TRT), GA20OX3.

Also, a false-positive number of root tips can indirectly affect total root length and volume traits as these traits are interrelated; hence, this pipeline can lead to digital computation of root traits that are closer to realistic values and therefore help in identification of causative genetic variants for these traits with better resolution.

**Fig. 8.**
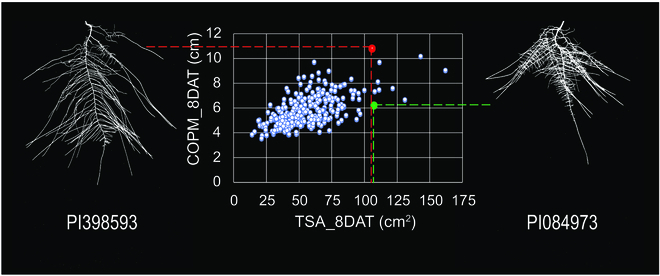
Two soybean genotypes with similar root morphology but variable architectural traits. Plot of the root architectural trait, center of mass, as a function of the root morphological trait, root surface area. This plot clearly shows that even though the 2 accessions, PI084973 and PI398593, have very similar total root system surface areas (TSA_8DAT), they are quite different in rooting depth with the distance of the root center of mass (COPM_8DAT) in PI398593 from the root:shoot junction about 50% larger than in PI084973.

We observed a large amount of root growth and RSA phenotypic variation among soybean genotypes, subpopulation groups, and maturity groups. Early maturity group accessions were found to exhibit greater early season root growth compared to late maturity groups. Larger root morphological and architectural trait values for early maturity group lines compared to late maturity group lines suggest that higher root growth rate in the earlier stages of plant growth is advantageous for shorter season soybean lines. These results are similar to those found on root systems of early and late maturing mung bean reported by Singh and Bell [51]. Our findings with soybean suggest that early maturity accessions try to establish extensively proliferated root systems in earlier stages of their life cycle to explore maximum soil area, as demonstrated by having larger convex hull area, root system center of mass, and larger fine root TSA, which is beneficial in acquiring water and nutrients as soon as possible during establishment. On the other hand, the longer growing season late maturity lines have more time to establish root systems and acquire water and nutrients over the longer time of growth, which can enable more rainfall events during the growing season. Additionally, we found that the late maturity lines had a set of thicker roots in their system, which should enable them to have better anchorage over the longer growth period. We also observed from root system phenotyping that different soybean genotypes with similar morphological traits can have very different root architectures. For example, accession number PI398593 had significantly higher root system center of mass than PI084973; however, both accessions had similar total root system surface areas (Fig. 8). We have found a number of examples like this that strongly suggest that although root growth/morphological traits can predict the performance of the root system to some degree, architectural traits such as deeper center of mass can enhance the prediction of root system performance in response to abiotic stresses such as drought.

### GWAS on soybean root growth and architecture traits generated several genomic regions of interest for further study

GWAS of our set of root traits identified putative genetic variants that underlie a portion of the phenotypic variation in root system growth/morphological and architectural traits (Figs. S9 and S10). The majority of significant loci associations for architectural traits were independent of and did not colocalize with significant peaks for root growth/morphology traits. This observation suggests that although many architectural traits are presumably genetically complex and controlled by multiple genes, there are quite a few architectural traits that are regulated by independent loci, such as deep rooting and drought avoidance by *DRO-1* in rice [7] and P acquisition efficiency in rice and sorghum by *Pstol1* [9,12]. Similarly, in this study, some of the individual GWAS loci appear to have a significant effect on changes in RSA. Therefore, this observation warrants more detailed analysis in order to better understand their genetic and physiological basis and to be utilized for breeding for improved root traits. Colocalization of approximately 58% of the significant GWAS associations from our study with previously identified root trait QTLs in soybean biparental mapping studies certainly helps support the validity of many of the associations identified in this study. Further, the application of high-throughput hydroponic phenotyping of root systems is shown here to be a useful approach to identify genetic variants responsible for the differences in root growth and architecture, as our group has used it previously to identify root genes conferring P efficiency and aluminum tolerance [9,55]. For 2 root system architectural traits, SOL and TRW, we could only find 33% and 0%, respectively, of the GWAS peaks colocalized with previously published root trait genetic studies. It makes sense as most of previous studies have been focused on root morphological traits and these results further support the argument that it is important to include the phenotyping of root architectural traits along with root morphological traits.

Interestingly, significantly associated SNPs with lower allele frequencies seem to be relatively commonly shared among different root trait mapping studies. A root length association in this study (QTRL_8DAT_5, MAF 0.06) colocalized with root length, area, weight, and lateral root density QTL from the Liang et al. [33] study and with root diameter mapping from Van Nguyen [35]. In another case, root volume associations from our study (QTRV_5DAT_08, MAF 0.05) colocalized in other studies with primary root width and lateral root density [30], dry root weight [32], and root volume [14].

### Four highest priority candidate genes associated with root center of mass (COPM), total root length (TRL), total number of lateral roots (TRT), or root volume (TRV)

#### *Glyma.04G233300*: A putative positive regulator of root center of mass (COPM)

As described above, *Glyma.04G233300* encodes a DOF1 transcription factor, DAG1, that is very closely located to a GWAS peak for variation in COPM, which is a measure of the depth of the entire root system. Allele analysis suggests that it could be a positive regulator of COPM (Fig. 6A). DAG1 has been reported to negatively regulate gibberellic acid (GA) biosynthesis genes. For example, Gabriele et al. [56] reported up-regulation of the GA biosynthesis gene, *AtGA3ox1*, in the *dag1* mutant in Arabidopsis. GA mediates various plant (shoot and root) developmental processes and has been suggested to negatively regulate the number of lateral roots in a root system [57,58]. Furthermore, association peaks for the COPM trait in our study colocalized with a soybean QTL for the number of lateral roots [32]. These observations suggest that the DAG1-GA network might be regulating the depth of the root system COPM trait at this locus. In another case, cotton lines overexpressing *GhDOF1* had a greater total root system volume and surface area as well as increased tolerance to NaCl toxicity, based on root growth inhibition in hydroponic media containing 150 mM NaCl.

#### *Glyma.14G157400*: A putative negative regulator of a number of lateral roots

The candidate gene, *Glyma.14G157400*, on chromosome 14, encodes the enzyme, GA 20 oxidase 3. GA oxidases have been reported to control the number of lateral roots via crosstalk with auxin [57,59]. As mentioned above, gibberellins impact various aspects of plant growth and development (reviewed by Fleet and Sun [60]). A GA-deficient mutant in tomato plants has been reported to have an increased number of lateral roots compared to wild-type (WT) due to GA-mediated changes in carbon partitioning between shoot and roots [61]. Della proteins have been reported to mediate responses to GA. Populus mutants expressing GA response regulators lacking their DELLA domains, gai and rgl1-3, had a greater number of lateral roots due to blockage of response to GA [58]. Further, GA-deficient transgenic plants in poplar exhibited similar results, suggesting again that a GA is involved in negative regulation of a number of lateral roots [59]. Recently, Wu et al. [62] discovered that mepiquat chloride was an inhibitor of GA synthesis, and cotton seedlings treated with this compound had reduced expression of GA20 oxidase and enhanced number of lateral roots. This GWAS peak is significantly associated with the GA20 oxidase gene. Furthermore, a soybean root biomass QTL identified in the work of Zhang et al. [54] further support our suggestion that *Glyma.14G157400* might be a negative regulator of lateral root number based on our observation of an inverse relationship between *Glyma.14G157400* expression and lateral root number (Fig. 6C). Finally, Prince et al. [14] identified an MRE (metal responsive element)-binding transmembrane protein of unknown function (Glyma.16G141800) as a candidate gene that regulates the number of lateral roots. In that case, they also observed that an increased concentration of hormones, in this case IAA, and a significantly higher concentration of ABA in soybean plants were associated with the unfavorable haplotype (lower number of lateral roots). These findings again suggest roles for hormonal levels and interactions between plant hormones such as auxin and GA in the regulation of lateral root formation.

#### Glyma.02G095900: A putative positive regulator of total root length and surface area of thinner roots

*Glyma.02G095900* is an uncharacterized protein, and its Arabidopsis ortholog, *TRM18* (*Tonneau/TON 1 recruiting motif 18*), is a phosphatidylinositol *N*-acetylglucosaminyltransferase subunit P-like protein. TRMs (TON1 recruiting motif proteins) have not been reported in root development but have been reported to play a role in cortical microtubule organization. TRMs recruit TON1 to form the TTP (TON1-TRM-PP2A) complex by binding protein phosphatase 2A, and this complex has been reported to regulate arrangement of cortical microtubules arrays, and thus be involved in cell division plane formation and cell growth, and the shape of plant organs [63–65]. Additionally, PP2A has been reported to regulate primary root length, and double mutants for *pp2a* (*pp2a-3*/*pp2a-4*) have defects in the organization of root tip cells and shorter primary roots compared to WT Arabidopsis plants [66]. Also, recently, it was reported that a phosphatidylinositol N-acetylglucosaminyltransferase II enzyme positively regulated root growth in Arabidopsis because a knockout for the gene encoding this enzyme, *GnTII*, exhibited reduced root growth compared to WT Arabidopsis plants [67]. Variation in expression of *Glyma.02G095900* (Fig. 6B) might be due to the deletion of TA repeats in the promotor region (data not shown). TATA sequences are known to be able to bind certain transcription factors and regulate gene transcription. Further characterization of the function in transcription of this indel is needed to confirm this hypothesis using soybean transgenic hairy roots with promoter::reporter gene assays.

#### Glyma.20G186400: A putative positive regulator of total root volume and surface area of thicker roots

*Glyma.20G186400*, a candidate gene for changes in total root volume and surface area of thicker roots, was identified on chromosome 20 and is a Cys^2^/His^2^ zinc finger protein named DEFECTIVELY ORGANIZED TRIBUTARIES 5 (DOT5). The Arabidopsis *DOT5* homolog harbors a WIP domain, which consists of 4 zinc finger motifs and is involved in the regulation of venation in leaves as well as root development [68]. Short roots in the *dot-5* loss-of-function mutant are consistent with DOT5’s putative positive regulation of root growth in our results (Fig. 6D). Petricka et al. [68] further speculated that the DOT5 protein is involved in the regulation of auxin efflux carriers due to enhanced sensitivity of auxin transport inhibitors in the *dot5* mutant. The C2H2 zinc finger family is one of the largest families of transcription factors involved in the regulation of plant development processes and response to abiotic stress responses [69,70]. Recently, it was shown that heterologous expression of a C2H2 zinc finger protein from a wild relative of soybean (*Glycine soja*) into Arabidopsis resulted in improved aluminum (Al) tolerance with greater root growth in Al-toxic media compared to WT Arabidopsis plants [70].

### Breeding prospects for the use of identified GWAS peaks tightly linked to RSA candidate genes

Drought and nutrient deficiency are the 2 major limitations of soybean yield [71–74]. The total number of lateral roots or number of root tips in a soybean root system contributes significantly to total root length and surface area and has been reported to be the characteristic of RSA of soybean lines (as well as other crop species in general) with greater yield under drought field conditions [14,71]. It is easy to see why a greater number of lateral roots, which translates into greater root system surface area for water and mineral nutrient uptake, would translate into improved water and nutrient acquisition efficiency [75]. We are finding similar results for root studies we are doing in canola and sorghum (unpublished data). As root volume is a trait that is roughly equivalent to root biomass, it is another root trait that is relatively easy to quantify, significantly contributing to improved performance under drought as it is also influenced by the number of lateral roots, root length, and root surface area [29,76]. Pyramiding of desired alleles from different root traits to shape a more efficient nutrient-acquiring RSA could lead to the production of crop varieties more resilient to climate change and make a significant contribution to agricultural sustainability, with improved yields using less N-P-K fertilizers.

## Conclusions

In this study, we were able to capture a significant portion of the large genetic diversity underlying the phenotypic variation for root architectural and growth/morphological traits. Our results suggest that a large number of GWAS peaks for root system architectural traits except total convex area were generally independent of root morphological traits. This strengthens the idea of incorporating and mapping of more root architectural traits that might contribute to higher yield and abiotic stress tolerance in resource-limited environments, including drought and lower levels of mineral nutrients. Nevertheless, the importance of root morphological traits has been paramount from the early years of root system research as traits such as increased root length, surface area and volume, and development of longer and thinner roots increase the absorption capacity of the root systems while reducing the carbon cost for those root systems. Given the high complexity of root architectural traits, identification of genetic variants controlling root length, volume, and surface area has been the prime focus in most prior research and enabled us to support the previously mapped QTLs and identify novel genetic variants for root morphological traits in this study. For example, a locus significantly associated with total root volume at chromosome 18 was colocalized with root volume or the closely associated trait, root biomass, in 2 previous root biparental studies and one GWAS mapping study. The putative candidate genes we identified in this study, *Glyma.04G233300*, *Glyma.02G095900*, and *Glyma.20G186400*, are possibly involved in positive regulation of center of mass, root length, and root volume, while *Glyma.14G157400* may be a negative regulator of the number of lateral roots in soybean. Differential expression of putative candidate genes tightly linked with these significant GWAS peaks provides further support that possibly one or more of these genes are bona fide root architecture genes and will be the focus of our subsequent research. Also, the genetic markers linked to these genes can be used for the generation of soybean lines with improved and more efficient root systems by marker-assisted breeding or gene editing. Having said that, it is necessary to further validate the effect of the favorable alleles we have identified by introgressing them into elite backgrounds and testing their performance in different field environments, before they can be employed in marker-assisted introgression breeding, genomic selection, or gene editing approaches to improve the RSA of soybean.

## Data Availability

All the data related to this paper are included in the paper and/or the Supplementary Materials.
