# Supplementary material for: Application of an Improved 2-Dimensional High-Throughput Soybean Root Phenotyping Platform to Identify Novel Genetic Variants Regulating Root Architecture Traits
Source: Plant Phenomics. 2023 Sep 28;5:0097. doi: 10.34133/plantphenomics.0097 (PMC10538525; doi:10.34133/plantphenomics.0097)
Supplement: Supplementary 1 — Figs. S1 to S13 Tables S1 to S10 [file plantphenomics.0097.f1.docx]

**Supplementary Figures**


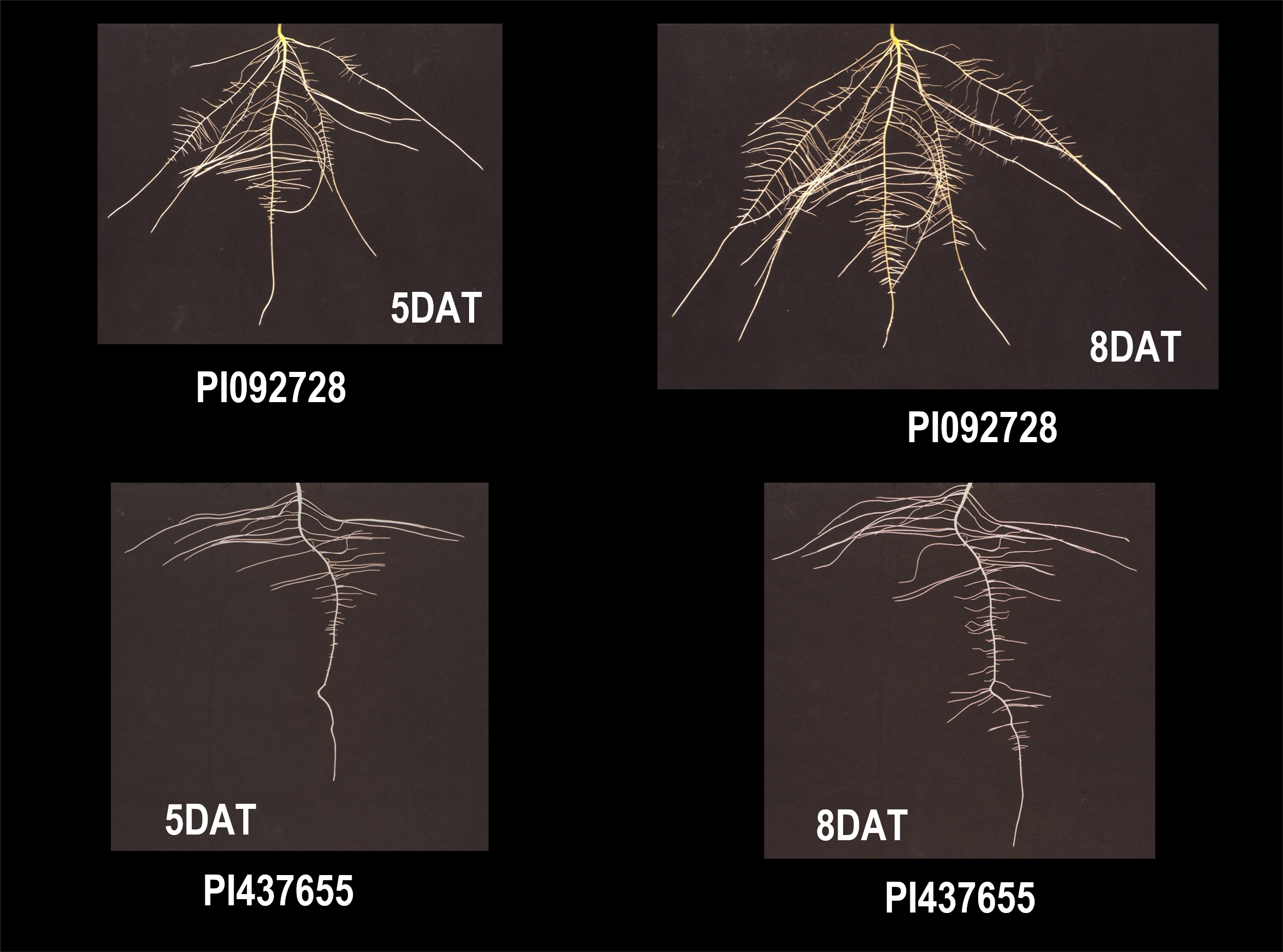


**Figure S1)** Image of soybean roots at 2 growth stages. Representative images of two soybean accessions, PI092728 and PI437655, at 5 days after transplanting into the 2D pouches (5DAT) and at 8 days after transplanting into 2D pouches (8DAT). The seedlings were germinated for 5 days before placing in the pouch, so the age of the seedlings at root imaging are 8 and 13 days.

**
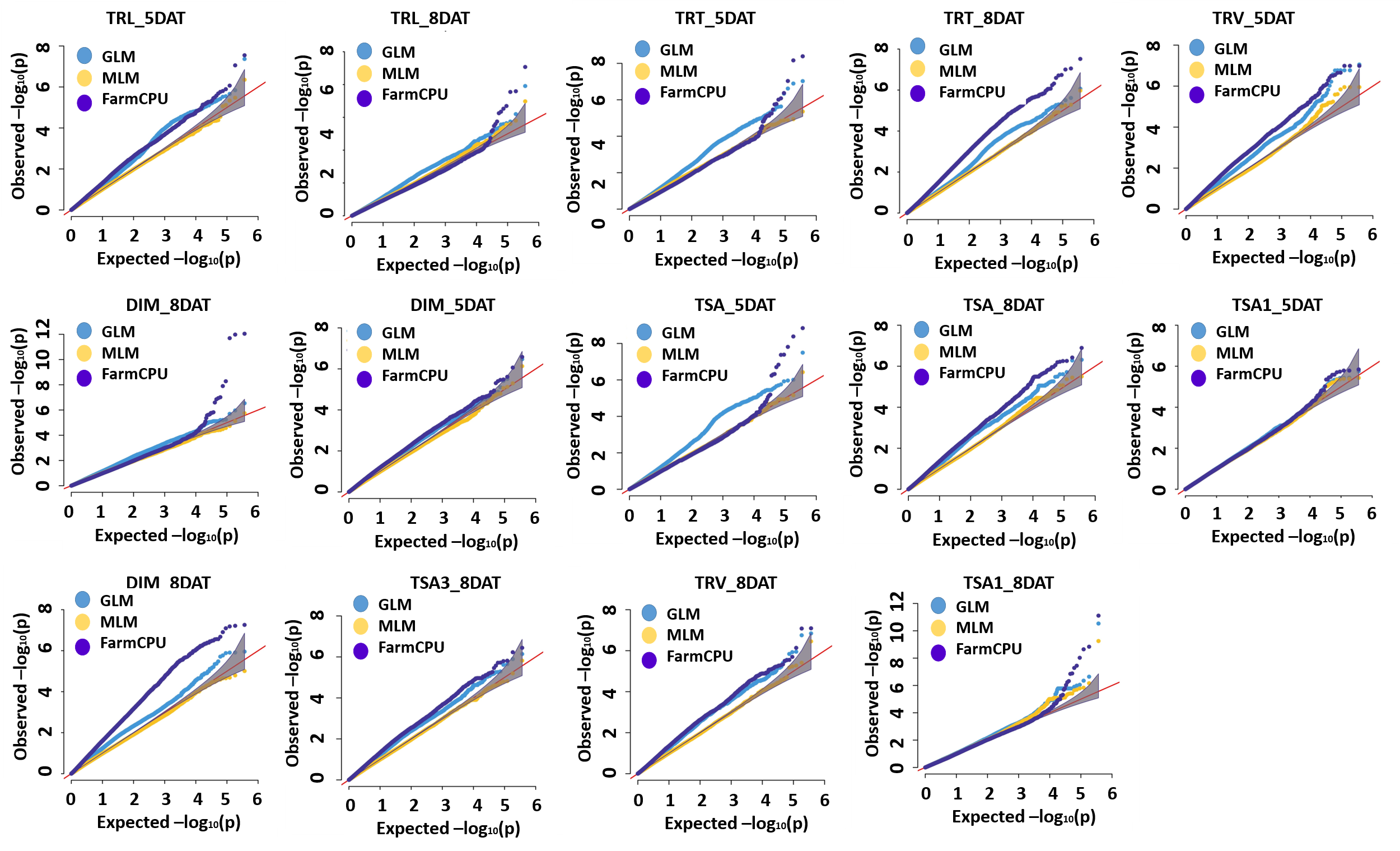
**

**
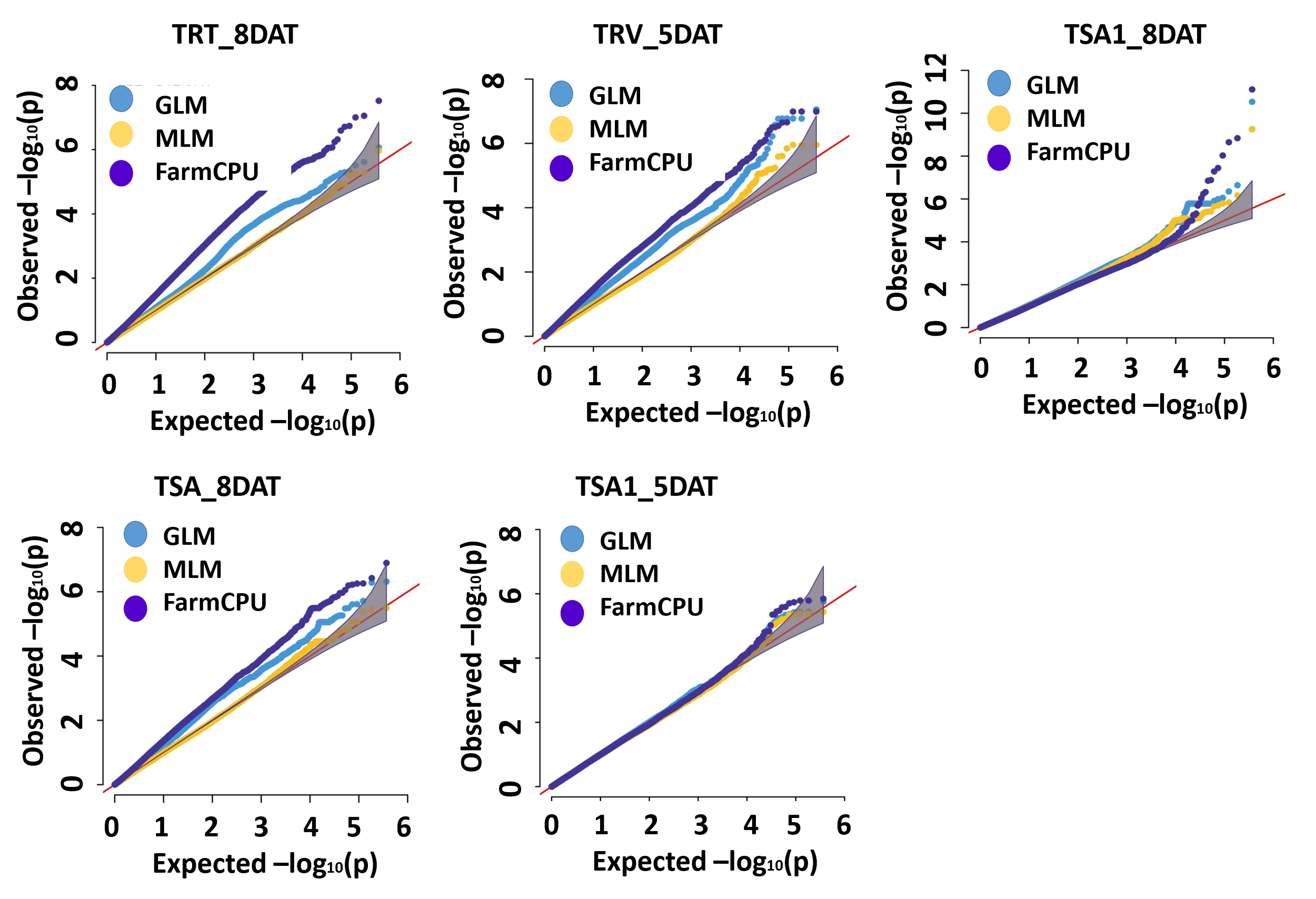
**

**
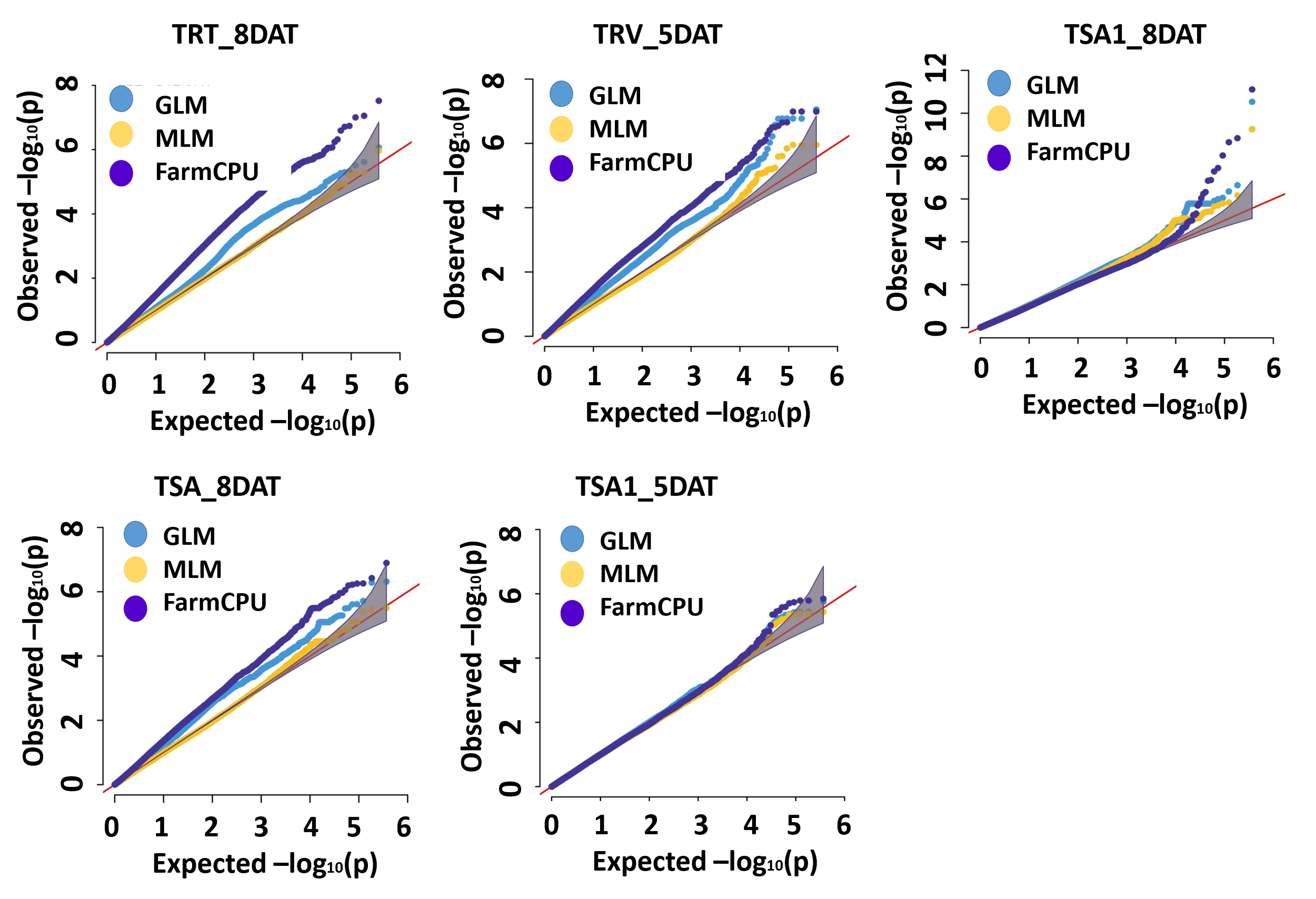
**

**Figure S2)** QQ (quantile-quantile) plots to test the fit of observed with expected results using the GLM (general linear model), MLM (mixed linear model) and FarmCPU (fixed and random model circulating probability unification) model for GWAS on all root morphological traits.

**
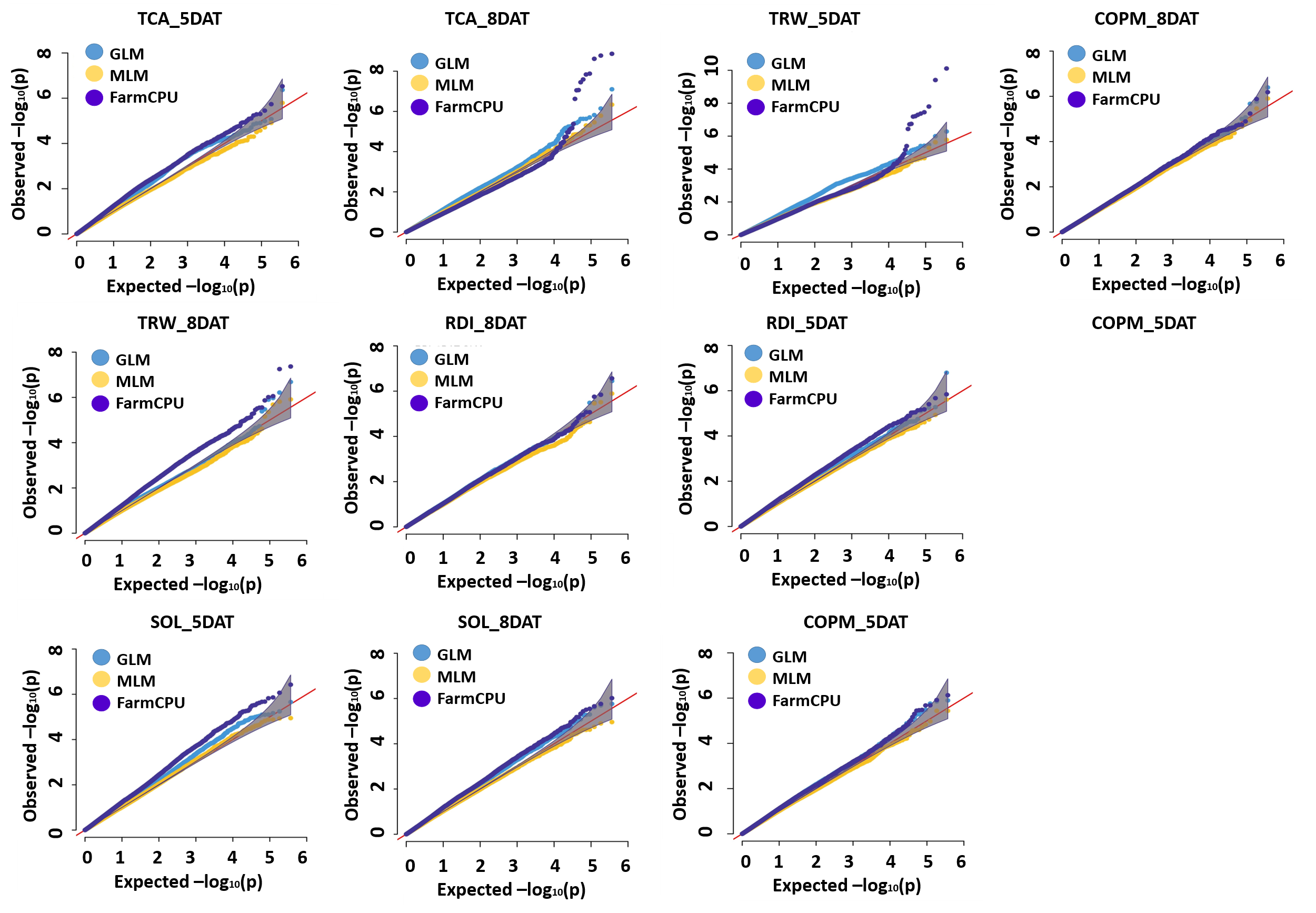
**

**
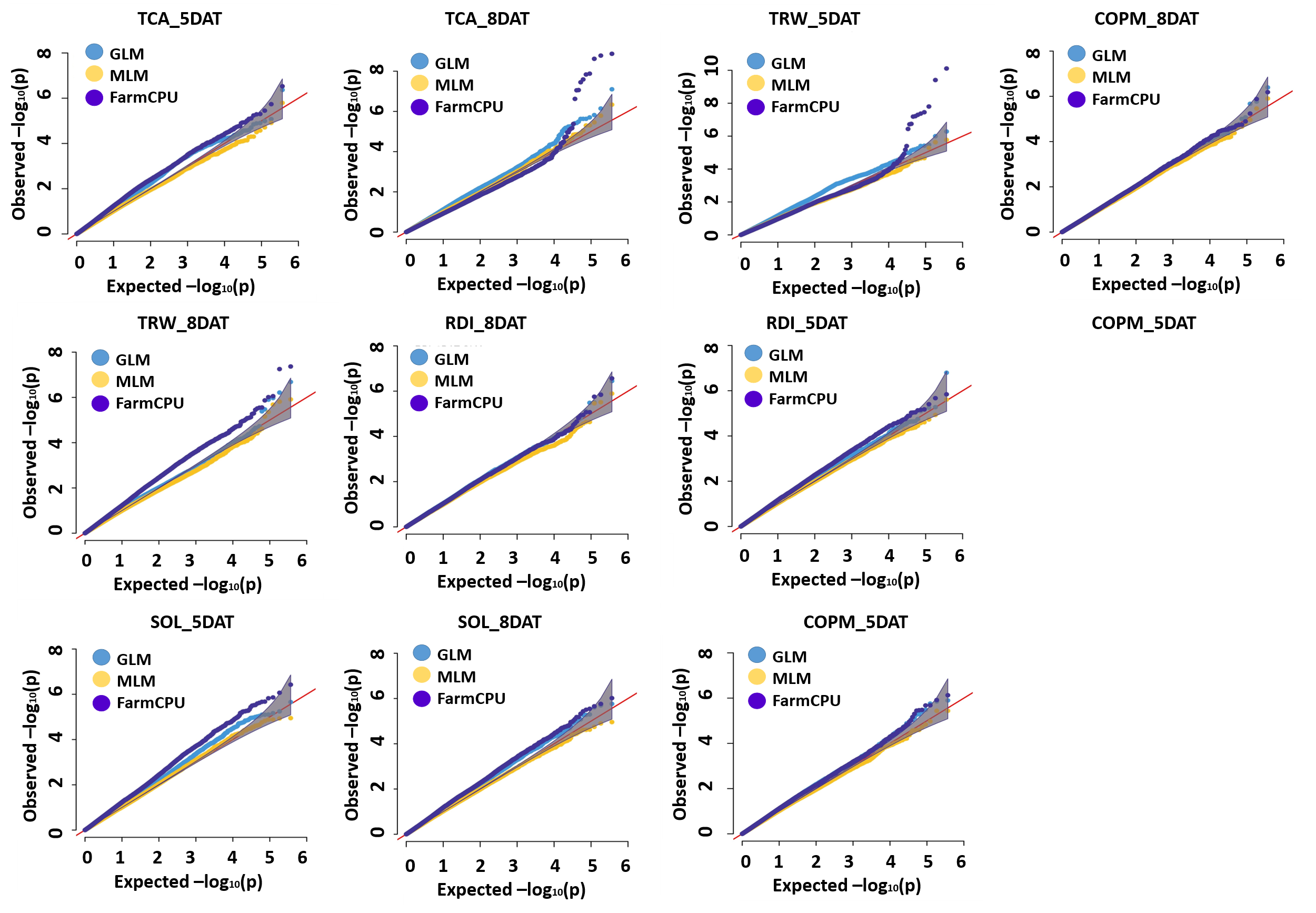
**

**Figure S3)** QQ (quantile-quantile) plots to test the fit of observed with expected results using the GLM (general linear model), MLM (mixed linear model) and FarmCPU (fixed and random circulating probability unification) model for GWAS on all root architectural traits.

**Figure S4)** Delta K = mean(|L’’(K)|) / sd(L(K)) vs K value plot was conducted to carry out population structure analysis. Population structure was analyzed with STRUCTURE software (42) by selecting 12000 evenly spaced genome-wide SNPs. A burning period of 12000 was applied with 10 iterations.


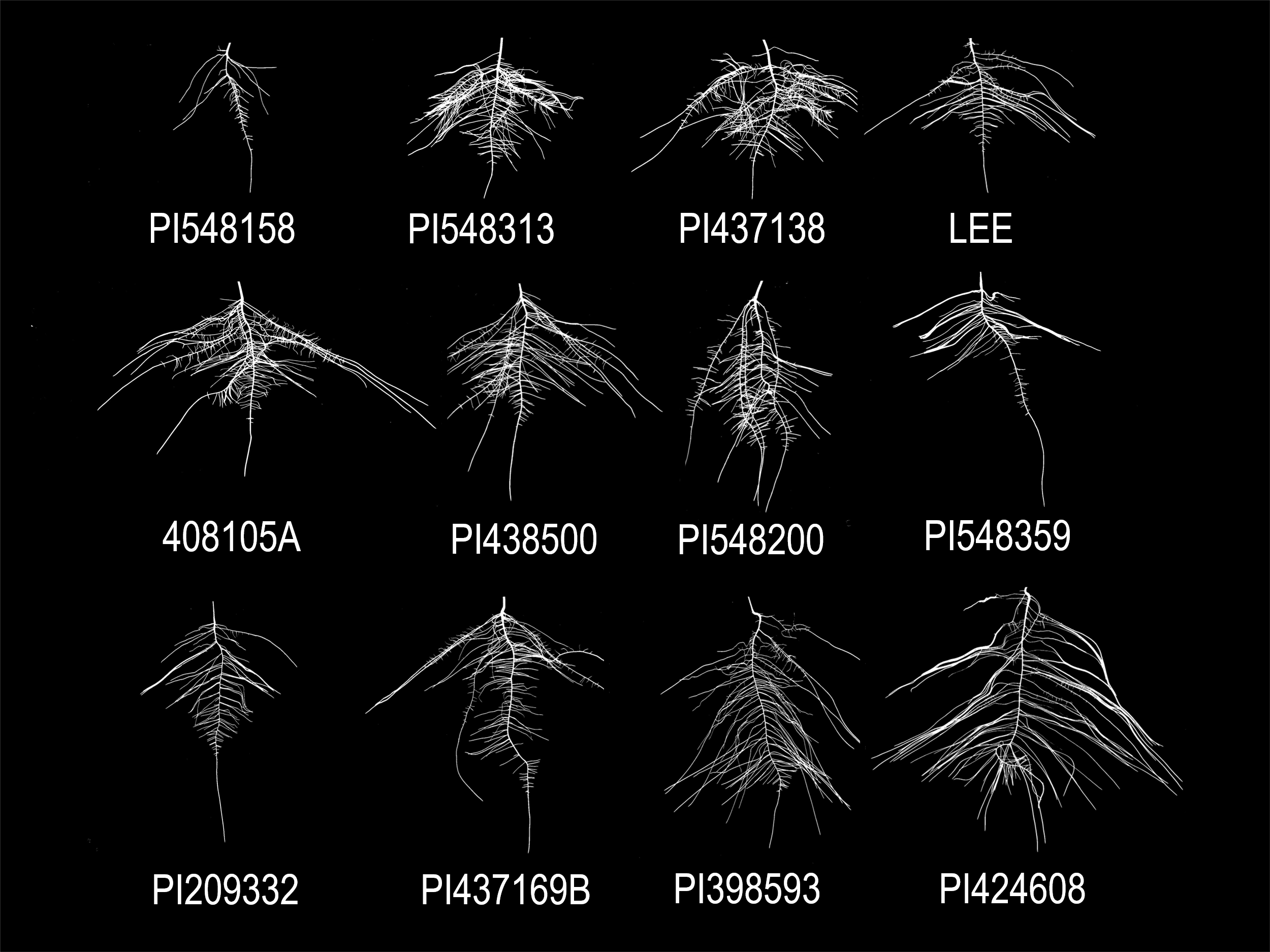


**Figure S5)** **Variation in root system architecture (RSA) in a subset of 12 soybean accessions.** Significant variation is observed in tap root length, lateral root number and length, the distribution of lateral roots at different depths, the width of the root system, and the 2D surface area the root system encompasses.


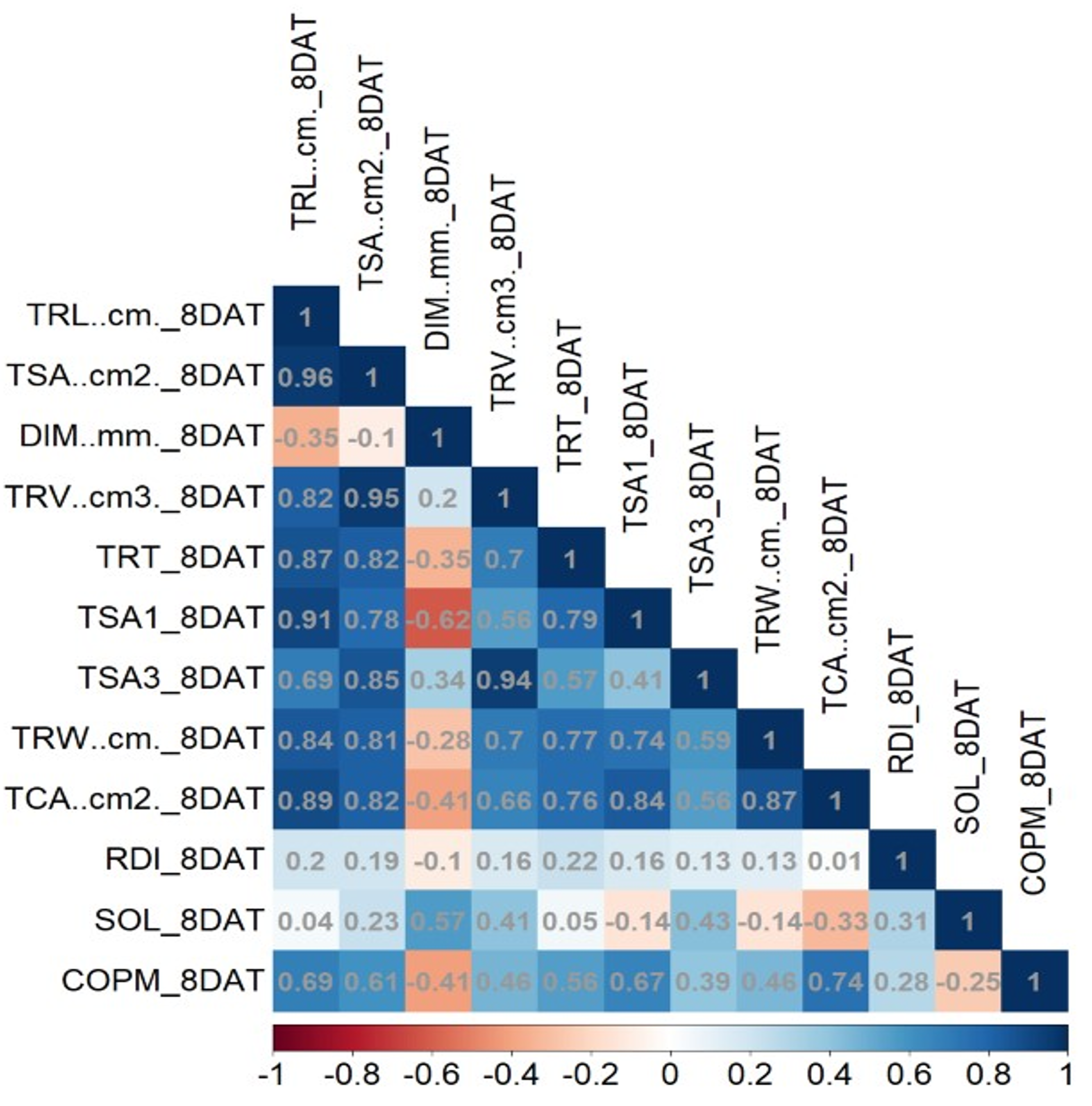


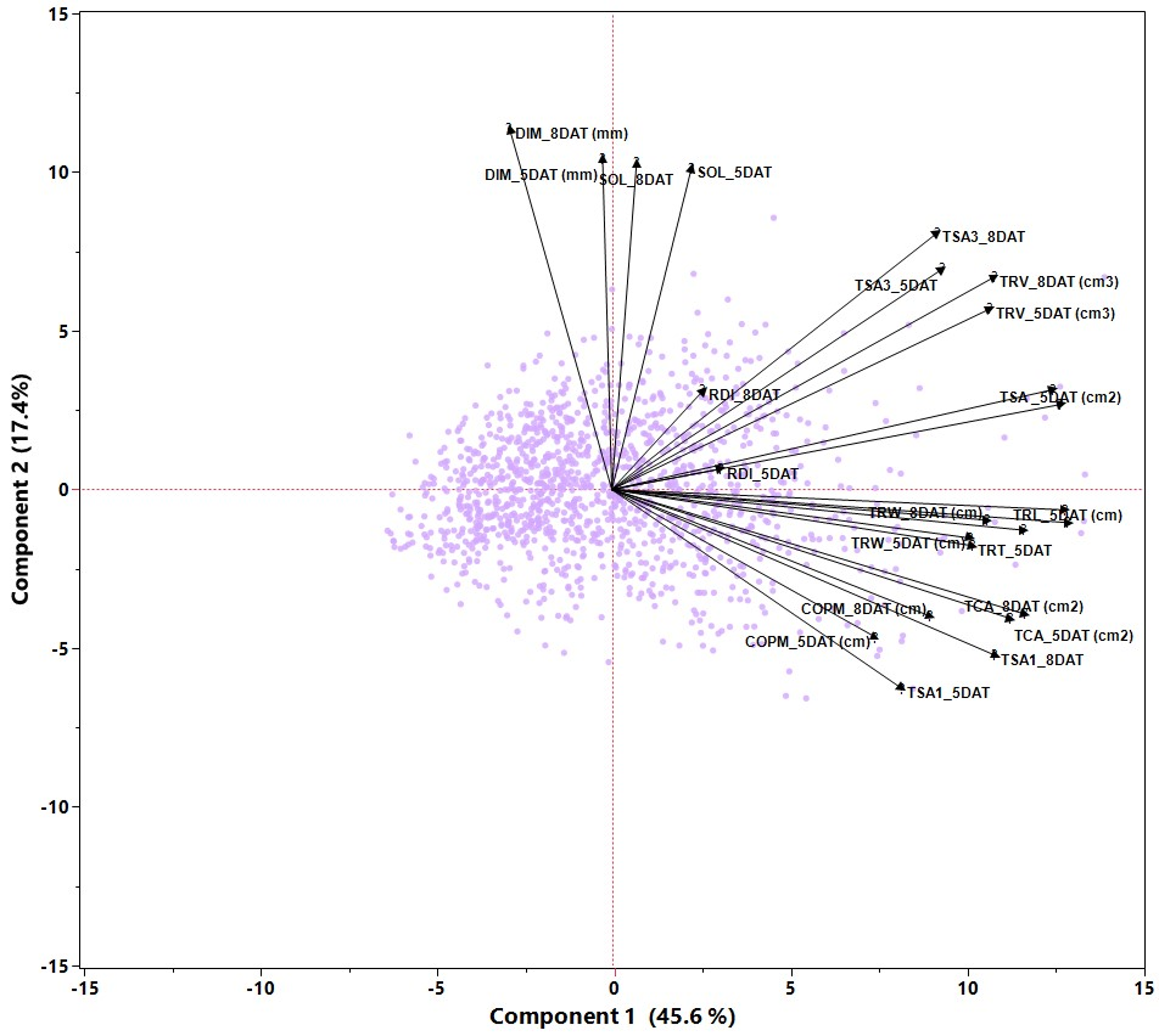


**Figure S6)** Correlation and principal component analysis of root traits. **A**) Color heat map representing strength of correlation between root morphological traits: TRL (total root length), TRV (total root volume), TRT (total number of root tips), DIM (average diameter), TSA (total surface area), TSA1 (surface area of diameter class 0.0 – 0.5 mm), TSA3 (surface area of diameter class 1.0 – 1.5 mm) and architectural traits: TRW (total root width), TCA (total convex area), RDI (root depth index), SOL (solidity) and COPM (center of mass) at 5DAT and 8DAT. Blue and orange colors represent negative and positive correlations, respectively. **B**) Biplot of principal component analysis. Results showed that Component 1 with all the root traits except DIM contributed 50.5 % of the variation in root traits and 17.7% of the variation was contributed by Component 2.


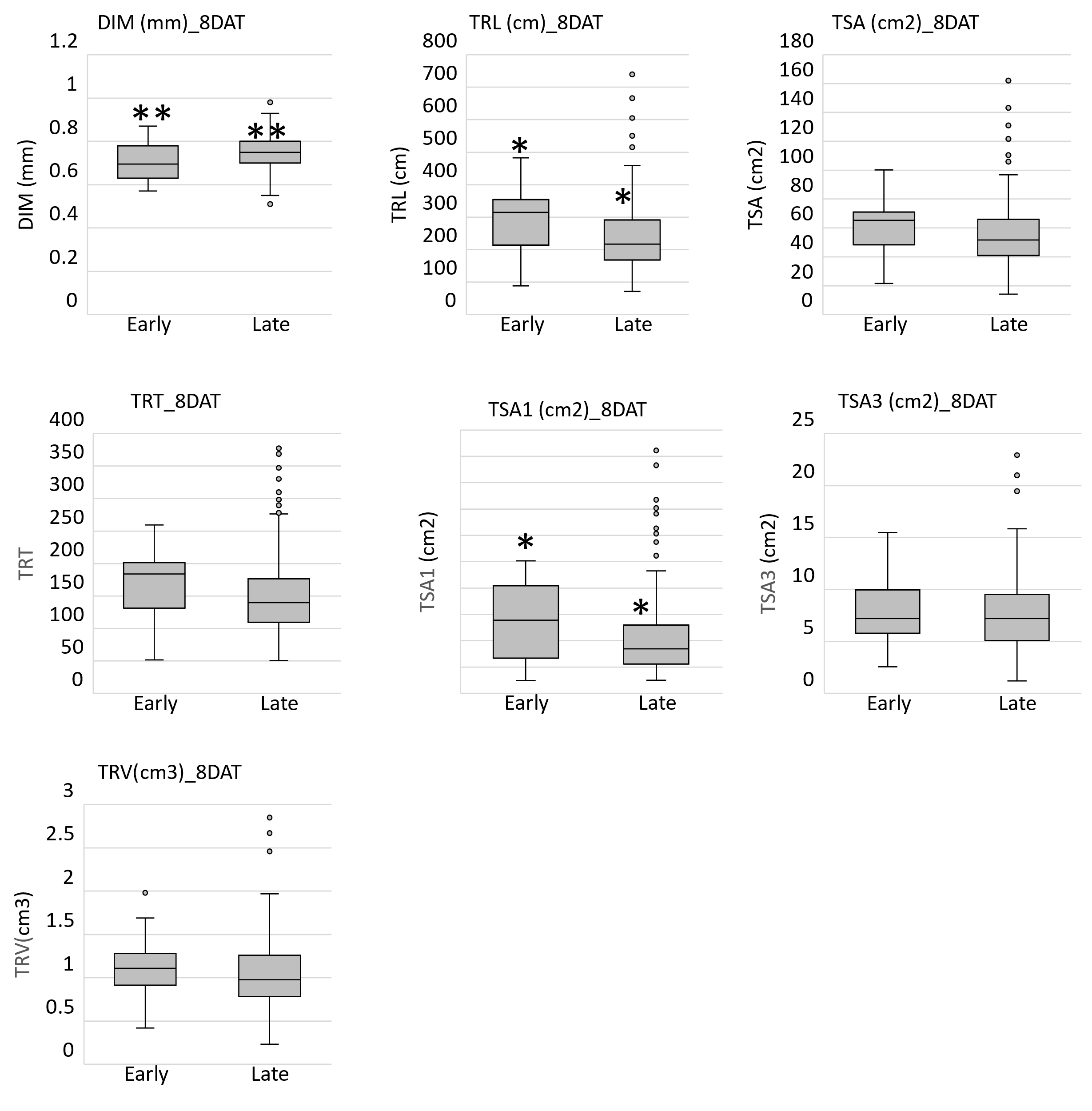


**Figure S7)** Variation in root morphological traits in early maturity (MG0, MGI and MGII) and late maturity groups (MGIII, MGIV, MGV and MGVI) of soybean accessions. The Tukey-Kramer HSD test was conducted for multiple comparisons of means between early and late maturity. Early maturity groups had significantly higher (p<0.05) root length and surface area of finer roots. Whereas the root average diameter was significantly higher (p<0.05) in late maturity groups compared to early maturity groups.


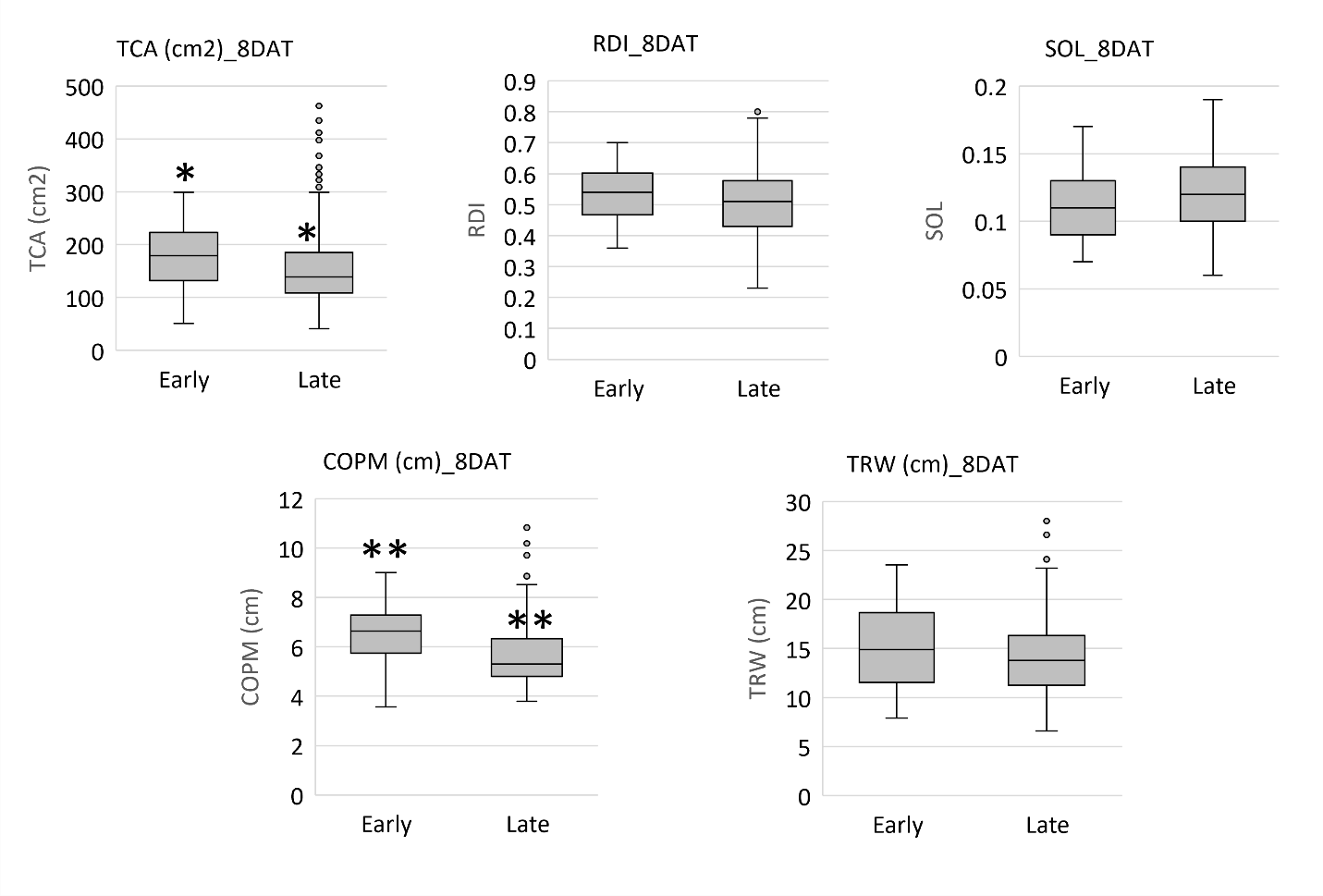


**Figure S8)** Variation in root architectural traits in early maturity (MG0, MGI and MGII) and late maturity groups (MGIII, MGIV, MGV and MGVI) of soybean accessions. The Tukey-Kramer HSD test was conducted for multiple comparisons of means between early and late maturity. The majority of the early maturity groups showed lower (statistically not significant p>0.05) root solidity and had a higher (statistically not significant p>0.05) root depth index (RDI) and total root width (TRW). Unit used for the center of mass trait (cm) is used as the trait represents the distance from the root system’s center of mass to the root:shoot junction. The center of mass was significantly (p<0.05) deeper, and the convex area was significantly (p<0.05) higher in early maturity groups compared to late maturity groups.


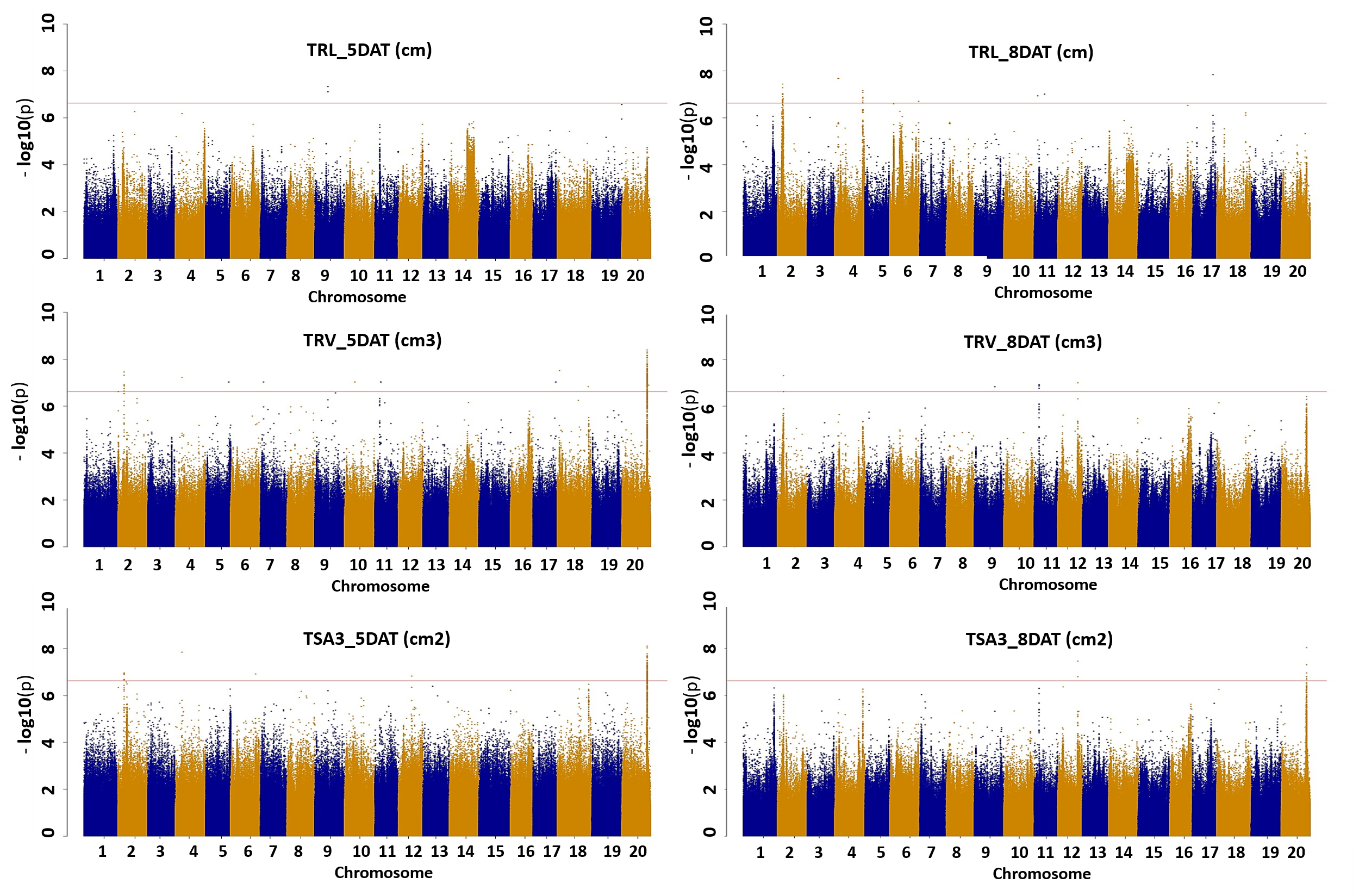


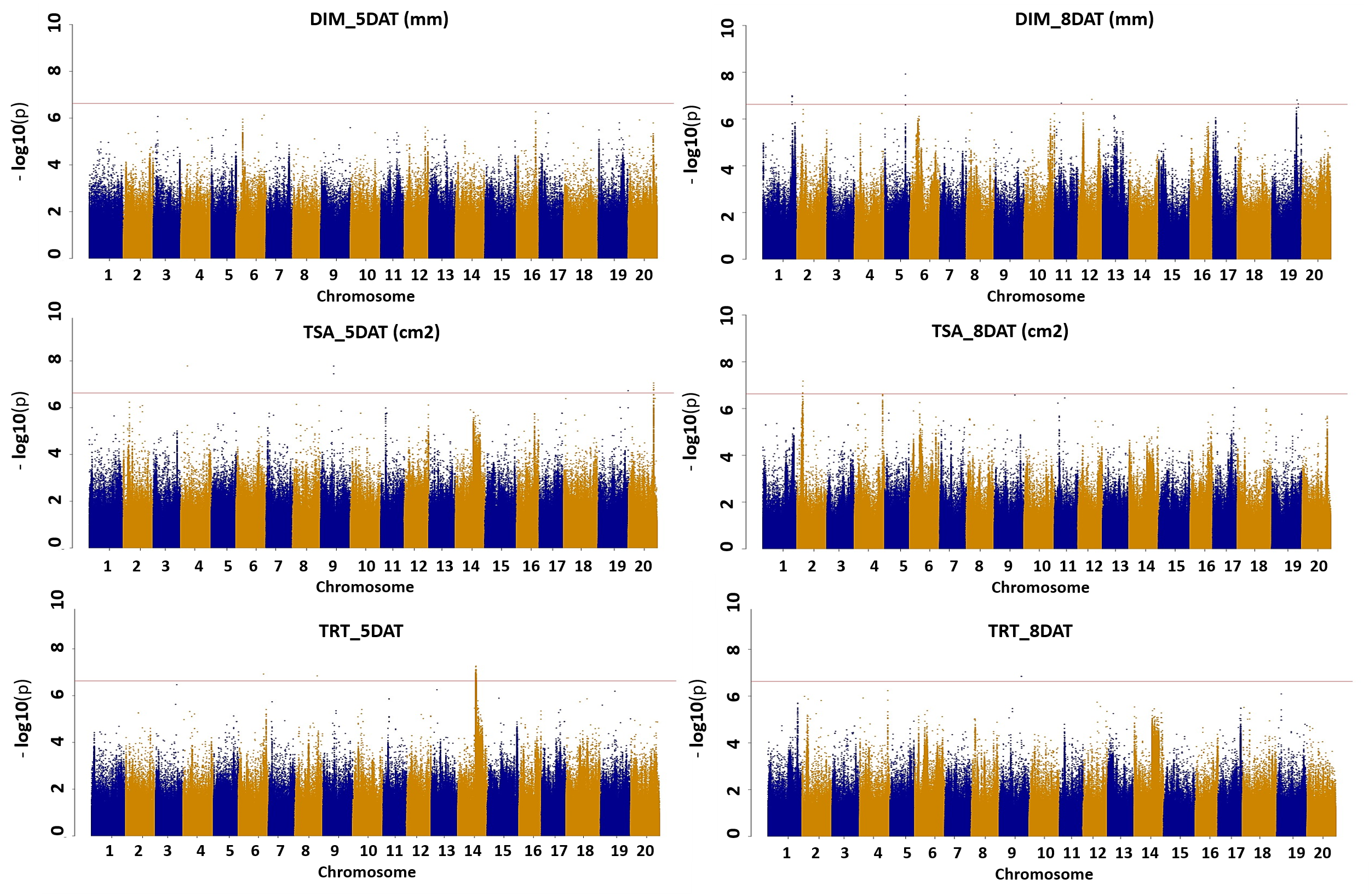


**
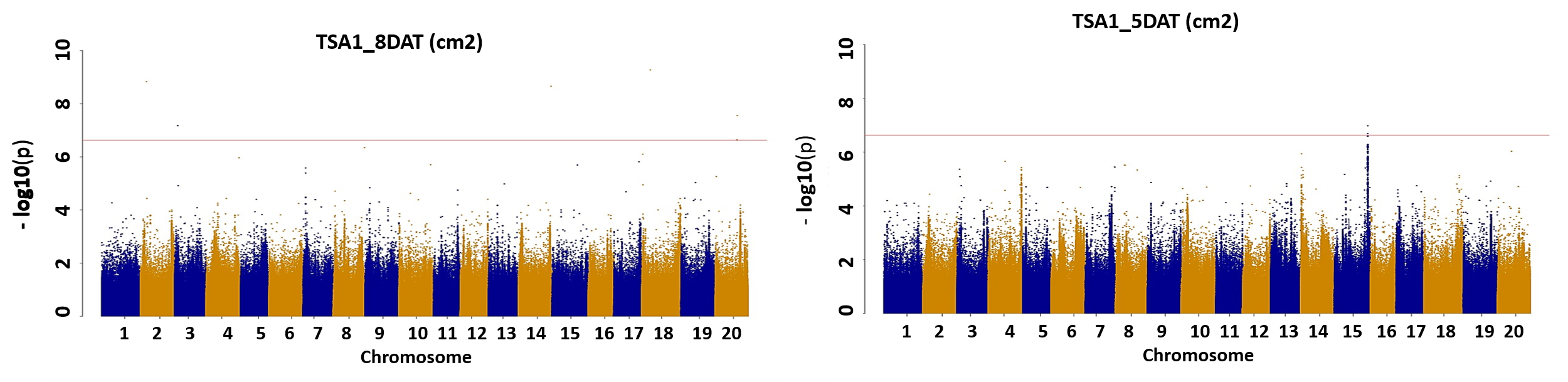
**

**Figure S9)** Manhattan plots for GWAS results from the FarmCPU model for all root morphological traits. The x‐axis denotes the -Log10 transformed p values across the 20 soybean chromosomes in a genome‐wide scan. The horizontal line indicates the genome‐wide significance threshold.

**
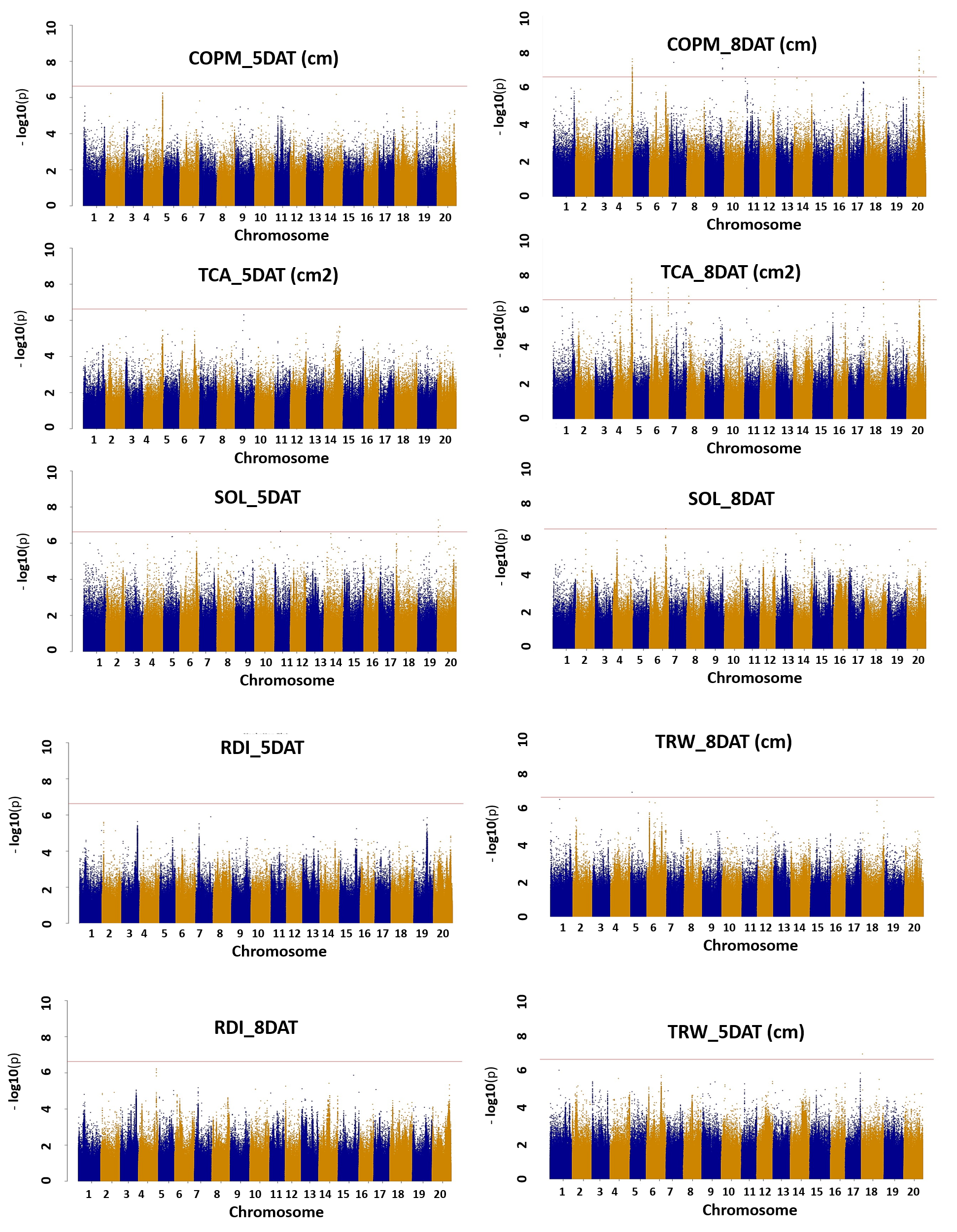
**

**
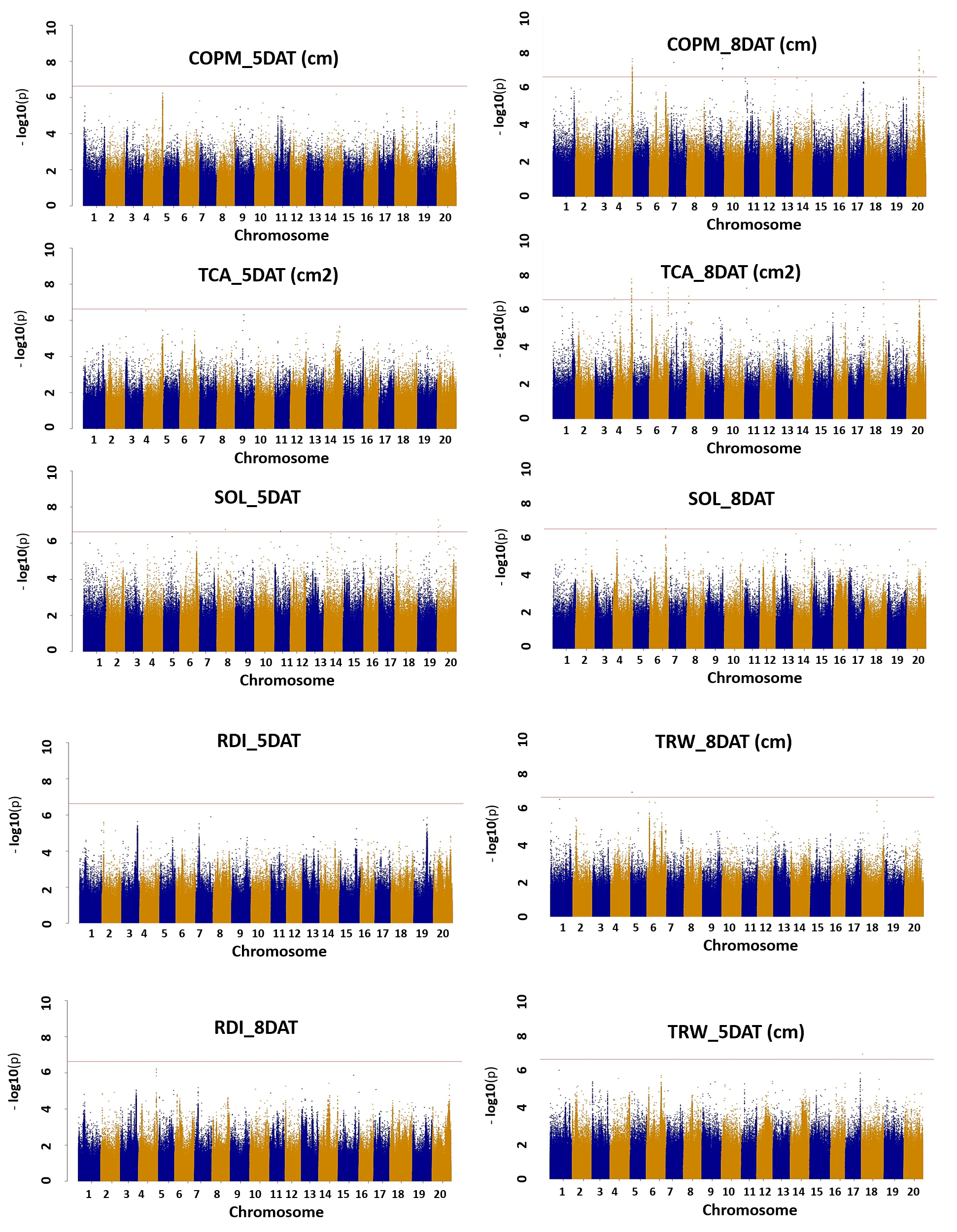
**

**Figure S10)** Manhattan plots for GWAS results from the FarmCPU model for all root architectural traits. The x‐axis denotes the -Log10 transformed p values across the 20 soybean chromosomes in a genome‐wide scan. The horizontal line indicates the genome‐wide significance threshold.

**
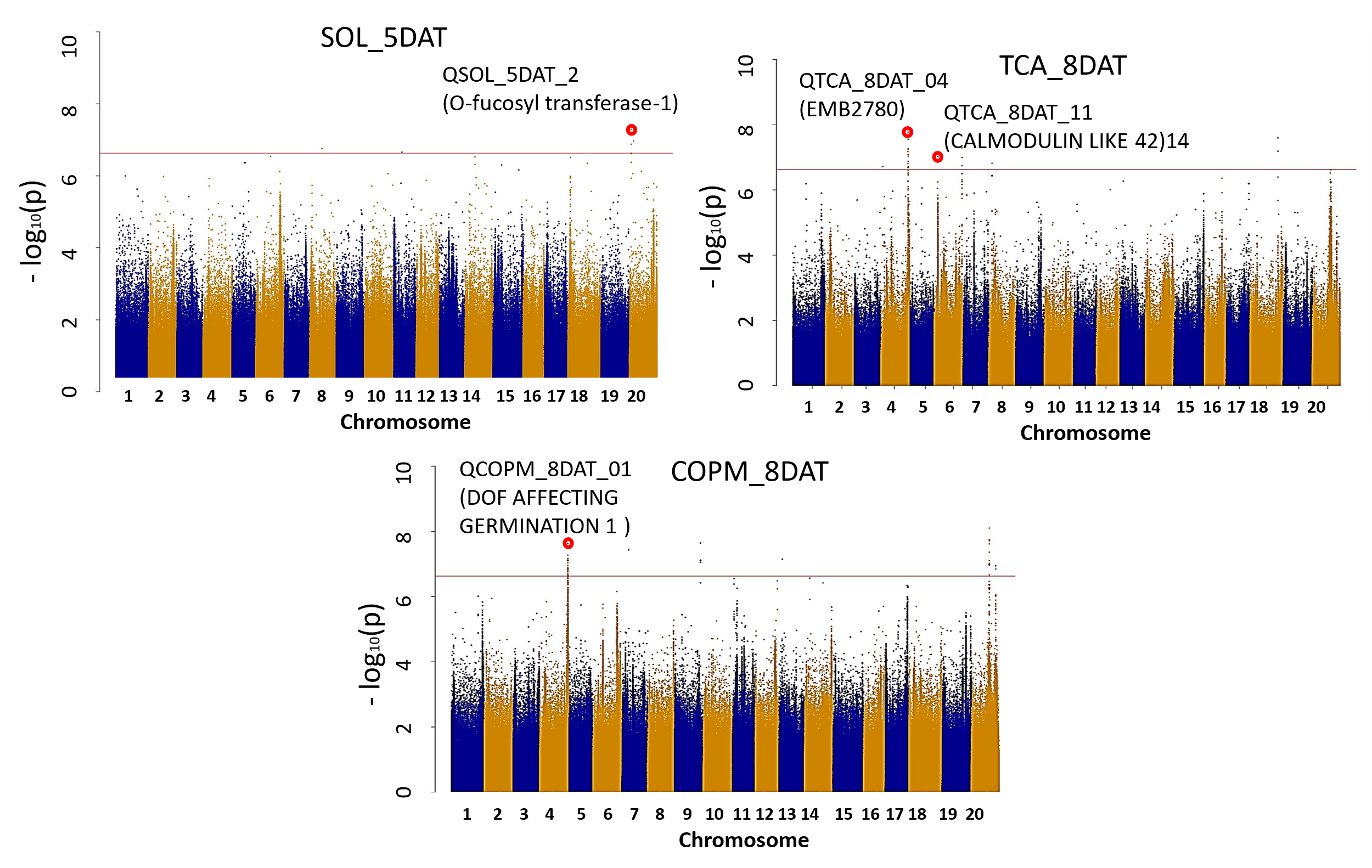
**

**Figure S11)** Manhattan plots exhibiting GWAS peaks and nearby putative candidate genes for a subset of architectural traits. Manhattan plots showing GWAS results by applying FarmCPU model for SNP- root. trait associations. For the p value threshold correction, the Bonferroni correction (43) was applied by treating each linkage block as one testing marker. The number of linkage blocks were calculated using Plink software scanning 200 kb windows for SNPs with a Pearson correlation coefficient of 0.2 or higher in the same linkage block. The threshold p value was calculated as -log (0.05/212612) = 6.6. Candidate genes are in close vicinity to significant GWAS peaks for the architectural traits: SOL (solidity) at 5DAT, TCA (total convex area) at 8DAT and COPM (center of mass) at 8DAT.

**
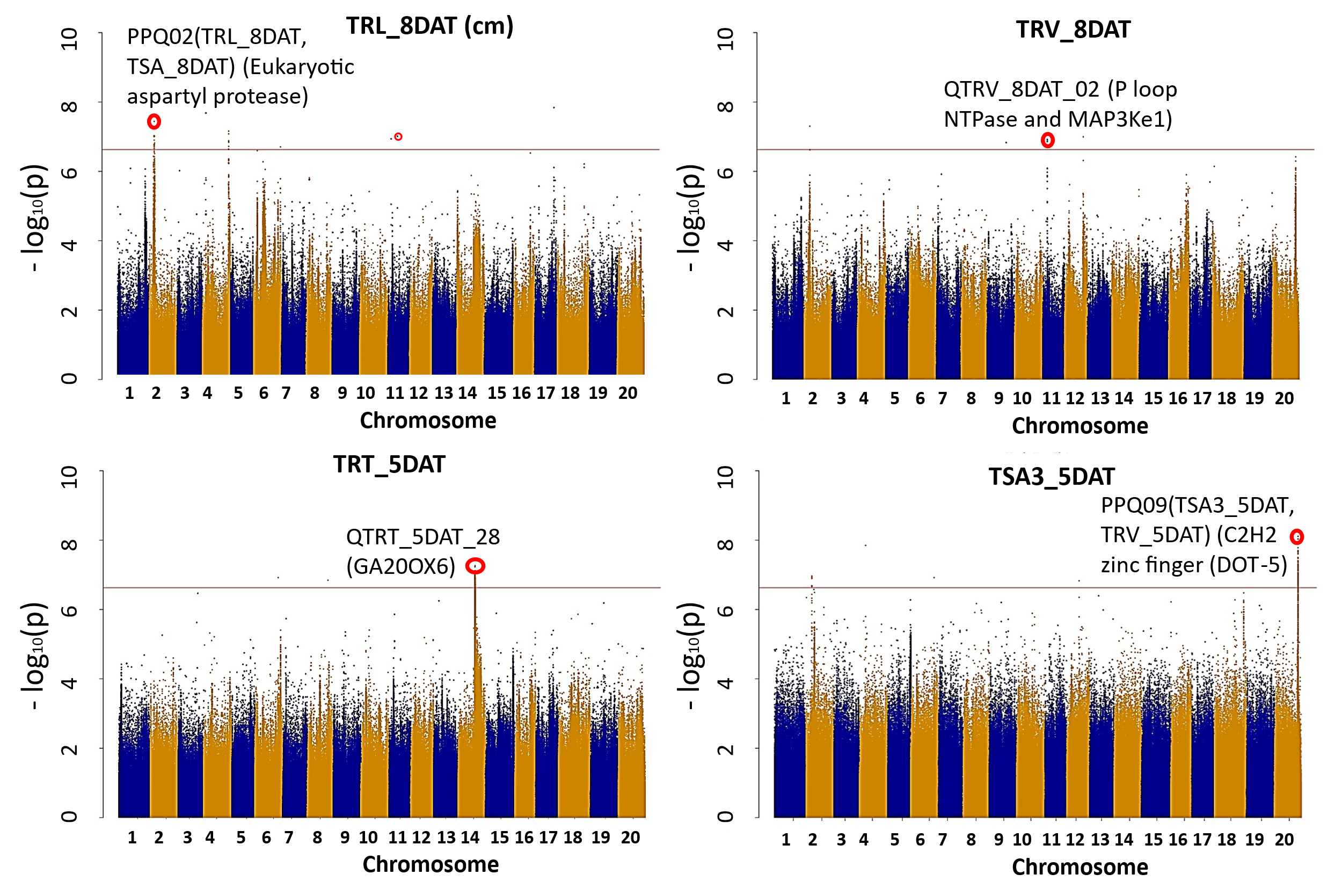
**

**Figure S12)** **Manhattan plots exhibiting GWAS peaks and nearby putative candidate genes for a subset of root morphological traits.** Manhattan plots and candidate genes in close vicinity to significant GWAS peaks for the root morphological traits: TRL (total root length) at 8DAT, TRV (total root volume) at 8DAT, TRT (total number of root tips) at 5DAT, and TSA3 (total surface area of diameter class 1.0 – 1.5 mm) at 5DAT.


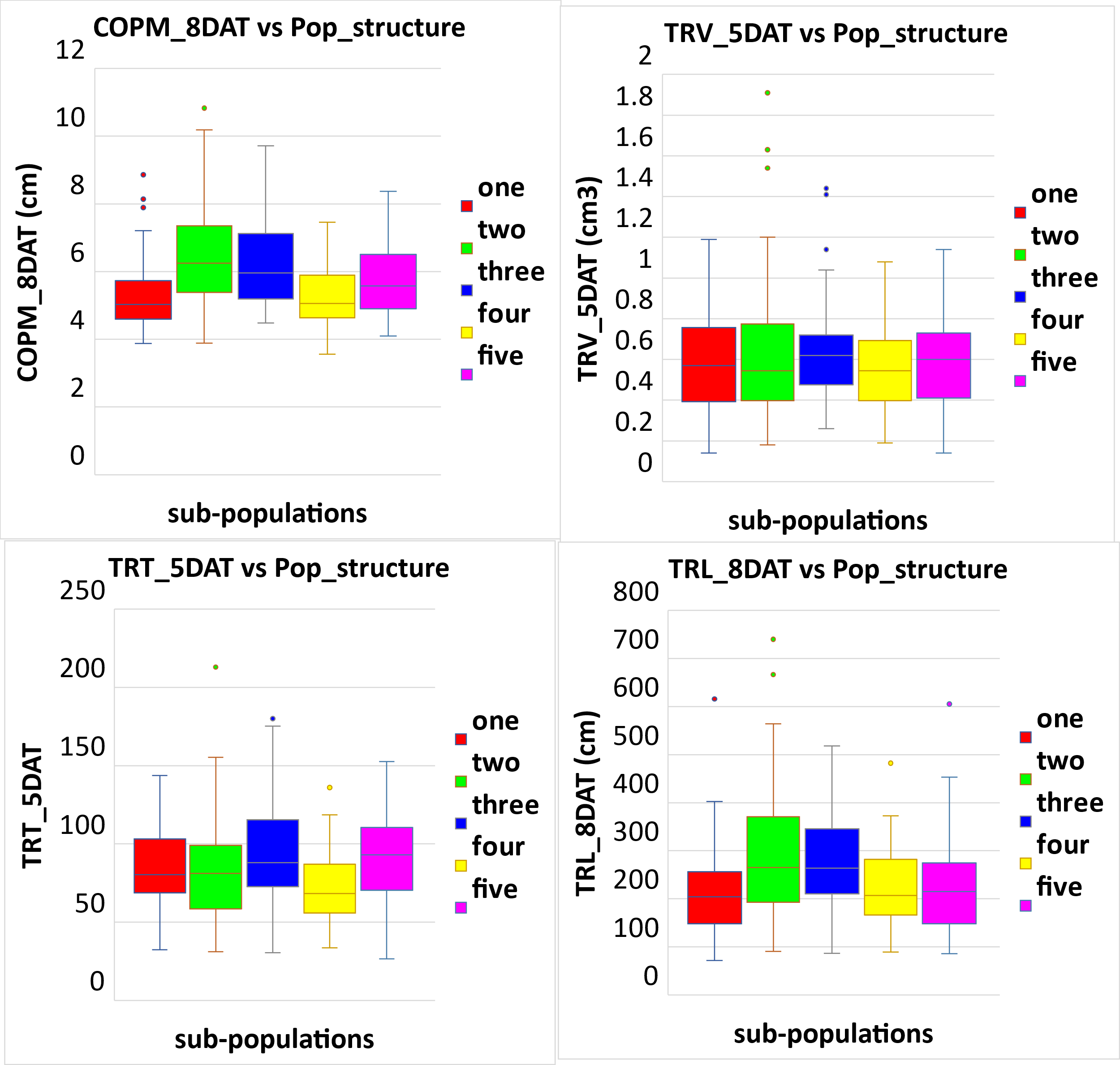


**Figure S13) Distribution of traits of interests among sub-populations based on structural analysis**. In the previous four figures, we identified four high priority candidate genes whose gene expression was variable among the soybean lines with contrasting alleles for the significantly associated SNP. Hence, we focused on the four traits related to those candidate genes to investigate the variation among sub-populations for those traits. Population structure was analyzed using STRUCTURE software (42) by selecting 12000 evenly spaced genome-wide SNPs. A burning period of 12000 was applied with 10 iterations. Four traits, COPM_8DAT, TRL_8DAT, TRV_5DAT and TRT_5DAT were evaluated for variation among sub-populations. We carried out the Tukey-Kramer HSD multiple comparison test and the different letters represent statistically significant (p<0.05) differences among sub-populations for all traits except TRV_5DAT.

**SUPPLEMENTARY TABLES**

**Table S1. Country of origin, maturity group, and key root trait values for all accessions in the soybean association panel**

| **Line/Accession** | **Country of origin** | **Maturity group** | **TRL (cm)_5DAT** | **TSA (cm2)_5DAT** | **DIM (mm)_5DAT** | **TRV (cm3)_5DAT** | **TRT_5DAT** | **TSA1_5DAT** |
| --- | --- | --- | --- | --- | --- | --- | --- | --- |
| **Lee** | **USA** | **VI** | **144.3** | **28.98** | **0.64** | **0.46** | **72.5** | **4.5** |
| **Magellan** | **USA** | **IV** | **130.1** | **29.66** | **0.73** | **0.54** | **79.8** | **3.6** |
| **Maverick** | **USA** | **III** | **118.8** | **31.34** | **0.84** | **0.66** | **65.47** | **1.55** |
| **PI091725** | **NK** | **V** | **174.6** | **40.5** | **0.74** | **0.75** | **106** | **3.06** |
| **PI398595** | **SK** | **V** | **148.9** | **28.47** | **0.61** | **0.44** | **66.6** | **6.16** |
| **PI408105A** | **SK** | **IV** | **281.3** | **59.76** | **0.68** | **1.01** | **131.8** | **11.5** |
| **PI417242** | **China** | **II** | **175.9** | **43.83** | **0.8** | **0.87** | **83.2** | **2.49** |
| **PI438500** | **USA** | **III** | **304.4** | **61.05** | **0.64** | **0.98** | **104.5** | **13.13** |
| **PI467347** | **China** | **II** | **141.6** | **33.28** | **0.76** | **0.64** | **117.7** | **2.59** |
| **PI518668** | **USA** | **IV** | **201.3** | **43.47** | **0.68** | **0.75** | **98.75** | **6.61** |
| **PI548316** | **China** | **III** | **200** | **46.79** | **0.74** | **0.87** | **126** | **6.05** |
| **PI561271** | **China** | **V** | **233.5** | **46.62** | **0.65** | **0.75** | **104.1** | **9.84** |
| **PI567305** | **China** | **IV** | **162.7** | **31.71** | **0.62** | **0.49** | **78.75** | **6.99** |
| **PI567343** | **China** | **V** | **186.2** | **37.75** | **0.65** | **0.61** | **129.8** | **8.15** |
| **PI567651** | **China** | **IV** | **172** | **39.45** | **0.74** | **0.73** | **82.6** | **4.54** |
| **PI092728** | **China** | **III** | **230.7** | **47.64** | **0.66** | **0.79** | **155.3** | **8.62** |
| **PI518671 (Williams 82)** | **USA** | **III** | **181.1** | **36.25** | **0.65** | **0.58** | **136** | **7.69** |
| **PI087617** | **NK** | **III** | **340.9** | **80.75** | **0.76** | **1.54** | **155.3** | **11.74** |
| **PI196175** | **SK** | **V** | **164.3** | **40.54** | **0.79** | **0.8** | **85.4** | **3.48** |
| **PI209332** | **Japan** | **IV** | **95.08** | **18.75** | **0.64** | **0.3** | **76.63** | **4.33** |
| **PI248515** | **Japan** | **IV** | **95.54** | **17.21** | **0.57** | **0.25** | **59.5** | **4.75** |
| **PI398593** | **SK** | **V** | **257.6** | **48.9** | **0.61** | **0.74** | **91.2** | **11.25** |
| **PI398610** | **SK** | **V** | **99.39** | **22.89** | **0.74** | **0.42** | **46.75** | **1.87** |
| **PI407788A** | **SK** | **IV** | **253.3** | **56.45** | **0.71** | **1** | **94.67** | **8.56** |
| **PI407965** | **SK** | **V** | **228.4** | **49.5** | **0.7** | **0.86** | **109** | **7.6** |
| **PI424608A** | **SK** | **IV** | **280.1** | **64.89** | **0.74** | **1.2** | **118.2** | **7.09** |
| **PI458515** | **China** | **IV** | **149** | **35.12** | **0.74** | **0.66** | **92.67** | **4.47** |
| **PI495017C** | **China** | **IV** | **90.09** | **20.26** | **0.72** | **0.36** | **52** | **2.59** |
| **PI603154** | **NK** | **V** | **135.3** | **36.09** | **0.86** | **0.77** | **72.75** | **1.92** |
| **PI603175** | **NK** | **IV** | **135** | **31.82** | **0.75** | **0.61** | **97.5** | **3.4** |
| **PI605869A** | **Vietnam** | **V** | **220.4** | **45.84** | **0.67** | **0.76** | **110.5** | **8.74** |
| **PI404166** | **China** | **III** | **212.7** | **42.64** | **0.64** | **0.69** | **111.3** | **6.68** |
| **PI417091** | **Japan** | **II** | **215.5** | **42.31** | **0.63** | **0.66** | **125** | **7.19** |
| **PI437169B** | **Russia** | **II** | **177.2** | **41.83** | **0.75** | **0.79** | **106.4** | **4.06** |
| **PI437655** | **China** | **III** | **191** | **45.02** | **0.75** | **0.85** | **88.4** | **2.43** |
| **PI437725** | **China** | **IV** | **189.8** | **36.96** | **0.62** | **0.57** | **113.5** | **7.1** |
| **PI468915** | **China** | **II** | **181.3** | **38.87** | **0.69** | **0.67** | **115.4** | **4.93** |
| **PI548359** | **China** | **IV** | **139.1** | **36.76** | **0.85** | **0.78** | **77.8** | **1.66** |
| **PI548402 (peking)** | **China** | **IV** | **133.5** | **30.19** | **0.72** | **0.54** | **88.86** | **3.79** |
| **PI548427** | **China** | **IV** | **172.2** | **45.03** | **0.83** | **0.94** | **87.5** | **2.05** |
| **PI548619** | **USA** | **IV** | **170.1** | **39.77** | **0.75** | **0.74** | **101** | **2.63** |
| **PI548633** | **Japan** | **IV** | **91.84** | **23.06** | **0.8** | **0.46** | **78** | **1.61** |
| **PI548696** | **USA** | **V** | **227** | **47.21** | **0.67** | **0.78** | **130.5** | **6.78** |
| **PI556511** | **Japan** | **III** | **96.89** | **24.88** | **0.85** | **0.52** | **71.5** | **1.68** |
| **PI567387** | **China** | **IV** | **174.9** | **38.44** | **0.71** | **0.68** | **116** | **4.08** |
| **PI437487** | **Russia** | **III** | **69.86** | **15.13** | **0.68** | **0.26** | **46** | **2.12** |
| **PI533654** | **USA** | **IV** | **118.1** | **21.85** | **0.59** | **0.32** | **65.6** | **6.48** |
| **PI539936** | **USA** | **IV** | **150.2** | **28.82** | **0.61** | **0.44** | **109.5** | **6.69** |
| **PI540555** | **USA** | **IV** | **156.4** | **34.03** | **0.69** | **0.59** | **99** | **4.59** |
| **PI548158** | **USA** | **IV** | **67.79** | **12.41** | **0.59** | **0.18** | **42.4** | **3.89** |
| **PI548200** | **USA** | **IV** | **173.6** | **40.23** | **0.74** | **0.75** | **124.6** | **3.77** |
| **PI561701** | **USA** | **VI** | **114.1** | **21.36** | **0.6** | **0.32** | **71.5** | **6.18** |
| **PI378663** | **Russia** | **I** | **162.2** | **36.31** | **0.7** | **0.65** | **115.7** | **4.4** |
| **PI417550** | **Russia** | **0** | **154.1** | **31.96** | **0.66** | **0.53** | **84.67** | **5.45** |
| **PI437123** | **Russia** | **I** | **114.6** | **24.98** | **0.7** | **0.43** | **64** | **2.58** |
| **PI437138** | **Russia** | **0** | **157.2** | **34.98** | **0.71** | **0.62** | **83.8** | **4.3** |
| **PI437366** | **Russia** | **I** | **173.5** | **30.47** | **0.57** | **0.43** | **84** | **9.86** |
| **PI437476** | **Russia** | **III** | **123.6** | **24.95** | **0.65** | **0.41** | **69.5** | **5.71** |
| **PI507678** | **Russia** | **I** | **108.3** | **21.61** | **0.63** | **0.34** | **56.5** | **4.75** |
| **PI548169** | **USA** | **IV** | **239.1** | **43.49** | **0.58** | **0.63** | **123** | **10.43** |
| **PI548178** | **USA** | **III** | **100.3** | **22.42** | **0.71** | **0.4** | **57** | **2.72** |
| **PI548313** | **Russia** | **III** | **102.7** | **21.17** | **0.65** | **0.35** | **67.25** | **3.45** |
| **PI548325** | **Russia** | **0** | **179.2** | **37.77** | **0.67** | **0.64** | **114.5** | **5.6** |
| **PI548336** | **Russia** | **I** | **178.5** | **31.54** | **0.57** | **0.44** | **91.75** | **8.99** |
| **PI556637** | **USA** | **I** | **239.8** | **41.66** | **0.57** | **0.58** | **114.8** | **13.97** |
| **PI567226** | **Russia** | **0** | **126** | **27.64** | **0.71** | **0.49** | **74.33** | **3.59** |
| **PI597402** | **Russia** | **0** | **146** | **29.64** | **0.66** | **0.48** | **86** | **6.5** |
| **PI062202-2** | **China** | **IV** | **233.4** | **51.76** | **0.71** | **0.92** | **109.5** | **4.85** |
| **PI063945** | **China** | **IV** | **123** | **25.66** | **0.68** | **0.43** | **97** | **3.7** |
| **PI079616** | **China** | **III** | **106.7** | **27.44** | **0.82** | **0.56** | **73.6** | **1.25** |
| **PI084509** | **Unknown** | **III** | **81.44** | **19.1** | **0.77** | **0.37** | **99.5** | **1.88** |
| **PI088306** | **China** | **III** | **92.4** | **22.03** | **0.77** | **0.42** | **57** | **1.13** |
| **PI090723** | **Unknown** | **III** | **123.5** | **25.48** | **0.66** | **0.42** | **79** | **4.01** |
| **PI091731-1** | **China** | **IV** | **144.4** | **35.87** | **0.8** | **0.72** | **85.4** | **2.61** |
| **PI504495** | **Taiwan** | **V** | **114.8** | **24.78** | **0.69** | **0.43** | **100** | **2.96** |
| **PI593258** | **USA** | **III** | **133.6** | **32.98** | **0.79** | **0.65** | **84** | **2.4** |
| **PI525454** | **USA** | **IV** | **137.9** | **34.39** | **0.8** | **0.69** | **65** | **1.42** |
| **PI548193** | **USA** | **IV** | **116.8** | **31.11** | **0.84** | **0.67** | **56.5** | **1.79** |
| **PI548511** | **USA** | **II** | **89.43** | **24.18** | **0.87** | **0.52** | **64.2** | **0.53** |
| **PI548547** | **USA** | **IV** | **139.1** | **35.2** | **0.84** | **0.72** | **74.5** | **1.29** |
| **PI559932** | **USA** | **IV** | **97.28** | **20.87** | **0.71** | **0.36** | **69.75** | **2.71** |
| **PI597384** | **USA** | **IV** | **67.19** | **17.14** | **0.82** | **0.35** | **34.75** | **0.34** |
| **PI060970** | **China** | **IV** | **130.1** | **34.87** | **0.82** | **0.75** | **78** | **1.66** |
| **PI070242-2** | **China** | **IV** | **261.6** | **68.67** | **0.84** | **1.44** | **175.3** | **3.27** |
| **PI079797** | **China** | **III** | **209.2** | **43.07** | **0.66** | **0.71** | **137** | **7.94** |
| **PI088448** | **China** | **III** | **197.4** | **40.52** | **0.66** | **0.67** | **146** | **8.43** |
| **PI090369** | **China** | **IV** | **144.6** | **35.33** | **0.8** | **0.69** | **116.4** | **2.8** |
| **PI180501** | **Germany** | **0** | **174.7** | **37.1** | **0.68** | **0.63** | **122.8** | **6.37** |
| **PI360957** | **Japan** | **0** | **118.7** | **28.61** | **0.75** | **0.55** | **81** | **2.77** |
| **PI404161** | **Georgia** | **IV** | **100.3** | **23.5** | **0.74** | **0.44** | **87.75** | **2.85** |
| **PI416751** | **Japan** | **I** | **164.8** | **40.51** | **0.77** | **0.79** | **98.75** | **2.21** |
| **PI417529** | **Germany** | **0** | **111.2** | **25.25** | **0.72** | **0.46** | **54** | **1.35** |
| **PI438312** | **Algeria** | **III** | **210.9** | **43.37** | **0.66** | **0.71** | **152.7** | **8.33** |
| **PI518751** | **Former Serbia and Montenegro** | **II** | **192.3** | **45.34** | **0.76** | **0.86** | **118.1** | **4.02** |
| **PI548414** | **Japan** | **0** | **186.9** | **43.02** | **0.74** | **0.79** | **111** | **4.41** |
| **PI068521_1** | **China** | **II** | **115.8** | **27.02** | **0.73** | **0.5** | **69.5** | **2.06** |
| **PI068679-2** | **China** | **IV** | **150** | **36.82** | **0.78** | **0.72** | **128.6** | **2.17** |
| **PI081042-2** | **Japan** | **IV** | **162.5** | **38.74** | **0.75** | **0.74** | **114** | **2.3** |
| **PI089772** | **China** | **IV** | **157.3** | **37.56** | **0.76** | **0.71** | **106.4** | **3.2** |
| **PI153281** | **Belgium** | **0** | **192.1** | **38.8** | **0.64** | **0.62** | **120.8** | **7.18** |
| **PI209331** | **Japan** | **III** | **96.08** | **24** | **0.8** | **0.48** | **101.5** | **1.08** |
| **PI290136** | **France** | **0** | **127.2** | **31.9** | **0.8** | **0.65** | **74.33** | **1.79** |
| **PI438323** | **France** | **I** | **197** | **45.25** | **0.73** | **0.83** | **121.3** | **3.32** |
| **PI438335** | **Algeria** | **III** | **186.2** | **38.76** | **0.66** | **0.64** | **78.4** | **6.04** |
| **PI507467** | **Japan** | **IV** | **166.6** | **55.61** | **1.08** | **1.63** | **102** | **2.59** |
| **PI087571** | **NK** | **IV** | **106.4** | **27.55** | **0.82** | **0.57** | **78** | **1.49** |
| **PI087618** | **NK** | **III** | **174.6** | **42.45** | **0.77** | **0.82** | **98** | **2.58** |
| **PI154189** | **Netherlands** | **0** | **88.91** | **21.94** | **0.78** | **0.43** | **71.67** | **1.13** |
| **PI198067** | **Sweden** | **0** | **172** | **38.31** | **0.71** | **0.68** | **109.2** | **3.71** |
| **PI361093** | **Serbia** | **I** | **102.3** | **26.95** | **0.84** | **0.57** | **92** | **0.95** |
| **PI372418** | **Serbia** | **I** | **142.2** | **32.97** | **0.74** | **0.61** | **83** | **2.44** |
| **PI398614** | **SK** | **V** | **83.61** | **17.56** | **0.68** | **0.3** | **54.63** | **2.68** |
| **PI398633** | **SK** | **V** | **138.8** | **27.92** | **0.64** | **0.45** | **85.13** | **5.49** |
| **PI398965** | **SK** | **IV** | **155** | **31.78** | **0.65** | **0.52** | **93.86** | **6.79** |
| **PI408088** | **SK** | **V** | **143.5** | **32.04** | **0.71** | **0.57** | **80.5** | **2.97** |
| **PI424005** | **SK** | **III** | **104.1** | **20.25** | **0.62** | **0.32** | **69.89** | **3.65** |
| **PI430595** | **China** | **IV** | **230.1** | **49.21** | **0.68** | **0.84** | **133.8** | **7.6** |
| **PI437287** | **Moldova** | **0** | **118.2** | **41.98** | **1.13** | **1.41** | **68** | **0.6** |
| **PI437296** | **Moldova** | **0** | **99.16** | **23.55** | **0.75** | **0.45** | **86** | **1.55** |
| **PI567225** | **Moldova** | **0** | **124.8** | **31.06** | **0.79** | **0.62** | **71.5** | **1.69** |
| **PI597476** | **SK** | **V** | **125.7** | **24.8** | **0.63** | **0.39** | **74.25** | **4.86** |
| **PI612611** | **NK** | **III** | **239.7** | **58.59** | **0.78** | **1.14** | **102** | **2.33** |
| **Holladay (S-100)** | **USA** | **V** | **91.96** | **24.54** | **0.85** | **0.52** | **60.6** | **1.04** |
| **IA3023** | **USA** | **III** | **100.7** | **26.32** | **0.83** | **0.55** | **58.44** | **1.58** |
| **PI088788** | **China** | **III** | **84.94** | **20.8** | **0.77** | **0.41** | **57.2** | **2.06** |
| **PI090763** | **China** | **IV** | **71.99** | **17.12** | **0.76** | **0.32** | **47** | **1.91** |
| **PI404198B** | **China** | **IV** | **83.54** | **18.84** | **0.72** | **0.34** | **58.2** | **2.41** |
| **PI407729** | **China** | **IV** | **121.2** | **28.49** | **0.75** | **0.53** | **74.4** | **3.12** |
| **PI437654** | **China** | **III** | **120.3** | **28.83** | **0.77** | **0.55** | **76.63** | **3.02** |
| **PI437679** | **China** | **IV** | **110.5** | **30.05** | **0.87** | **0.65** | **61.6** | **1.32** |
| **PI437690** | **China** | **III** | **77.25** | **16.47** | **0.68** | **0.28** | **57.8** | **3.14** |
| **PI438258** | **China** | **II** | **128.1** | **35.72** | **0.89** | **0.79** | **67.6** | **1.31** |
| **PI467312** | **China** | **II** | **85.66** | **20.9** | **0.78** | **0.41** | **52.6** | **1.53** |
| **PI475783B** | **China** | **III** | **139.8** | **37.62** | **0.85** | **0.81** | **74.8** | **1.96** |
| **PI547862** | **USA** | **III** | **91.23** | **26.35** | **0.9** | **0.62** | **58** | **1.03** |
| **PI548317** | **China** | **III** | **78.04** | **19.03** | **0.78** | **0.37** | **49.8** | **1.58** |
| **PI548415** | **China** | **IV** | **84.99** | **19.82** | **0.74** | **0.37** | **52.8** | **1.65** |
| **PI567516C** | **China** | **IV** | **79.25** | **17.17** | **0.69** | **0.3** | **53** | **2.7** |
| **FC033243** | **Algeria** | **IV** | **73.75** | **17.6** | **0.76** | **0.34** | **51** | **1.89** |
| **PI549018** | **China** | **V** | **63.79** | **13.24** | **0.66** | **0.22** | **46.5** | **2.74** |
| **PI549031** | **China** | **III** | **104** | **25.42** | **0.79** | **0.5** | **66.2** | **2.48** |
| **PI552538** | **USA** | **III** | **124.2** | **34.4** | **0.86** | **0.76** | **68.4** | **1.16** |
| **PI567230** | **China** | **V** | **64.54** | **13.55** | **0.67** | **0.23** | **42.2** | **2.57** |
| **PI567336B** | **China** | **IV** | **87.82** | **18.83** | **0.68** | **0.32** | **64** | **3.18** |
| **PI567354** | **China** | **IV** | **147.3** | **37.95** | **0.84** | **0.78** | **92.4** | **3.65** |
| **PI567357** | **China** | **III** | **191.9** | **53.59** | **0.89** | **1.19** | **112.8** | **3.09** |
| **PI567383** | **China** | **V** | **124.8** | **33.21** | **0.84** | **0.71** | **75.8** | **2.37** |
| **PI567519** | **China** | **III** | **85.41** | **22.61** | **0.85** | **0.48** | **53.2** | **1.91** |
| **PI567611** | **China** | **IV** | **73.05** | **17.91** | **0.8** | **0.35** | **55** | **2.63** |
| **PI567690** | **China** | **III** | **64.33** | **17.14** | **0.86** | **0.37** | **47.67** | **1.25** |
| **PI567731** | **China** | **III** | **67.6** | **17.4** | **0.82** | **0.36** | **44.6** | **1.21** |
| **PI594599** | **China** | **IV** | **139.6** | **33.17** | **0.76** | **0.63** | **92.8** | **4.42** |
| **PI597387** | **USA** | **III** | **97.52** | **26.19** | **0.85** | **0.56** | **58.5** | **1.72** |
| **PI603176A** | **NK** | **IV** | **107.7** | **26.18** | **0.77** | **0.51** | **54.5** | **1.31** |
| **PI639740 (LD00-3309)** | **USA** | **IV** | **117.6** | **28.43** | **0.77** | **0.55** | **74.11** | **2.77** |
| **PI092651** | **China** | **IV** | **137.5** | **35.99** | **0.83** | **0.75** | **88** | **2.57** |
| **PI103088** | **China** | **III** | **183.6** | **48.5** | **0.83** | **1.02** | **100.2** | **4.25** |
| **PI153231** | **China** | **III** | **161.4** | **45.27** | **0.89** | **1.01** | **95** | **2.89** |
| **PI171428** | **China** | **IV** | **151.8** | **38.15** | **0.79** | **0.77** | **105.2** | **3.99** |
| **PI253661B** | **China** | **III** | **165.4** | **36.33** | **0.7** | **0.64** | **117** | **6.2** |
| **PI379618** | **China** | **V** | **149.6** | **37.5** | **0.8** | **0.75** | **82** | **3.44** |
| **PI407742** | **China** | **V** | **145.7** | **34.79** | **0.77** | **0.67** | **115** | **3.33** |
| **PI417581** | **China** | **V** | **123.3** | **30.4** | **0.78** | **0.6** | **80.67** | **1.56** |
| **PI424038B** | **China** | **V** | **83.69** | **16.09** | **0.61** | **0.25** | **58.2** | **3.54** |
| **PI437110A** | **China** | **III** | **117.2** | **29.69** | **0.81** | **0.6** | **80.25** | **2.66** |
| **PI437127A** | **China** | **IV** | **213.5** | **47.38** | **0.7** | **0.84** | **139.8** | **8.79** |
| **PI437685D** | **China** | **III** | **123.3** | **31.28** | **0.8** | **0.63** | **74.2** | **2.91** |
| **PI437776** | **China** | **III** | **222.6** | **53.57** | **0.76** | **1.04** | **142.3** | **8.99** |
| **FC029333** | **China** | **III** | **152.8** | **40.33** | **0.82** | **0.86** | **101** | **3.5** |
| **FC031697** | **China** | **IV** | **116.9** | **31.22** | **0.83** | **0.67** | **77.67** | **1.52** |
| **PI054615_1** | **China** | **III** | **155.3** | **37.79** | **0.78** | **0.74** | **98.6** | **4.6** |
| **PI058955** | **China** | **IV** | **96.01** | **23.6** | **0.78** | **0.46** | **66.6** | **1.72** |
| **PI068604_1** | **China** | **III** | **128.5** | **29.47** | **0.72** | **0.54** | **91.88** | **4.76** |
| **PI070466_3** | **China** | **IV** | **158.3** | **38.08** | **0.77** | **0.73** | **112.5** | **4.33** |
| **PI071465** | **China** | **V** | **205.3** | **47.09** | **0.73** | **0.86** | **93.2** | **6.58** |
| **PI083925** | **SK** | **IV** | **181.9** | **41.02** | **0.72** | **0.74** | **109.5** | **5.16** |
| **PI084946_2** | **China** | **IV** | **334** | **89.47** | **0.86** | **1.91** | **213** | **9.04** |
| **PI086972_2** | **China** | **IV** | **268** | **61.99** | **0.73** | **1.14** | **180** | **7.77** |
| **PI090479P** | **China** | **IV** | **117** | **30.76** | **0.86** | **0.65** | **60.25** | **2.78** |
| **PI090486** | **China** | **III** | **124.5** | **30.04** | **0.76** | **0.58** | **96.4** | **3.42** |
| **PI567426** | **China** | **IV** | **179.1** | **41.21** | **0.73** | **0.75** | **111.3** | **5.85** |
| **PI567558** | **China** | **III** | **218.3** | **49.2** | **0.72** | **0.88** | **134.5** | **6.37** |
| **PI578495** | **China** | **IV** | **168.3** | **40.21** | **0.76** | **0.77** | **108.8** | **4.9** |
| **PI603442** | **China** | **III** | **157.4** | **39.05** | **0.79** | **0.77** | **96.75** | **3.38** |
| **PI468408B** | **China** | **III** | **118.7** | **28.37** | **0.76** | **0.54** | **86.75** | **2.38** |
| **PI479735** | **France** | **III** | **113.3** | **29.52** | **0.83** | **0.61** | **95.5** | **1.58** |
| **PI490766** | **Georgia** | **III** | **158.6** | **42.67** | **0.86** | **0.92** | **119.3** | **1.93** |
| **PI495020** | **Germany** | **IV** | **66.45** | **15.07** | **0.72** | **0.27** | **43.25** | **1.55** |
| **PI561371** | **Moldova** | **IV** | **105.6** | **26.38** | **0.8** | **0.53** | **80.67** | **1.86** |
| **PI567307** | **NK** | **IV** | **105.8** | **21.88** | **0.66** | **0.36** | **109.5** | **4.09** |
| **PI567415A** | **Russia** | **IV** | **98.24** | **25.8** | **0.84** | **0.54** | **71.67** | **1.96** |
| **PI567416** | **Russia** | **IV** | **123** | **27.74** | **0.72** | **0.5** | **94.8** | **3.49** |
| **PI567428** | **Russia** | **IV** | **103.6** | **22.97** | **0.71** | **0.41** | **93** | **3.24** |
| **PI567435B** | **Russia** | **III** | **130** | **31.21** | **0.76** | **0.61** | **96** | **3.4** |
| **PI567439** | **Russia** | **V** | **117.8** | **30.19** | **0.82** | **0.62** | **102.2** | **1.75** |
| **PI567488A** | **Russia** | **IV** | **147.7** | **37.09** | **0.8** | **0.74** | **106** | **3.28** |
| **PI567532** | **Russia** | **IV** | **105.7** | **27.23** | **0.82** | **0.56** | **70.2** | **1.83** |
| **PI567548** | **Russia** | **IV** | **99.64** | **21.44** | **0.69** | **0.37** | **81.33** | **3.58** |
| **PI567576** | **Russia** | **III** | **165** | **41.68** | **0.8** | **0.84** | **129.8** | **4.29** |
| **PI567780B** | **China** | **IV** | **110.8** | **24.08** | **0.69** | **0.42** | **86.4** | **3.08** |
| **PI538386A** | **Japan** | **III** | **194.3** | **37.52** | **0.62** | **0.58** | **122.3** | **9.97** |
| **PI548383** | **Japan** | **III** | **115.4** | **26.48** | **0.74** | **0.48** | **96** | **3.22** |
| **PI548400** | **Japan** | **IV** | **140.7** | **33.45** | **0.75** | **0.64** | **88.75** | **2.31** |
| **PI567346** | **NK** | **V** | **99.04** | **22.18** | **0.72** | **0.4** | **67.25** | **2.12** |
| **PI567352A** | **Peru** | **IV** | **179.7** | **45.49** | **0.81** | **0.92** | **102** | **5** |
| **PI567353** | **Philippines** | **IV** | **163.9** | **36.04** | **0.7** | **0.63** | **109** | **4.88** |
| **PI567361** | **Philippines** | **III** | **243.3** | **49.5** | **0.65** | **0.81** | **131.3** | **11.08** |
| **PI567407** | **Romania** | **V** | **222.3** | **47.03** | **0.68** | **0.8** | **135** | **9.56** |
| **PI567408** | **Russia** | **V** | **136.3** | **33.18** | **0.77** | **0.65** | **78.4** | **2.79** |
| **PI567675** | **Russia** | **IV** | **118** | **26.16** | **0.73** | **0.48** | **79.4** | **4.29** |
| **PI567685** | **Russia** | **IV** | **98.58** | **22.18** | **0.72** | **0.4** | **55.5** | **2.57** |
| **PI567698A** | **Russia** | **IV** | **131.7** | **30.28** | **0.75** | **0.56** | **80.6** | **4.08** |
| **PI567726** | **Russia** | **IV** | **171.3** | **34.53** | **0.64** | **0.56** | **98** | **7.71** |
| **PI567746** | **Russia** | **IV** | **93.11** | **20.21** | **0.69** | **0.35** | **68** | **3.33** |
| **PI574477** | **SK** | **IV** | **168.6** | **41.92** | **0.78** | **0.83** | **118.6** | **3.86** |
| **PI587588B** | **SK** | **V** | **157.8** | **32.29** | **0.65** | **0.53** | **98.25** | **6.54** |
| **PI587712B** | **SK** | **V** | **126.2** | **28.03** | **0.7** | **0.5** | **89.2** | **3.65** |
| **PI068423** | **China** | **III** | **98.04** | **22.01** | **0.72** | **0.39** | **65** | **3.02** |
| **PI068523** | **China** | **III** | **114.4** | **30.11** | **0.82** | **0.63** | **89.5** | **2.06** |
| **PI068732_1** | **China** | **II** | **118.4** | **26.99** | **0.73** | **0.49** | **80** | **2.7** |
| **PI297505** | **China** | **I** | **82.12** | **17.75** | **0.69** | **0.31** | **71** | **2.53** |
| **PI417381** | **Japan** | **0** | **100.4** | **23.19** | **0.74** | **0.43** | **85.5** | **2.62** |
| **PI603495B** | **China** | **V** | **116.3** | **29.9** | **0.8** | **0.62** | **68.5** | **1.9** |
| **PI603526** | **China** | **IV** | **148.7** | **35.58** | **0.77** | **0.68** | **94** | **2.94** |
| **PI603549** | **China** | **III** | **158.6** | **37.2** | **0.74** | **0.7** | **121.2** | **4.2** |
| **PI603556** | **China** | **III** | **113.3** | **27.62** | **0.77** | **0.54** | **47** | **1.81** |
| **PI070208** | **China** | **IV** | **115.6** | **26.61** | **0.73** | **0.49** | **68.4** | **2.27** |
| **PI079870-4** | **China** | **IV** | **55.71** | **13.62** | **0.77** | **0.27** | **30.4** | **0.87** |
| **PI430598B** | **China** | **IV** | **134.4** | **33.83** | **0.8** | **0.68** | **76** | **2.83** |
| **PI594456A** | **China** | **III** | **93.79** | **18.05** | **0.61** | **0.28** | **69.6** | **4.31** |
| **PI594880** | **China** | **V** | **76.45** | **17.64** | **0.74** | **0.32** | **43** | **1.35** |
| **PI594922** | **USA** | **V** | **154.9** | **39.01** | **0.8** | **0.79** | **79.33** | **2.3** |
| **PI597478B** | **SK** | **III** | **94.26** | **22.37** | **0.75** | **0.42** | **38** | **0.87** |
| **PI598358** | **Unknown** | **V** | **82.32** | **17.51** | **0.68** | **0.3** | **54.2** | **2.74** |
| **PI606374** | **Vietnam** | **III** | **47.24** | **9.1** | **0.62** | **0.14** | **32.33** | **1.91** |
| **PI632418** | **USA** | **V** | **81.24** | **20.03** | **0.78** | **0.39** | **52.75** | **1.19** |
| **PI093055S** | **China** | **V** | **101.3** | **22.94** | **0.75** | **0.42** | **71.75** | **2.58** |
| **PI093563** | **China** | **III** | **177.1** | **39.86** | **0.71** | **0.72** | **84.5** | **5.98** |
| **PI103079** | **China** | **V** | **120.3** | **25.29** | **0.66** | **0.42** | **79.25** | **4.67** |
| **PI123587** | **Belgium** | **V** | **146.5** | **29.42** | **0.72** | **0.49** | **91.5** | **7.08** |
| **PI235347** | **Japan** | **IV** | **226.4** | **40.76** | **0.59** | **0.59** | **153** | **13.16** |
| **PI253656B** | **China** | **IV** | **124.6** | **27.6** | **0.72** | **0.49** | **89.6** | **3.61** |
| **PI378682C** | **Japan** | **IV** | **135.4** | **31.45** | **0.74** | **0.58** | **74.8** | **2.91** |
| **PI603494** | **China** | **IV** | **78.95** | **17.55** | **0.71** | **0.31** | **54.86** | **2.4** |
| **4J105-3-4** | **USA** | **III** | **91.06** | **20.49** | **0.72** | **0.37** | **49.33** | **2.12** |
| **5M20-2-5-2** | **USA** | **III** | **200.6** | **52.19** | **0.83** | **1.08** | **102.5** | **4.37** |
| **CL0J095-4-6** | **USA** | **III** | **125.1** | **25.35** | **0.64** | **0.41** | **90.6** | **6.05** |
| **LD02-9050** | **USA** | **IV** | **99.14** | **23.38** | **0.75** | **0.44** | **56.2** | **2.57** |
| **LG00-3372** | **USA** | **III** | **90** | **20.89** | **0.73** | **0.39** | **49.4** | **2.12** |
| **LG03-2979** | **USA** | **III** | **107.3** | **25.59** | **0.78** | **0.49** | **69.2** | **2.73** |
| **LG03-3191** | **USA** | **IV** | **96.22** | **20.38** | **0.68** | **0.35** | **57.33** | **3.71** |
| **LG04-4717** | **USA** | **III** | **85.5** | **22.5** | **0.84** | **0.47** | **37.6** | **0.64** |
| **LG05-4292** | **USA** | **IV** | **93.51** | **21.59** | **0.74** | **0.4** | **68** | **2.7** |
| **LG05-4317** | **USA** | **IV** | **96.72** | **26.15** | **0.86** | **0.56** | **55** | **1.04** |
| **LG05-4464** | **USA** | **III** | **225** | **49.79** | **0.72** | **0.88** | **92** | **6.19** |
| **LG05-4832** | **USA** | **III** | **114.7** | **31.76** | **0.88** | **0.7** | **56.8** | **1.06** |
| **LG90-2550** | **USA** | **III** | **98.09** | **20.87** | **0.67** | **0.35** | **44** | **2.85** |
| **LG94-1906** | **USA** | **II** | **54.7** | **11.42** | **0.66** | **0.19** | **33.5** | **2.36** |
| **LG98-1605** | **USA** | **III** | **110.6** | **28.67** | **0.83** | **0.59** | **56.4** | **1.01** |
| **NE3001** | **USA** | **III** | **145.2** | **39.95** | **0.87** | **0.88** | **87** | **1.98** |
| **PI404188A** | **China** | **II** | **72.36** | **18.34** | **0.8** | **0.37** | **47.25** | **1.33** |
| **Prohio** | **USA** | **III** | **150.6** | **35.57** | **0.75** | **0.67** | **87** | **1.9** |
| **S06-13640** | **USA** | **IV** | **58.7** | **14.02** | **0.74** | **0.27** | **34.25** | **0.85** |
| **TN05-3027** | **USA** | **V** | **103.9** | **26.73** | **0.81** | **0.55** | **63.2** | **1.45** |
| **U03-100612** | **USA** | **I** | **149.8** | **38.11** | **0.82** | **0.77** | **87** | **3.28** |
| **PI200503** | **Japan** | **V** | **91.65** | **21.22** | **0.73** | **0.39** | **46** | **1.38** |
| **PI209334** | **Japan** | **III** | **77.45** | **22.93** | **0.98** | **0.54** | **52** | **0.89** |
| **PI342434** | **Japan** | **V** | **87.58** | **17.59** | **0.64** | **0.28** | **58** | **3.65** |
| **PI377574** | **Japan** | **IV** | **64.01** | **14.4** | **0.72** | **0.26** | **45.5** | **2.25** |
| **PI416838** | **Japan** | **V** | **194.6** | **50.95** | **0.83** | **1.06** | **88.67** | **2.35** |
| **PI417007** | **Japan** | **IV** | **167.7** | **31.82** | **0.6** | **0.48** | **122.4** | **6.25** |
| **PI417015** | **Japan** | **III** | **159.9** | **35.28** | **0.69** | **0.62** | **91.25** | **3.52** |
| **PI417077** | **Japan** | **III** | **108.8** | **23.88** | **0.69** | **0.42** | **65.75** | **2.8** |
| **PI417479** | **Japan** | **IV** | **144.8** | **32.88** | **0.73** | **0.6** | **81.25** | **3.68** |
| **PI504288** | **Japan** | **V** | **39.13** | **8.17** | **0.66** | **0.14** | **26.5** | **0.82** |
| **PI506933** | **Japan** | **IV** | **69.96** | **16.2** | **0.75** | **0.3** | **44.5** | **1.57** |
| **PI548364** | **Japan** | **IV** | **140.1** | **33.39** | **0.78** | **0.64** | **80.25** | **2.88** |
| **PI561387** | **Japan** | **V** | **48.9** | **11.76** | **0.75** | **0.23** | **31** | **0.67** |
| **PI080837** | **Japan** | **IV** | **108.8** | **22.12** | **0.65** | **0.36** | **73.75** | **3.4** |
| **PI081041** | **Japan** | **III** | **135.9** | **30.91** | **0.73** | **0.56** | **71.5** | **2.8** |
| **PI081785** | **Japan** | **III** | **190.3** | **33.32** | **0.56** | **0.47** | **106.8** | **11.34** |
| **PI084631** | **SK** | **III** | **219.1** | **53.22** | **0.77** | **1.04** | **143.8** | **4.65** |
| **PI084973** | **Japan** | **III** | **188** | **46.33** | **0.77** | **0.92** | **114.3** | **3.2** |
| **PI086084** | **Japan** | **V** | **133.1** | **28.05** | **0.68** | **0.47** | **69** | **5.33** |
| **PI086982** | **SK** | **V** | **123.9** | **25.87** | **0.67** | **0.43** | **83.67** | **4.79** |
| **PI091083** | **SK** | **III** | **106.8** | **29.97** | **0.9** | **0.67** | **49** | **0.63** |
| **Line/Accession** | **Country of origin** | **Maturity group** | **TSA3_5DAT** | **TRW (cm)_5DAT** | **TCA (cm2)_5DAT** | **RDI_5DAT** | **SOL_5DAT** | **COPM_5DAT** |
| **Lee** | **USA** | **VI** | **2.87** | **11.67** | **97.07** | **0.6** | **0.1** | **4.93** |
| **Magellan** | **USA** | **IV** | **4.77** | **10.03** | **95.87** | **0.41** | **0.1** | **5.12** |
| **Maverick** | **USA** | **III** | **5.87** | **11.43** | **92.74** | **0.36** | **0.11** | **3.89** |
| **PI091725** | **NK** | **V** | **5.79** | **11.66** | **113** | **0.47** | **0.11** | **4.64** |
| **PI398595** | **SK** | **V** | **3.07** | **11.69** | **105.3** | **0.55** | **0.09** | **5.04** |
| **PI408105A** | **SK** | **IV** | **8.01** | **15.02** | **147.7** | **0.63** | **0.13** | **6.04** |
| **PI417242** | **China** | **II** | **7.13** | **16.27** | **158.5** | **0.55** | **0.09** | **5.45** |
| **PI438500** | **USA** | **III** | **7.92** | **17.01** | **156.3** | **0.67** | **0.12** | **5.93** |
| **PI467347** | **China** | **II** | **4.68** | **9.73** | **107.6** | **0.52** | **0.1** | **4.72** |
| **PI518668** | **USA** | **IV** | **7.01** | **14.5** | **131.2** | **0.58** | **0.11** | **5.2** |
| **PI548316** | **China** | **III** | **6.56** | **14.25** | **134.8** | **0.42** | **0.11** | **4.91** |
| **PI561271** | **China** | **V** | **5.12** | **14.84** | **172.8** | **0.47** | **0.09** | **6.12** |
| **PI567305** | **China** | **IV** | **3.63** | **12.13** | **125.1** | **0.5** | **0.08** | **5.22** |
| **PI567343** | **China** | **V** | **4.41** | **9.65** | **113.4** | **0.48** | **0.11** | **6.13** |
| **PI567651** | **China** | **IV** | **5.35** | **15.51** | **142.6** | **0.52** | **0.09** | **4.71** |
| **PI092728** | **China** | **III** | **4.77** | **13.9** | **153.8** | **0.52** | **0.1** | **5.68** |
| **PI518671 (Williams 82)** | **USA** | **III** | **3.67** | **13.66** | **125.1** | **0.52** | **0.1** | **4.94** |
| **PI087617** | **NK** | **III** | **10.9** | **18.4** | **239.4** | **0.35** | **0.11** | **6.27** |
| **PI196175** | **SK** | **V** | **6.67** | **11.2** | **110.9** | **0.54** | **0.12** | **5.36** |
| **PI209332** | **Japan** | **IV** | **1.76** | **8.88** | **99.51** | **0.37** | **0.07** | **5.2** |
| **PI248515** | **Japan** | **IV** | **1.43** | **6.99** | **74.39** | **0.51** | **0.07** | **5.59** |
| **PI398593** | **SK** | **V** | **4.64** | **12.51** | **153.4** | **0.66** | **0.1** | **7.67** |
| **PI398610** | **SK** | **V** | **3.43** | **9.3** | **93.77** | **0.35** | **0.08** | **4.37** |
| **PI407788A** | **SK** | **IV** | **8.05** | **12.74** | **141** | **0.55** | **0.13** | **5.41** |
| **PI407965** | **SK** | **V** | **7.06** | **12.8** | **187.7** | **0.39** | **0.09** | **6.83** |
| **PI424608A** | **SK** | **IV** | **10.14** | **17.32** | **201.5** | **0.63** | **0.1** | **7.04** |
| **PI458515** | **China** | **IV** | **4.94** | **13.8** | **125.4** | **0.38** | **0.1** | **4.25** |
| **PI495017C** | **China** | **IV** | **3.22** | **5.74** | **73.56** | **0.38** | **0.09** | **5.95** |
| **PI603154** | **NK** | **V** | **7.49** | **8.16** | **96.74** | **0.39** | **0.12** | **5.26** |
| **PI603175** | **NK** | **IV** | **5.38** | **10.4** | **94.03** | **0.47** | **0.11** | **4.99** |
| **PI605869A** | **Vietnam** | **V** | **5.66** | **15.28** | **141.4** | **0.46** | **0.1** | **5.29** |
| **PI404166** | **China** | **III** | **3.85** | **15.75** | **214.5** | **0.32** | **0.06** | **6.5** |
| **PI417091** | **Japan** | **II** | **4.48** | **14.31** | **161.3** | **0.43** | **0.09** | **6.6** |
| **PI437169B** | **Russia** | **II** | **6.12** | **13.42** | **141.4** | **0.28** | **0.1** | **4.53** |
| **PI437655** | **China** | **III** | **7.78** | **17.71** | **205.2** | **0.26** | **0.07** | **5.7** |
| **PI437725** | **China** | **IV** | **3.98** | **14.11** | **160.4** | **0.4** | **0.07** | **5.59** |
| **PI468915** | **China** | **II** | **4.91** | **14.45** | **172.7** | **0.37** | **0.07** | **5.69** |
| **PI548359** | **China** | **IV** | **7.69** | **11.78** | **121.1** | **0.33** | **0.1** | **4.7** |
| **PI548402 (peking)** | **China** | **IV** | **4.38** | **12.26** | **103.3** | **0.38** | **0.1** | **4.23** |
| **PI548427** | **China** | **IV** | **6.67** | **14.38** | **120.5** | **0.51** | **0.12** | **4.48** |
| **PI548619** | **USA** | **IV** | **5.72** | **15.13** | **139.9** | **0.53** | **0.09** | **4.85** |
| **PI548633** | **Japan** | **IV** | **5.36** | **7.73** | **79.73** | **0.4** | **0.1** | **5.19** |
| **PI548696** | **USA** | **V** | **5.62** | **14.26** | **174.5** | **0.45** | **0.09** | **5.93** |
| **PI556511** | **Japan** | **III** | **4.67** | **8.83** | **86.73** | **0.34** | **0.09** | **4.39** |
| **PI567387** | **China** | **IV** | **4.7** | **13.47** | **136.3** | **0.41** | **0.1** | **4.69** |
| **PI437487** | **Russia** | **III** | **1.93** | **8.73** | **65.54** | **0.49** | **0.07** | **4.01** |
| **PI533654** | **USA** | **IV** | **1.56** | **11.3** | **97.61** | **0.55** | **0.08** | **5.1** |
| **PI539936** | **USA** | **IV** | **2.13** | **12.02** | **101** | **0.54** | **0.09** | **5.03** |
| **PI540555** | **USA** | **IV** | **4.84** | **14.28** | **136.1** | **0.44** | **0.08** | **5.11** |
| **PI548158** | **USA** | **IV** | **0.76** | **6.7** | **54.3** | **0.43** | **0.08** | **4.08** |
| **PI548200** | **USA** | **IV** | **5.44** | **13.2** | **174.3** | **0.57** | **0.07** | **7.45** |
| **PI561701** | **USA** | **VI** | **1.45** | **7.33** | **74.79** | **0.53** | **0.09** | **5.82** |
| **PI378663** | **Russia** | **I** | **5.69** | **12.6** | **93.15** | **0.54** | **0.12** | **3.91** |
| **PI417550** | **Russia** | **0** | **3.76** | **11.9** | **103.9** | **0.5** | **0.1** | **4.91** |
| **PI437123** | **Russia** | **I** | **2.22** | **9.08** | **116.5** | **0.42** | **0.07** | **5.91** |
| **PI437138** | **Russia** | **0** | **4.74** | **12.4** | **91.42** | **0.47** | **0.12** | **4.13** |
| **PI437366** | **Russia** | **I** | **2.57** | **12.31** | **138.1** | **0.32** | **0.07** | **4.95** |
| **PI437476** | **Russia** | **III** | **2.48** | **11.02** | **84.05** | **0.5** | **0.09** | **4.02** |
| **PI507678** | **Russia** | **I** | **1.8** | **10.18** | **75.64** | **0.45** | **0.09** | **4.05** |
| **PI548169** | **USA** | **IV** | **3.43** | **18.98** | **225.4** | **0.45** | **0.06** | **5.55** |
| **PI548178** | **USA** | **III** | **2.76** | **7.55** | **59.29** | **0.45** | **0.12** | **3.69** |
| **PI548313** | **Russia** | **III** | **2.34** | **8.79** | **73.33** | **0.27** | **0.09** | **3.67** |
| **PI548325** | **Russia** | **0** | **3.8** | **9.91** | **113.1** | **0.45** | **0.11** | **5.6** |
| **PI548336** | **Russia** | **I** | **1.79** | **12.07** | **116.7** | **0.44** | **0.1** | **4.73** |
| **PI556637** | **USA** | **I** | **2.63** | **14.77** | **147.6** | **0.48** | **0.09** | **5.72** |
| **PI567226** | **Russia** | **0** | **3.05** | **11.82** | **88.63** | **0.4** | **0.11** | **3.76** |
| **PI597402** | **Russia** | **0** | **3.36** | **9.41** | **88.94** | **0.33** | **0.11** | **4.06** |
| **PI062202-2** | **China** | **IV** | **6.34** | **11.87** | **136.2** | **0.58** | **0.13** | **6.15** |
| **PI063945** | **China** | **IV** | **2.06** | **10.25** | **91.12** | **0.55** | **0.1** | **4.84** |
| **PI079616** | **China** | **III** | **4.1** | **10.3** | **83.98** | **0.43** | **0.11** | **4.15** |
| **PI084509** | **Unknown** | **III** | **1.6** | **8.56** | **70.74** | **0.48** | **0.09** | **4.31** |
| **PI088306** | **China** | **III** | **2.2** | **8.18** | **64.91** | **0.48** | **0.11** | **4.41** |
| **PI090723** | **Unknown** | **III** | **2.92** | **12.57** | **105.6** | **0.35** | **0.08** | **4.09** |
| **PI091731-1** | **China** | **IV** | **6.77** | **12.09** | **132.1** | **0.27** | **0.09** | **4.18** |
| **PI504495** | **Taiwan** | **V** | **1.97** | **8.89** | **88.02** | **0.35** | **0.09** | **4.03** |
| **PI593258** | **USA** | **III** | **5.21** | **11.45** | **90.01** | **0.47** | **0.12** | **4.11** |
| **PI525454** | **USA** | **IV** | **3.79** | **13.74** | **130.2** | **0.46** | **0.08** | **5.05** |
| **PI548193** | **USA** | **IV** | **5.62** | **10.06** | **80.96** | **0.56** | **0.13** | **4.5** |
| **PI548511** | **USA** | **II** | **4.88** | **7.56** | **78.18** | **0.52** | **0.11** | **5.5** |
| **PI548547** | **USA** | **IV** | **7.45** | **11.19** | **106.5** | **0.44** | **0.11** | **4.84** |
| **PI559932** | **USA** | **IV** | **2.91** | **7.05** | **56.66** | **0.47** | **0.12** | **4.28** |
| **PI597384** | **USA** | **IV** | **2.07** | **7.64** | **64.27** | **0.24** | **0.08** | **3.75** |
| **PI060970** | **China** | **IV** | **5.82** | **10.71** | **96.43** | **0.59** | **0.11** | **5.17** |
| **PI070242-2** | **China** | **IV** | **11.7** | **20.57** | **167.4** | **0.66** | **0.13** | **5.62** |
| **PI079797** | **China** | **III** | **5.73** | **12.91** | **113.2** | **0.57** | **0.12** | **5.19** |
| **PI088448** | **China** | **III** | **4.46** | **11.58** | **102.9** | **0.64** | **0.13** | **5.35** |
| **PI090369** | **China** | **IV** | **5.48** | **10.85** | **101.9** | **0.62** | **0.12** | **4.94** |
| **PI180501** | **Germany** | **0** | **5.4** | **12.77** | **117.1** | **0.56** | **0.1** | **5.63** |
| **PI360957** | **Japan** | **0** | **4.35** | **9.04** | **84.74** | **0.71** | **0.11** | **6.05** |
| **PI404161** | **Georgia** | **IV** | **3.16** | **8.82** | **84.73** | **0.45** | **0.1** | **4.42** |
| **PI416751** | **Japan** | **I** | **6.05** | **13.22** | **125.7** | **0.55** | **0.1** | **5.16** |
| **PI417529** | **Germany** | **0** | **3.43** | **10.05** | **71.68** | **0.67** | **0.11** | **4.6** |
| **PI438312** | **Algeria** | **III** | **4.34** | **16.2** | **165.3** | **0.52** | **0.09** | **5.34** |
| **PI518751** | **Former Serbia and Montenegro** | **II** | **6.87** | **12.96** | **133.9** | **0.43** | **0.12** | **5.17** |
| **PI548414** | **Japan** | **0** | **6.36** | **14.63** | **113.5** | **0.54** | **0.12** | **4.94** |
| **PI068521_1** | **China** | **II** | **3.71** | **9.85** | **98.64** | **0.43** | **0.09** | **4.9** |
| **PI068679-2** | **China** | **IV** | **6.79** | **9.38** | **124.5** | **0.6** | **0.1** | **7.32** |
| **PI081042-2** | **Japan** | **IV** | **6.55** | **6.13** | **85.94** | **0.64** | **0.14** | **7.49** |
| **PI089772** | **China** | **IV** | **5.02** | **11.62** | **119.2** | **0.48** | **0.11** | **5.47** |
| **PI153281** | **Belgium** | **0** | **3.81** | **13.89** | **126.4** | **0.65** | **0.1** | **5.8** |
| **PI209331** | **Japan** | **III** | **4.96** | **3.64** | **45.98** | **0.6** | **0.17** | **5.86** |
| **PI290136** | **France** | **0** | **6.75** | **6.89** | **81.26** | **0.48** | **0.15** | **5.93** |
| **PI438323** | **France** | **I** | **5.66** | **10.56** | **139.3** | **0.61** | **0.1** | **6.98** |
| **PI438335** | **Algeria** | **III** | **5.1** | **11.75** | **128.6** | **0.63** | **0.1** | **6.64** |
| **PI507467** | **Japan** | **IV** | **7.78** | **9.98** | **111** | **0.54** | **0.16** | **5.76** |
| **PI087571** | **NK** | **IV** | **5.95** | **5.58** | **56.6** | **0.51** | **0.17** | **4.86** |
| **PI087618** | **NK** | **III** | **8.24** | **10.9** | **122.2** | **0.52** | **0.11** | **5.75** |
| **PI154189** | **Netherlands** | **0** | **4.04** | **6.76** | **65.45** | **0.51** | **0.11** | **5.46** |
| **PI198067** | **Sweden** | **0** | **4.74** | **9.08** | **106.8** | **0.53** | **0.12** | **6.1** |
| **PI361093** | **Serbia** | **I** | **6.54** | **5.12** | **53.6** | **0.54** | **0.16** | **6.13** |
| **PI372418** | **Serbia** | **I** | **4.91** | **9.13** | **104.6** | **0.52** | **0.11** | **5.55** |
| **PI398614** | **SK** | **V** | **1.86** | **6.97** | **57.39** | **0.42** | **0.11** | **4.05** |
| **PI398633** | **SK** | **V** | **3.03** | **9.22** | **93.04** | **0.49** | **0.1** | **4.97** |
| **PI398965** | **SK** | **IV** | **3.49** | **8.79** | **85.18** | **0.42** | **0.12** | **4.94** |
| **PI408088** | **SK** | **V** | **4.35** | **8.58** | **97.72** | **0.44** | **0.1** | **5.45** |
| **PI424005** | **SK** | **III** | **1.34** | **6.32** | **69** | **0.39** | **0.1** | **5.12** |
| **PI430595** | **China** | **IV** | **5.06** | **13.71** | **135.5** | **0.55** | **0.12** | **5.33** |
| **PI437287** | **Moldova** | **0** | **7.36** | **9.1** | **119.2** | **0.56** | **0.11** | **6.92** |
| **PI437296** | **Moldova** | **0** | **3.55** | **5.59** | **52.61** | **0.48** | **0.14** | **5.23** |
| **PI567225** | **Moldova** | **0** | **7.13** | **8.01** | **85.92** | **0.4** | **0.12** | **5.13** |
| **PI597476** | **SK** | **V** | **2.11** | **8.72** | **85.99** | **0.51** | **0.1** | **5.82** |
| **PI612611** | **NK** | **III** | **10.75** | **18.22** | **202.3** | **0.62** | **0.1** | **6.88** |
| **Holladay (S-100)** | **USA** | **V** | **3.36** | **10.12** | **74.43** | **0.38** | **0.11** | **3.87** |
| **IA3023** | **USA** | **III** | **3.6** | **12.04** | **82.5** | **0.47** | **0.1** | **3.52** |
| **PI088788** | **China** | **III** | **2.88** | **8.14** | **69.69** | **0.51** | **0.09** | **4.84** |
| **PI090763** | **China** | **IV** | **2.89** | **7.27** | **52.89** | **0.52** | **0.1** | **4.17** |
| **PI404198B** | **China** | **IV** | **2.26** | **9.49** | **84.43** | **0.32** | **0.07** | **4.39** |
| **PI407729** | **China** | **IV** | **4.13** | **9.96** | **86.29** | **0.3** | **0.1** | **4.13** |
| **PI437654** | **China** | **III** | **3.31** | **11.3** | **98.48** | **0.45** | **0.1** | **4.95** |
| **PI437679** | **China** | **IV** | **4.99** | **10.33** | **83.76** | **0.35** | **0.11** | **4.09** |
| **PI437690** | **China** | **III** | **1.09** | **8.13** | **65.25** | **0.34** | **0.08** | **4.11** |
| **PI438258** | **China** | **II** | **5.51** | **12.05** | **94.46** | **0.46** | **0.12** | **3.9** |
| **PI467312** | **China** | **II** | **3.08** | **8.5** | **73.86** | **0.36** | **0.09** | **4.19** |
| **PI475783B** | **China** | **III** | **6.11** | **11.33** | **109.4** | **0.32** | **0.11** | **4.39** |
| **PI547862** | **USA** | **III** | **5.44** | **9.06** | **79.63** | **0.3** | **0.1** | **3.84** |
| **PI548317** | **China** | **III** | **2.76** | **7.84** | **63.2** | **0.38** | **0.09** | **3.93** |
| **PI548415** | **China** | **IV** | **2.38** | **9.82** | **74.19** | **0.37** | **0.08** | **3.91** |
| **PI567516C** | **China** | **IV** | **1.73** | **9.44** | **75.8** | **0.39** | **0.07** | **3.99** |
| **FC033243** | **Algeria** | **IV** | **3.15** | **7.48** | **51.26** | **0.45** | **0.11** | **3.72** |
| **PI549018** | **China** | **V** | **0.86** | **6.69** | **49.12** | **0.44** | **0.09** | **4.02** |
| **PI549031** | **China** | **III** | **3.44** | **10.92** | **95** | **0.23** | **0.09** | **4.07** |
| **PI552538** | **USA** | **III** | **7.94** | **8.96** | **81.14** | **0.47** | **0.13** | **4.44** |
| **PI567230** | **China** | **V** | **1.12** | **7.03** | **57.58** | **0.35** | **0.08** | **3.92** |
| **PI567336B** | **China** | **IV** | **1.22** | **8.41** | **71.47** | **0.23** | **0.08** | **3.71** |
| **PI567354** | **China** | **IV** | **5.48** | **11.16** | **95.27** | **0.31** | **0.13** | **4.47** |
| **PI567357** | **China** | **III** | **9.9** | **15.95** | **131.3** | **0.42** | **0.13** | **4.35** |
| **PI567383** | **China** | **V** | **6.56** | **11.86** | **103.5** | **0.43** | **0.1** | **4.33** |
| **PI567519** | **China** | **III** | **3.84** | **9.77** | **67.98** | **0.34** | **0.1** | **3.66** |
| **PI567611** | **China** | **IV** | **1.82** | **5.08** | **47.09** | **0.54** | **0.12** | **5.34** |
| **PI567690** | **China** | **III** | **2.49** | **6.65** | **60.95** | **0.26** | **0.09** | **4.13** |
| **PI567731** | **China** | **III** | **2.69** | **7.5** | **58.8** | **0.42** | **0.09** | **4.04** |
| **PI594599** | **China** | **IV** | **4.04** | **10.64** | **98.34** | **0.34** | **0.11** | **4.61** |
| **PI597387** | **USA** | **III** | **4.67** | **10.53** | **93.59** | **0.35** | **0.09** | **4.09** |
| **PI603176A** | **NK** | **IV** | **3.72** | **9.17** | **66.49** | **0.6** | **0.13** | **4.21** |
| **PI639740 (LD00-3309)** | **USA** | **IV** | **4.56** | **10.88** | **94.4** | **0.39** | **0.1** | **4.23** |
| **PI092651** | **China** | **IV** | **5.67** | **13.15** | **80.19** | **0.62** | **0.15** | **3.66** |
| **PI103088** | **China** | **III** | **6.98** | **13.06** | **97.1** | **0.44** | **0.16** | **3.91** |
| **PI153231** | **China** | **III** | **8.94** | **12.42** | **104.2** | **0.26** | **0.13** | **4.13** |
| **PI171428** | **China** | **IV** | **5.58** | **10.78** | **87.77** | **0.6** | **0.14** | **4.38** |
| **PI253661B** | **China** | **III** | **4.47** | **11.67** | **102.6** | **0.43** | **0.11** | **4.43** |
| **PI379618** | **China** | **V** | **5.74** | **11.51** | **86.08** | **0.59** | **0.14** | **3.98** |
| **PI407742** | **China** | **V** | **3.95** | **12.21** | **106.1** | **0.44** | **0.1** | **4.47** |
| **PI417581** | **China** | **V** | **5.37** | **11.16** | **79.96** | **0.74** | **0.12** | **4.64** |
| **PI424038B** | **China** | **V** | **0.98** | **7.69** | **74.01** | **0.26** | **0.07** | **4.25** |
| **PI437110A** | **China** | **III** | **4.18** | **10.24** | **70.34** | **0.6** | **0.14** | **3.89** |
| **PI437127A** | **China** | **IV** | **6.36** | **13.49** | **117.6** | **0.56** | **0.13** | **4.66** |
| **PI437685D** | **China** | **III** | **5.21** | **10.03** | **75.53** | **0.34** | **0.13** | **3.72** |
| **PI437776** | **China** | **III** | **7.81** | **12.09** | **123.5** | **0.3** | **0.14** | **4.4** |
| **FC029333** | **China** | **III** | **7.55** | **12.8** | **100.6** | **0.54** | **0.13** | **4.51** |
| **FC031697** | **China** | **IV** | **5.74** | **12.31** | **104.2** | **0.45** | **0.09** | **4.03** |
| **PI054615_1** | **China** | **III** | **5.82** | **10.58** | **85.54** | **0.64** | **0.14** | **4.7** |
| **PI058955** | **China** | **IV** | **2.06** | **10.4** | **71.93** | **0.46** | **0.1** | **3.85** |
| **PI068604_1** | **China** | **III** | **3.77** | **9.49** | **87.09** | **0.37** | **0.11** | **4.35** |
| **PI070466_3** | **China** | **IV** | **6.51** | **12.6** | **107.1** | **0.39** | **0.11** | **4.45** |
| **PI071465** | **China** | **V** | **6.48** | **14.44** | **132.1** | **0.41** | **0.12** | **4.44** |
| **PI083925** | **SK** | **IV** | **3.55** | **13.14** | **113.4** | **0.45** | **0.11** | **4.31** |
| **PI084946_2** | **China** | **IV** | **13.1** | **20.27** | **166.3** | **0.7** | **0.17** | **5.29** |
| **PI086972_2** | **China** | **IV** | **7.17** | **17.1** | **170.6** | **0.32** | **0.12** | **4.82** |
| **PI090479P** | **China** | **IV** | **5.03** | **8.43** | **72.17** | **0.35** | **0.14** | **4.29** |
| **PI090486** | **China** | **III** | **4.4** | **9.38** | **77.77** | **0.42** | **0.12** | **4.28** |
| **PI567426** | **China** | **IV** | **4.5** | **12.59** | **118.2** | **0.45** | **0.11** | **5.42** |
| **PI567558** | **China** | **III** | **5.39** | **14.93** | **144** | **0.44** | **0.11** | **5.1** |
| **PI578495** | **China** | **IV** | **5.7** | **10.5** | **104.1** | **0.41** | **0.12** | **4.52** |
| **PI603442** | **China** | **III** | **5.92** | **11.22** | **91.38** | **0.41** | **0.14** | **3.96** |
| **PI468408B** | **China** | **III** | **4.47** | **9.36** | **76.77** | **0.41** | **0.12** | **4.15** |
| **PI479735** | **France** | **III** | **5.76** | **9.57** | **95.33** | **0.24** | **0.1** | **4.71** |
| **PI490766** | **Georgia** | **III** | **8.14** | **11.5** | **124.2** | **0.3** | **0.11** | **4.61** |
| **PI495020** | **Germany** | **IV** | **2.11** | **5.99** | **56.15** | **0.29** | **0.1** | **3.91** |
| **PI561371** | **Moldova** | **IV** | **4.87** | **6.83** | **64.86** | **0.56** | **0.13** | **5.66** |
| **PI567307** | **NK** | **IV** | **2.36** | **8.2** | **93.22** | **0.39** | **0.08** | **6.1** |
| **PI567415A** | **Russia** | **IV** | **4.76** | **7.36** | **63.24** | **0.59** | **0.13** | **4.18** |
| **PI567416** | **Russia** | **IV** | **4.68** | **5.9** | **68.34** | **0.5** | **0.13** | **6.12** |
| **PI567428** | **Russia** | **IV** | **3.22** | **7.34** | **82.99** | **0.37** | **0.09** | **5.84** |
| **PI567435B** | **Russia** | **III** | **5.88** | **10.88** | **96.83** | **0.46** | **0.11** | **4.26** |
| **PI567439** | **Russia** | **V** | **7.18** | **9.49** | **99.96** | **0.35** | **0.1** | **4.6** |
| **PI567488A** | **Russia** | **IV** | **5.28** | **8.59** | **74.46** | **0.52** | **0.16** | **4.07** |
| **PI567532** | **Russia** | **IV** | **3.82** | **10.24** | **75.92** | **0.47** | **0.12** | **4.04** |
| **PI567548** | **Russia** | **IV** | **3.25** | **6.57** | **70.43** | **0.41** | **0.1** | **5.14** |
| **PI567576** | **Russia** | **III** | **5.77** | **9.4** | **83.3** | **0.51** | **0.16** | **4.03** |
| **PI567780B** | **China** | **IV** | **2.67** | **9.99** | **104.4** | **0.29** | **0.08** | **4.64** |
| **PI538386A** | **Japan** | **III** | **3.72** | **10.96** | **137.7** | **0.3** | **0.09** | **5.68** |
| **PI548383** | **Japan** | **III** | **4.34** | **8.91** | **94.48** | **0.26** | **0.09** | **4.72** |
| **PI548400** | **Japan** | **IV** | **4.17** | **13.8** | **103.7** | **0.46** | **0.1** | **4.23** |
| **PI567346** | **NK** | **V** | **2.83** | **10.14** | **79.52** | **0.32** | **0.09** | **3.96** |
| **PI567352A** | **Peru** | **IV** | **8.75** | **10.82** | **95.09** | **0.28** | **0.15** | **4.14** |
| **PI567353** | **Philippines** | **IV** | **3.98** | **13.7** | **100.4** | **0.6** | **0.12** | **4.43** |
| **PI567361** | **Philippines** | **III** | **6.78** | **19.53** | **236.9** | **0.34** | **0.07** | **5.69** |
| **PI567407** | **Romania** | **V** | **6.46** | **10.71** | **142.3** | **0.29** | **0.11** | **5.68** |
| **PI567408** | **Russia** | **V** | **5.07** | **9.87** | **96.82** | **0.31** | **0.11** | **4.45** |
| **PI567675** | **Russia** | **IV** | **3.19** | **6.4** | **64.9** | **0.36** | **0.13** | **4.9** |
| **PI567685** | **Russia** | **IV** | **2.46** | **7.22** | **70.88** | **0.36** | **0.1** | **4.43** |
| **PI567698A** | **Russia** | **IV** | **3.32** | **9.08** | **89.21** | **0.34** | **0.11** | **4** |
| **PI567726** | **Russia** | **IV** | **3.83** | **10.17** | **119.5** | **0.35** | **0.09** | **5.5** |
| **PI567746** | **Russia** | **IV** | **2.11** | **7.13** | **53.48** | **0.36** | **0.12** | **4.21** |
| **PI574477** | **SK** | **IV** | **6.27** | **12.11** | **114.9** | **0.54** | **0.11** | **4.84** |
| **PI587588B** | **SK** | **V** | **3.63** | **9.29** | **105.3** | **0.37** | **0.1** | **5.76** |
| **PI587712B** | **SK** | **V** | **3.13** | **8.45** | **72.13** | **0.39** | **0.13** | **4.12** |
| **PI068423** | **China** | **III** | **2.92** | **7.37** | **78.56** | **0.38** | **0.09** | **5.25** |
| **PI068523** | **China** | **III** | **4.71** | **9.75** | **83.79** | **0.58** | **0.11** | **4.88** |
| **PI068732_1** | **China** | **II** | **3.29** | **10.38** | **85.58** | **0.55** | **0.1** | **4.31** |
| **PI297505** | **China** | **I** | **2.38** | **5.7** | **58.31** | **0.36** | **0.1** | **4.58** |
| **PI417381** | **Japan** | **0** | **3.12** | **8.38** | **72.17** | **0.48** | **0.1** | **4.36** |
| **PI603495B** | **China** | **V** | **5.06** | **12.04** | **77.47** | **0.51** | **0.12** | **3.87** |
| **PI603526** | **China** | **IV** | **5.08** | **13.24** | **102.6** | **0.43** | **0.11** | **4.38** |
| **PI603549** | **China** | **III** | **4.63** | **12.68** | **122.4** | **0.38** | **0.1** | **4.52** |
| **PI603556** | **China** | **III** | **3.67** | **11.81** | **84.35** | **0.56** | **0.1** | **3.99** |
| **PI070208** | **China** | **IV** | **2.9** | **9.28** | **87.21** | **0.29** | **0.1** | **4.5** |
| **PI079870-4** | **China** | **IV** | **1.92** | **8.06** | **45.82** | **0.57** | **0.09** | **3.46** |
| **PI430598B** | **China** | **IV** | **4.93** | **11.07** | **84.64** | **0.49** | **0.13** | **4.17** |
| **PI594456A** | **China** | **III** | **1.2** | **7.51** | **63.29** | **0.37** | **0.09** | **4.06** |
| **PI594880** | **China** | **V** | **2.02** | **7.1** | **52.5** | **0.45** | **0.11** | **3.66** |
| **PI594922** | **USA** | **V** | **7.71** | **12.02** | **98.41** | **0.33** | **0.13** | **4.19** |
| **PI597478B** | **SK** | **III** | **3.25** | **10.52** | **64.25** | **0.54** | **0.11** | **3.51** |
| **PI598358** | **Unknown** | **V** | **2.05** | **7.99** | **53.68** | **0.36** | **0.11** | **3.01** |
| **PI606374** | **Vietnam** | **III** | **0.6** | **5.84** | **30.86** | **0.61** | **0.1** | **2.84** |
| **PI632418** | **USA** | **V** | **3.6** | **8.73** | **65** | **0.4** | **0.1** | **3.67** |
| **PI093055S** | **China** | **V** | **2.04** | **7.49** | **63.88** | **0.48** | **0.12** | **4.98** |
| **PI093563** | **China** | **III** | **4.29** | **11.81** | **116.2** | **0.31** | **0.11** | **4.61** |
| **PI103079** | **China** | **V** | **1.61** | **8.41** | **75.66** | **0.44** | **0.11** | **4.5** |
| **PI123587** | **Belgium** | **V** | **2.11** | **9.23** | **110.5** | **0.51** | **0.1** | **5.52** |
| **PI235347** | **Japan** | **IV** | **2.36** | **14.32** | **193.9** | **0.36** | **0.08** | **6.64** |
| **PI253656B** | **China** | **IV** | **2.93** | **8.08** | **78.23** | **0.35** | **0.12** | **4.98** |
| **PI378682C** | **Japan** | **IV** | **3.99** | **9.42** | **101.3** | **0.38** | **0.1** | **4.9** |
| **PI603494** | **China** | **IV** | **1.4** | **6.65** | **61.06** | **0.44** | **0.1** | **4.72** |
| **4J105-3-4** | **USA** | **III** | **1.59** | **9.14** | **77.75** | **0.37** | **0.09** | **4.45** |
| **5M20-2-5-2** | **USA** | **III** | **7.59** | **14.61** | **125.6** | **0.35** | **0.13** | **4.1** |
| **CL0J095-4-6** | **USA** | **III** | **2.69** | **11.02** | **107.2** | **0.25** | **0.08** | **4.48** |
| **LD02-9050** | **USA** | **IV** | **4.33** | **8.84** | **77.18** | **0.31** | **0.1** | **3.77** |
| **LG00-3372** | **USA** | **III** | **3.09** | **9.63** | **63.84** | **0.4** | **0.11** | **3.47** |
| **LG03-2979** | **USA** | **III** | **2.99** | **12.31** | **109.9** | **0.27** | **0.08** | **3.81** |
| **LG03-3191** | **USA** | **IV** | **2.3** | **8.24** | **66.19** | **0.31** | **0.1** | **3.49** |
| **LG04-4717** | **USA** | **III** | **3.55** | **10.09** | **59.53** | **0.61** | **0.12** | **3.61** |
| **LG05-4292** | **USA** | **IV** | **2.76** | **9.38** | **60.72** | **0.42** | **0.11** | **3.46** |
| **LG05-4317** | **USA** | **IV** | **4.47** | **11.56** | **85.42** | **0.49** | **0.1** | **4.47** |
| **LG05-4464** | **USA** | **III** | **5.19** | **13.62** | **170.3** | **0.4** | **0.1** | **5.81** |
| **LG05-4832** | **USA** | **III** | **5.7** | **13.1** | **96.07** | **0.44** | **0.11** | **3.94** |
| **LG90-2550** | **USA** | **III** | **2.79** | **10.61** | **83.55** | **0.41** | **0.08** | **3.87** |
| **LG94-1906** | **USA** | **II** | **1.4** | **6.81** | **41.19** | **0.36** | **0.09** | **2.97** |
| **LG98-1605** | **USA** | **III** | **4.27** | **12.97** | **99.12** | **0.39** | **0.09** | **3.77** |
| **NE3001** | **USA** | **III** | **6.7** | **12.26** | **99.86** | **0.33** | **0.13** | **3.79** |
| **PI404188A** | **China** | **II** | **3.82** | **6.38** | **49.77** | **0.4** | **0.12** | **3.48** |
| **Prohio** | **USA** | **III** | **5.35** | **8.86** | **95.19** | **0.35** | **0.12** | **5.09** |
| **S06-13640** | **USA** | **IV** | **1.98** | **7.11** | **55.2** | **0.42** | **0.08** | **3.66** |
| **TN05-3027** | **USA** | **V** | **4.78** | **8.98** | **75.91** | **0.22** | **0.11** | **3.68** |
| **U03-100612** | **USA** | **I** | **6.14** | **9.6** | **96.47** | **0.27** | **0.12** | **3.86** |
| **PI200503** | **Japan** | **V** | **3.27** | **8.65** | **73.35** | **0.46** | **0.09** | **4.02** |
| **PI209334** | **Japan** | **III** | **4.16** | **7.79** | **64.28** | **0.43** | **0.12** | **3.87** |
| **PI342434** | **Japan** | **V** | **2.17** | **7.65** | **46.5** | **0.58** | **0.12** | **3.89** |
| **PI377574** | **Japan** | **IV** | **1.36** | **6.05** | **44.11** | **0.43** | **0.1** | **3.98** |
| **PI416838** | **Japan** | **V** | **7.6** | **13.05** | **119.8** | **0.53** | **0.13** | **4.58** |
| **PI417007** | **Japan** | **IV** | **1.73** | **12.36** | **145.2** | **0.33** | **0.07** | **5.71** |
| **PI417015** | **Japan** | **III** | **2.92** | **10.78** | **107.9** | **0.38** | **0.11** | **5.3** |
| **PI417077** | **Japan** | **III** | **3.06** | **10.48** | **82.86** | **0.42** | **0.09** | **4.35** |
| **PI417479** | **Japan** | **IV** | **4.25** | **9.1** | **90.97** | **0.41** | **0.11** | **5.2** |
| **PI504288** | **Japan** | **V** | **1.4** | **5.89** | **34.11** | **0.51** | **0.08** | **3.25** |
| **PI506933** | **Japan** | **IV** | **2.94** | **8.67** | **41.55** | **0.67** | **0.12** | **3.2** |
| **PI548364** | **Japan** | **IV** | **4.17** | **12.92** | **94.28** | **0.65** | **0.12** | **4.38** |
| **PI561387** | **Japan** | **V** | **2.49** | **6.11** | **42.6** | **0.61** | **0.09** | **3.98** |
| **PI080837** | **Japan** | **IV** | **1.22** | **9.94** | **121.7** | **0.35** | **0.06** | **5.97** |
| **PI081041** | **Japan** | **III** | **4.35** | **11.86** | **101.7** | **0.41** | **0.1** | **4.02** |
| **PI081785** | **Japan** | **III** | **1.77** | **10.19** | **118.1** | **0.43** | **0.09** | **6.2** |
| **PI084631** | **SK** | **III** | **7.27** | **14.12** | **180.8** | **0.42** | **0.11** | **6.1** |
| **PI084973** | **Japan** | **III** | **6.46** | **14.41** | **137.2** | **0.39** | **0.11** | **4.9** |
| **PI086084** | **Japan** | **V** | **4.13** | **7.58** | **85.86** | **0.4** | **0.1** | **5.8** |
| **PI086982** | **SK** | **V** | **2.58** | **9.35** | **103.1** | **0.41** | **0.08** | **5.9** |
| **PI091083** | **SK** | **III** | **5** | **12.32** | **94.01** | **0.44** | **0.1** | **4** |
| **Line/Accession** | **Country of origin** | **Maturity group** | **TRL (cm)_8DAT** | **TSA (cm2)_8DAT** | **DIM (mm)_8DAT** | **TRV (cm3)_8DAT** | **TRT_8DAT** | **TSA1_8DAT** |
| **Lee** | **USA** | **VI** | **250.6** | **53.51** | **0.68** | **0.92** | **171** | **9.76** |
| **Magellan** | **USA** | **IV** | **289.8** | **69.56** | **0.77** | **1.34** | **139.7** | **11.68** |
| **Maverick** | **USA** | **III** | **214.5** | **52.79** | **0.79** | **1.04** | **139.5** | **6.85** |
| **PI091725** | **NK** | **V** | **403.9** | **84.42** | **0.67** | **1.4** | **248.5** | **15.07** |
| **PI398595** | **SK** | **V** | **275.9** | **54.54** | **0.63** | **0.86** | **128.2** | **14.16** |
| **PI408105A** | **SK** | **IV** | **524.1** | **108** | **0.66** | **1.78** | **276.3** | **28.72** |
| **PI417242** | **China** | **II** | **281.8** | **73.22** | **0.85** | **1.52** | **195** | **8.06** |
| **PI438500** | **USA** | **III** | **605.3** | **121.7** | **0.64** | **1.96** | **273.3** | **34.1** |
| **PI467347** | **China** | **II** | **167.1** | **35.99** | **0.69** | **0.62** | **125.3** | **7.01** |
| **PI518668** | **USA** | **IV** | **372.8** | **78.1** | **0.66** | **1.31** | **215.3** | **17.69** |
| **PI548316** | **China** | **III** | **332** | **75.65** | **0.73** | **1.39** | **217.5** | **15.23** |
| **PI561271** | **China** | **V** | **375** | **75.35** | **0.65** | **1.21** | **161.6** | **21.8** |
| **PI567305** | **China** | **IV** | **237.8** | **51.67** | **0.71** | **0.91** | **152.5** | **10.91** |
| **PI567343** | **China** | **V** | **306.8** | **64.33** | **0.71** | **1.09** | **212.2** | **15.28** |
| **PI567651** | **China** | **IV** | **296.2** | **64.09** | **0.7** | **1.12** | **172** | **14.26** |
| **PI092728** | **China** | **III** | **295** | **64.49** | **0.7** | **1.12** | **212.7** | **11.97** |
| **PI518671 (Williams 82)** | **USA** | **III** | **292.2** | **60.42** | **0.66** | **1** | **189.4** | **16.5** |
| **PI087617** | **NK** | **III** | **739.9** | **162.2** | **0.71** | **2.85** | **290.5** | **46.1** |
| **PI196175** | **SK** | **V** | **365.5** | **78.11** | **0.68** | **1.33** | **152.2** | **21.04** |
| **PI209332** | **Japan** | **IV** | **144.5** | **28.22** | **0.62** | **0.44** | **107.4** | **9.35** |
| **PI248515** | **Japan** | **IV** | **212.3** | **40.73** | **0.61** | **0.63** | **98.17** | **14.3** |
| **PI398593** | **SK** | **V** | **564.3** | **105.9** | **0.6** | **1.59** | **184.8** | **37.37** |
| **PI398610** | **SK** | **V** | **205.2** | **46.17** | **0.74** | **0.84** | **122.5** | **8.54** |
| **PI407788A** | **SK** | **IV** | **550.7** | **111.5** | **0.65** | **1.81** | **368.7** | **30.35** |
| **PI407965** | **SK** | **V** | **553.8** | **111.7** | **0.64** | **1.8** | **309.8** | **31.35** |
| **PI424608A** | **SK** | **IV** | **666.5** | **143.1** | **0.68** | **2.46** | **291** | **36.76** |
| **PI458515** | **China** | **IV** | **422.6** | **91.25** | **0.76** | **1.61** | **239** | **21.15** |
| **PI495017C** | **China** | **IV** | **267.4** | **65.08** | **0.77** | **1.26** | **126.5** | **10.1** |
| **PI603154** | **NK** | **V** | **301.7** | **74.73** | **0.79** | **1.49** | **119.5** | **12.29** |
| **PI603175** | **NK** | **IV** | **367.5** | **81.61** | **0.71** | **1.45** | **143.8** | **17.37** |
| **PI605869A** | **Vietnam** | **V** | **615.8** | **110.4** | **0.57** | **1.58** | **330.3** | **43.31** |
| **PI404166** | **China** | **III** | **280.3** | **56.11** | **0.65** | **0.91** | **167.3** | **14.28** |
| **PI417091** | **Japan** | **II** | **364** | **69.79** | **0.62** | **1.08** | **158.2** | **21.73** |
| **PI437169B** | **Russia** | **II** | **350.2** | **80.84** | **0.74** | **1.5** | **201** | **14.28** |
| **PI437655** | **China** | **III** | **261.3** | **64.27** | **0.79** | **1.27** | **121.4** | **4.86** |
| **PI437725** | **China** | **IV** | **268.2** | **51.32** | **0.61** | **0.8** | **165** | **15.7** |
| **PI468915** | **China** | **II** | **347.9** | **65.48** | **0.6** | **0.99** | **188** | **20.08** |
| **PI548359** | **China** | **IV** | **224** | **56.55** | **0.81** | **1.14** | **107.2** | **7.15** |
| **PI548402 (peking)** | **China** | **IV** | **204.8** | **47.76** | **0.74** | **0.89** | **145.2** | **7.47** |
| **PI548427** | **China** | **IV** | **360** | **82.56** | **0.73** | **1.51** | **203** | **14.8** |
| **PI548619** | **USA** | **IV** | **304.1** | **65.74** | **0.7** | **1.15** | **210.5** | **11.09** |
| **PI548633** | **Japan** | **IV** | **118.7** | **31.52** | **0.84** | **0.67** | **89.83** | **2.89** |
| **PI548696** | **USA** | **V** | **375.1** | **84.09** | **0.71** | **1.5** | **226.3** | **14.08** |
| **PI556511** | **Japan** | **III** | **181.5** | **42** | **0.83** | **0.82** | **111** | **8.23** |
| **PI567387** | **China** | **IV** | **329.8** | **70.65** | **0.7** | **1.23** | **189.8** | **14.42** |
| **PI437487** | **Russia** | **III** | **211.9** | **49.16** | **0.75** | **0.91** | **115.6** | **8.88** |
| **PI533654** | **USA** | **IV** | **222.2** | **49.93** | **0.71** | **0.89** | **127.4** | **10** |
| **PI539936** | **USA** | **IV** | **324.5** | **65.96** | **0.65** | **1.08** | **200.5** | **20.2** |
| **PI540555** | **USA** | **IV** | **227.2** | **47.47** | **0.68** | **0.79** | **142.3** | **11.62** |
| **PI548158** | **USA** | **IV** | **119.4** | **22.03** | **0.6** | **0.33** | **75.8** | **8.72** |
| **PI548200** | **USA** | **IV** | **263.8** | **58.96** | **0.72** | **1.05** | **172.2** | **10.76** |
| **PI561701** | **USA** | **VI** | **226.6** | **49.88** | **0.7** | **0.88** | **124** | **10.25** |
| **PI378663** | **Russia** | **I** | **323** | **69.64** | **0.69** | **1.23** | **160.7** | **16.36** |
| **PI417550** | **Russia** | **0** | **366.9** | **70.31** | **0.6** | **1.08** | **190.7** | **23.47** |
| **PI437123** | **Russia** | **I** | **184.1** | **38.76** | **0.67** | **0.65** | **201.5** | **8.1** |
| **PI437138** | **Russia** | **0** | **392.6** | **83.07** | **0.67** | **1.4** | **224.4** | **19.69** |
| **PI437366** | **Russia** | **I** | **399.9** | **75.63** | **0.6** | **1.14** | **195.3** | **24.71** |
| **PI437476** | **Russia** | **III** | **292.5** | **65.87** | **0.73** | **1.19** | **175.8** | **13.4** |
| **PI507678** | **Russia** | **I** | **314.5** | **69.7** | **0.7** | **1.23** | **186.5** | **13.53** |
| **PI548169** | **USA** | **IV** | **437.1** | **75.32** | **0.55** | **1.03** | **292.7** | **30.44** |
| **PI548178** | **USA** | **III** | **239** | **50.52** | **0.66** | **0.85** | **130.7** | **12.36** |
| **PI548313** | **Russia** | **III** | **311.5** | **69.8** | **0.71** | **1.25** | **168** | **15.83** |
| **PI548325** | **Russia** | **0** | **361.8** | **73.32** | **0.65** | **1.19** | **244.5** | **21.15** |
| **PI548336** | **Russia** | **I** | **357.2** | **69.08** | **0.62** | **1.07** | **204.3** | **20.2** |
| **PI556637** | **USA** | **I** | **482** | **100.1** | **0.67** | **1.69** | **250** | **25.15** |
| **PI567226** | **Russia** | **0** | **316.9** | **70.32** | **0.73** | **1.27** | **189** | **16.54** |
| **PI597402** | **Russia** | **0** | **352** | **68.44** | **0.65** | **1.08** | **189** | **21.1** |
| **PI062202-2** | **China** | **IV** | **390.4** | **92.62** | **0.76** | **1.75** | **241.3** | **14.42** |
| **PI063945** | **China** | **IV** | **210.2** | **48.16** | **0.75** | **0.88** | **159.8** | **7.96** |
| **PI079616** | **China** | **III** | **209.5** | **52.45** | **0.79** | **1.05** | **145.6** | **4.6** |
| **PI084509** | **Unknown** | **III** | **307.3** | **64.44** | **0.68** | **1.08** | **181** | **16.83** |
| **PI088306** | **China** | **III** | **152.8** | **37.5** | **0.79** | **0.74** | **108.6** | **3.72** |
| **PI090723** | **Unknown** | **III** | **278.4** | **61.76** | **0.71** | **1.09** | **140.8** | **12.97** |
| **PI091731-1** | **China** | **IV** | **210.4** | **53.4** | **0.8** | **1.08** | **130.2** | **5.61** |
| **PI504495** | **Taiwan** | **V** | **333.2** | **79.91** | **0.75** | **1.54** | **196** | **12.38** |
| **PI593258** | **USA** | **III** | **206** | **51.51** | **0.8** | **1.03** | **124.3** | **6.15** |
| **PI525454** | **USA** | **IV** | **231.3** | **49.57** | **0.69** | **0.85** | **171** | **11.32** |
| **PI548193** | **USA** | **IV** | **236.2** | **61.15** | **0.82** | **1.27** | **116.8** | **8.11** |
| **PI548511** | **USA** | **II** | **190.4** | **48.45** | **0.81** | **0.98** | **111.2** | **5.14** |
| **PI548547** | **USA** | **IV** | **288.3** | **71.03** | **0.8** | **1.4** | **201.8** | **9.32** |
| **PI559932** | **USA** | **IV** | **207.6** | **49.23** | **0.76** | **0.94** | **129.5** | **9.09** |
| **PI597384** | **USA** | **IV** | **166.2** | **39.9** | **0.76** | **0.78** | **104.5** | **6.07** |
| **PI060970** | **China** | **IV** | **313.8** | **70.41** | **0.72** | **1.26** | **175.7** | **16.62** |
| **PI070242-2** | **China** | **IV** | **518.3** | **90.08** | **0.55** | **1.25** | **347.3** | **35.2** |
| **PI079797** | **China** | **III** | **408.8** | **77.61** | **0.6** | **1.18** | **230.3** | **26.83** |
| **PI088448** | **China** | **III** | **327.9** | **65.23** | **0.63** | **1.04** | **188.8** | **22.39** |
| **PI090369** | **China** | **IV** | **259.5** | **54.52** | **0.69** | **0.93** | **169.2** | **14.57** |
| **PI180501** | **Germany** | **0** | **314** | **60.53** | **0.62** | **0.93** | **185.4** | **21.91** |
| **PI360957** | **Japan** | **0** | **351** | **68.76** | **0.62** | **1.07** | **224.5** | **23.34** |
| **PI404161** | **Georgia** | **IV** | **161** | **34.44** | **0.68** | **0.59** | **121.8** | **9.1** |
| **PI416751** | **Japan** | **I** | **353** | **65.18** | **0.59** | **0.96** | **259.5** | **22.16** |
| **PI417529** | **Germany** | **0** | **314.7** | **62.66** | **0.63** | **1.01** | **205.3** | **15.66** |
| **PI438312** | **Algeria** | **III** | **362.1** | **58.04** | **0.51** | **0.74** | **237** | **26.83** |
| **PI518751** | **Former Serbia and Montenegro** | **II** | **354.7** | **73.01** | **0.65** | **1.24** | **201.1** | **19.25** |
| **PI548414** | **Japan** | **0** | **268.8** | **47.75** | **0.57** | **0.68** | **162.6** | **18.84** |
| **PI068521_1** | **China** | **II** | **183.3** | **39.88** | **0.68** | **0.69** | **133.5** | **7.99** |
| **PI068679-2** | **China** | **IV** | **227.5** | **53.92** | **0.76** | **1.03** | **248.4** | **8.2** |
| **PI081042-2** | **Japan** | **IV** | **271.7** | **61.66** | **0.7** | **1.12** | **177.5** | **13.27** |
| **PI089772** | **China** | **IV** | **257.2** | **58.02** | **0.74** | **1.05** | **200.5** | **10.6** |
| **PI153281** | **Belgium** | **0** | **329.1** | **64.52** | **0.63** | **1.01** | **217.3** | **21.39** |
| **PI209331** | **Japan** | **III** | **188.4** | **47.86** | **0.8** | **0.97** | **171.8** | **4.61** |
| **PI290136** | **France** | **0** | **271.2** | **65.21** | **0.76** | **1.25** | **148.3** | **11.55** |
| **PI438323** | **France** | **I** | **344.8** | **72.87** | **0.68** | **1.23** | **218** | **15.51** |
| **PI438335** | **Algeria** | **III** | **263** | **51.23** | **0.62** | **0.8** | **154** | **17.77** |
| **PI507467** | **Japan** | **IV** | **230** | **60.07** | **0.83** | **1.28** | **162.5** | **8.05** |
| **PI087571** | **NK** | **IV** | **237.4** | **58.34** | **0.79** | **1.15** | **104.7** | **6.96** |
| **PI087618** | **NK** | **III** | **362.2** | **91.59** | **0.81** | **1.85** | **204.5** | **10.33** |
| **PI154189** | **Netherlands** | **0** | **171** | **41.73** | **0.78** | **0.81** | **105.3** | **4.14** |
| **PI198067** | **Sweden** | **0** | **371.7** | **85.06** | **0.73** | **1.55** | **198** | **15.03** |
| **PI361093** | **Serbia** | **I** | **232.1** | **62.54** | **0.85** | **1.35** | **122.7** | **3.36** |
| **PI372418** | **Serbia** | **I** | **294.2** | **66.28** | **0.72** | **1.19** | **182.8** | **10.54** |
| **PI398614** | **SK** | **V** | **190.7** | **43.92** | **0.74** | **0.81** | **118.8** | **6.98** |
| **PI398633** | **SK** | **V** | **246.1** | **54.75** | **0.71** | **0.97** | **139.4** | **11.41** |
| **PI398965** | **SK** | **IV** | **207.6** | **49.53** | **0.76** | **0.95** | **124** | **8.85** |
| **PI408088** | **SK** | **V** | **245.7** | **62.23** | **0.8** | **1.26** | **130.2** | **6.91** |
| **PI424005** | **SK** | **III** | **216.2** | **44.16** | **0.65** | **0.72** | **117.6** | **9.55** |
| **PI430595** | **China** | **IV** | **320.9** | **74.58** | **0.73** | **1.39** | **224.8** | **13.63** |
| **PI437287** | **Moldova** | **0** | **220** | **60.19** | **0.87** | **1.31** | **95.8** | **2.4** |
| **PI437296** | **Moldova** | **0** | **262.9** | **64.94** | **0.78** | **1.28** | **101.8** | **6.58** |
| **PI567225** | **Moldova** | **0** | **264.8** | **66.1** | **0.79** | **1.33** | **142.8** | **7.03** |
| **PI597476** | **SK** | **V** | **204.3** | **42.47** | **0.66** | **0.7** | **126.5** | **11.41** |
| **PI612611** | **NK** | **III** | **381.3** | **90.19** | **0.76** | **1.7** | **212.4** | **12.83** |
| **Holladay (S-100)** | **USA** | **V** | **183.9** | **49.71** | **0.86** | **1.07** | **128** | **4.29** |
| **IA3023** | **USA** | **III** | **206.3** | **52.43** | **0.81** | **1.07** | **129.1** | **7.37** |
| **PI088788** | **China** | **III** | **161.5** | **36.19** | **0.7** | **0.65** | **114.8** | **6.44** |
| **PI090763** | **China** | **IV** | **106.7** | **25.76** | **0.77** | **0.5** | **66.75** | **3.51** |
| **PI404198B** | **China** | **IV** | **129** | **29.67** | **0.73** | **0.55** | **101.8** | **4.83** |
| **PI407729** | **China** | **IV** | **192.9** | **47.74** | **0.79** | **0.95** | **116.8** | **6.2** |
| **PI437654** | **China** | **III** | **173.3** | **39.82** | **0.74** | **0.73** | **144** | **6.66** |
| **PI437679** | **China** | **IV** | **149.6** | **38.35** | **0.82** | **0.78** | **101.4** | **3.69** |
| **PI437690** | **China** | **III** | **130.9** | **28.6** | **0.7** | **0.5** | **95** | **6.38** |
| **PI438258** | **China** | **II** | **230** | **60.75** | **0.84** | **1.28** | **148.8** | **6.73** |
| **PI467312** | **China** | **II** | **130** | **30.24** | **0.74** | **0.56** | **108.6** | **4.89** |
| **PI475783B** | **China** | **III** | **189.2** | **49.48** | **0.83** | **1.04** | **124.6** | **4.96** |
| **PI547862** | **USA** | **III** | **181.5** | **53.99** | **0.92** | **1.31** | **117** | **3.72** |
| **PI548317** | **China** | **III** | **129.4** | **32.39** | **0.8** | **0.65** | **89.4** | **3.42** |
| **PI548415** | **China** | **IV** | **141.2** | **31.29** | **0.71** | **0.55** | **102.2** | **5.64** |
| **PI567516C** | **China** | **IV** | **119.4** | **24.43** | **0.66** | **0.4** | **89.4** | **6.16** |
| **FC033243** | **Algeria** | **IV** | **147.5** | **32.41** | **0.71** | **0.57** | **98** | **7.35** |
| **PI549018** | **China** | **V** | **123.5** | **24.66** | **0.64** | **0.39** | **108** | **6.62** |
| **PI549031** | **China** | **III** | **169.3** | **42.86** | **0.81** | **0.86** | **105.4** | **5.12** |
| **PI552538** | **USA** | **III** | **188.1** | **47.86** | **0.8** | **0.98** | **100** | **4.89** |
| **PI567230** | **China** | **V** | **98.99** | **22.39** | **0.73** | **0.41** | **65.4** | **4.05** |
| **PI567336B** | **China** | **IV** | **137** | **32.86** | **0.76** | **0.63** | **103.8** | **4.98** |
| **PI567354** | **China** | **IV** | **247.6** | **69.54** | **0.9** | **1.56** | **145** | **7.3** |
| **PI567357** | **China** | **III** | **270.9** | **69.55** | **0.83** | **1.43** | **160** | **8.74** |
| **PI567383** | **China** | **V** | **181.5** | **49.42** | **0.86** | **1.08** | **129.2** | **4.45** |
| **PI567519** | **China** | **III** | **136.2** | **36.34** | **0.86** | **0.78** | **95.4** | **4.15** |
| **PI567611** | **China** | **IV** | **137.6** | **33.66** | **0.79** | **0.66** | **105.4** | **4.42** |
| **PI567690** | **China** | **III** | **125.6** | **34.39** | **0.86** | **0.75** | **89.67** | **3.15** |
| **PI567731** | **China** | **III** | **135.9** | **38.12** | **0.89** | **0.85** | **76.6** | **2.47** |
| **PI594599** | **China** | **IV** | **212.6** | **61** | **0.9** | **1.4** | **143.4** | **5.21** |
| **PI597387** | **USA** | **III** | **142.7** | **38.14** | **0.84** | **0.81** | **100.3** | **3.5** |
| **PI603176A** | **NK** | **IV** | **186.8** | **41.5** | **0.71** | **0.73** | **125.5** | **7.59** |
| **PI639740 (LD00-3309)** | **USA** | **IV** | **209** | **48.93** | **0.74** | **0.92** | **124.2** | **8.06** |
| **PI092651** | **China** | **IV** | **256** | **61.55** | **0.77** | **1.18** | **241.8** | **10.65** |
| **PI103088** | **China** | **III** | **215.4** | **57.98** | **0.85** | **1.25** | **135.4** | **7.41** |
| **PI153231** | **China** | **III** | **247** | **67.01** | **0.85** | **1.46** | **175.3** | **7.63** |
| **PI171428** | **China** | **IV** | **297** | **72.68** | **0.78** | **1.42** | **176.4** | **13.2** |
| **PI253661B** | **China** | **III** | **216.7** | **48.4** | **0.71** | **0.86** | **176.8** | **10.26** |
| **PI379618** | **China** | **V** | **251.9** | **62.54** | **0.8** | **1.24** | **166** | **8.82** |
| **PI407742** | **China** | **V** | **210.4** | **50.01** | **0.76** | **0.96** | **173** | **7.82** |
| **PI417581** | **China** | **V** | **179.2** | **42.45** | **0.75** | **0.8** | **147.7** | **4.79** |
| **PI424038B** | **China** | **V** | **123.4** | **23.14** | **0.59** | **0.35** | **109.8** | **7.8** |
| **PI437110A** | **China** | **III** | **190.3** | **46.12** | **0.76** | **0.91** | **182.8** | **8.43** |
| **PI437127A** | **China** | **IV** | **317.4** | **66.38** | **0.67** | **1.11** | **227.8** | **16.59** |
| **PI437685D** | **China** | **III** | **204.5** | **51.98** | **0.79** | **1.06** | **154.4** | **8.09** |
| **PI437776** | **China** | **III** | **346.1** | **86.12** | **0.78** | **1.73** | **226.3** | **17.42** |
| **FC029333** | **China** | **III** | **307.7** | **74.25** | **0.76** | **1.43** | **224.3** | **12.3** |
| **FC031697** | **China** | **IV** | **139.6** | **35.77** | **0.81** | **0.74** | **142.7** | **3.25** |
| **PI054615_1** | **China** | **III** | **275.3** | **68.25** | **0.8** | **1.36** | **205.8** | **11.01** |
| **PI058955** | **China** | **IV** | **147.4** | **35.33** | **0.78** | **0.68** | **135.5** | **4.84** |
| **PI068604_1** | **China** | **III** | **205.2** | **48.05** | **0.75** | **0.9** | **150.8** | **9.09** |
| **PI070466_3** | **China** | **IV** | **263.8** | **60.19** | **0.73** | **1.1** | **227.5** | **11.78** |
| **PI071465** | **China** | **V** | **228.2** | **48.72** | **0.68** | **0.83** | **128.5** | **11.62** |
| **PI083925** | **SK** | **IV** | **227.2** | **49.5** | **0.7** | **0.86** | **179** | **8.33** |
| **PI084946_2** | **China** | **IV** | **515.2** | **131.1** | **0.81** | **2.67** | **377.5** | **20.77** |
| **PI086972_2** | **China** | **IV** | **348.1** | **79.13** | **0.72** | **1.44** | **289.7** | **16.3** |
| **PI090479P** | **China** | **IV** | **174.6** | **54.13** | **0.99** | **1.37** | **113** | **5.09** |
| **PI090486** | **China** | **III** | **185.9** | **44.11** | **0.75** | **0.84** | **187.2** | **7.32** |
| **PI567426** | **China** | **IV** | **360** | **84.51** | **0.74** | **1.59** | **294.8** | **15.67** |
| **PI567558** | **China** | **III** | **314.6** | **68.81** | **0.69** | **1.2** | **263.3** | **14.8** |
| **PI578495** | **China** | **IV** | **251.6** | **61.52** | **0.77** | **1.21** | **159** | **11.01** |
| **PI603442** | **China** | **III** | **262.9** | **65.27** | **0.8** | **1.29** | **152.8** | **9.28** |
| **PI468408B** | **China** | **III** | **177.5** | **48.05** | **0.87** | **1.05** | **112.8** | **4.86** |
| **PI479735** | **France** | **III** | **181.3** | **52.55** | **0.93** | **1.22** | **121** | **3.44** |
| **PI490766** | **Georgia** | **III** | **186.1** | **51.91** | **0.89** | **1.16** | **160.3** | **3.85** |
| **PI495020** | **Germany** | **IV** | **124.1** | **31.41** | **0.79** | **0.64** | **70** | **3.79** |
| **PI561371** | **Moldova** | **IV** | **154.7** | **40.9** | **0.86** | **0.87** | **109.5** | **4.26** |
| **PI567307** | **NK** | **IV** | **126.6** | **28.73** | **0.72** | **0.52** | **126** | **5.9** |
| **PI567415A** | **Russia** | **IV** | **147** | **39.8** | **0.87** | **0.86** | **121** | **4.31** |
| **PI567416** | **Russia** | **IV** | **149.1** | **37.75** | **0.8** | **0.76** | **102.8** | **4.93** |
| **PI567428** | **Russia** | **IV** | **146.7** | **36.65** | **0.79** | **0.73** | **113.4** | **4.79** |
| **PI567435B** | **Russia** | **III** | **167.7** | **41.2** | **0.76** | **0.82** | **127.6** | **6.27** |
| **PI567439** | **Russia** | **V** | **132.5** | **35.89** | **0.86** | **0.77** | **107.8** | **2.78** |
| **PI567488A** | **Russia** | **IV** | **193** | **51.44** | **0.86** | **1.1** | **110.8** | **5.17** |
| **PI567532** | **Russia** | **IV** | **149.4** | **37.39** | **0.8** | **0.75** | **121.4** | **4.94** |
| **PI567548** | **Russia** | **IV** | **134.7** | **32.5** | **0.76** | **0.63** | **89.83** | **5.93** |
| **PI567576** | **Russia** | **III** | **180.9** | **48.01** | **0.85** | **1.02** | **127.3** | **6.68** |
| **PI567780B** | **China** | **IV** | **143.8** | **33.11** | **0.73** | **0.61** | **113.4** | **5.67** |
| **PI538386A** | **Japan** | **III** | **209** | **45.25** | **0.7** | **0.78** | **125.3** | **11.75** |
| **PI548383** | **Japan** | **III** | **146.1** | **33.77** | **0.74** | **0.63** | **108.8** | **6.57** |
| **PI548400** | **Japan** | **IV** | **158.2** | **40.1** | **0.81** | **0.82** | **101.8** | **4.96** |
| **PI567346** | **NK** | **V** | **191.6** | **44.65** | **0.75** | **0.83** | **142.3** | **6.17** |
| **PI567352A** | **Peru** | **IV** | **260.9** | **80.26** | **0.98** | **1.97** | **132** | **6.11** |
| **PI567353** | **Philippines** | **IV** | **250.8** | **58.79** | **0.74** | **1.1** | **213.7** | **10.36** |
| **PI567361** | **Philippines** | **III** | **452.9** | **96.06** | **0.68** | **1.64** | **284.7** | **23.24** |
| **PI567407** | **Romania** | **V** | **294.8** | **66.44** | **0.72** | **1.2** | **167.5** | **15.58** |
| **PI567408** | **Russia** | **V** | **209.9** | **57.72** | **0.88** | **1.27** | **133.4** | **5.01** |
| **PI567675** | **Russia** | **IV** | **175.6** | **42.76** | **0.76** | **0.85** | **108.6** | **6.64** |
| **PI567685** | **Russia** | **IV** | **207** | **49.94** | **0.78** | **0.96** | **160** | **7.5** |
| **PI567698A** | **Russia** | **IV** | **170.7** | **42.29** | **0.79** | **0.84** | **118.2** | **5.74** |
| **PI567726** | **Russia** | **IV** | **219.7** | **45.96** | **0.66** | **0.77** | **156.2** | **12.8** |
| **PI567746** | **Russia** | **IV** | **157.5** | **40.94** | **0.8** | **0.86** | **94.8** | **5.45** |
| **PI574477** | **SK** | **IV** | **206.1** | **49.5** | **0.75** | **0.95** | **152** | **7.97** |
| **PI587588B** | **SK** | **V** | **235.6** | **51.85** | **0.71** | **0.91** | **149.8** | **11.48** |
| **PI587712B** | **SK** | **V** | **209.5** | **51.71** | **0.79** | **1.02** | **158.2** | **7.24** |
| **PI068423** | **China** | **III** | **175.4** | **43.14** | **0.78** | **0.85** | **94.5** | **5.13** |
| **PI068523** | **China** | **III** | **295.2** | **71.75** | **0.75** | **1.4** | **182.5** | **10.53** |
| **PI068732_1** | **China** | **II** | **174.4** | **43.4** | **0.79** | **0.86** | **145** | **6.28** |
| **PI297505** | **China** | **I** | **156** | **35** | **0.72** | **0.63** | **117.7** | **6.02** |
| **PI417381** | **Japan** | **0** | **193** | **45** | **0.74** | **0.84** | **116.5** | **7.52** |
| **PI603495B** | **China** | **V** | **247.2** | **65.91** | **0.85** | **1.4** | **145.5** | **8.04** |
| **PI603526** | **China** | **IV** | **248.7** | **64.75** | **0.84** | **1.35** | **124.2** | **6.73** |
| **PI603549** | **China** | **III** | **223.9** | **53.67** | **0.76** | **1.03** | **140** | **7.77** |
| **PI603556** | **China** | **III** | **202** | **49.1** | **0.77** | **0.95** | **119.5** | **5.23** |
| **PI070208** | **China** | **IV** | **165.6** | **35.98** | **0.69** | **0.62** | **125.8** | **5.38** |
| **PI079870-4** | **China** | **IV** | **86.71** | **20.12** | **0.72** | **0.37** | **61.6** | **2.67** |
| **PI430598B** | **China** | **IV** | **143.5** | **35.78** | **0.8** | **0.71** | **82.8** | **4.42** |
| **PI594456A** | **China** | **III** | **116.3** | **24.54** | **0.67** | **0.42** | **89.4** | **5.45** |
| **PI594880** | **China** | **V** | **96.81** | **22.18** | **0.73** | **0.41** | **72** | **2.94** |
| **PI594922** | **USA** | **V** | **172.7** | **42.02** | **0.77** | **0.82** | **90.67** | **4.42** |
| **PI597478B** | **SK** | **III** | **138.4** | **30.58** | **0.7** | **0.54** | **81.75** | **4.11** |
| **PI598358** | **Unknown** | **V** | **100.3** | **21.9** | **0.7** | **0.38** | **68.8** | **4.6** |
| **PI606374** | **Vietnam** | **III** | **71.41** | **14.23** | **0.63** | **0.23** | **50.33** | **3.85** |
| **PI632418** | **USA** | **V** | **106** | **25.39** | **0.76** | **0.48** | **81** | **2.76** |
| **PI093055S** | **China** | **V** | **239.4** | **52.63** | **0.71** | **0.94** | **150.3** | **10.75** |
| **PI093563** | **China** | **III** | **388.2** | **96.92** | **0.79** | **1.93** | **205.3** | **16.93** |
| **PI103079** | **China** | **V** | **160** | **39.38** | **0.77** | **0.79** | **100** | **6.88** |
| **PI123587** | **Belgium** | **V** | **218.5** | **51.73** | **0.77** | **1** | **124** | **9.98** |
| **PI235347** | **Japan** | **IV** | **253** | **49.8** | **0.65** | **0.81** | **153.4** | **17.98** |
| **PI253656B** | **China** | **IV** | **215.1** | **54.11** | **0.8** | **1.09** | **139.8** | **8.18** |
| **PI378682C** | **Japan** | **IV** | **286.4** | **65.91** | **0.73** | **1.21** | **137** | **11.75** |
| **PI603494** | **China** | **IV** | **121.2** | **26.39** | **0.7** | **0.46** | **79.86** | **5.59** |
| **4J105-3-4** | **USA** | **III** | **165.5** | **41.11** | **0.79** | **0.81** | **98.33** | **5.5** |
| **5M20-2-5-2** | **USA** | **III** | **279.9** | **73.23** | **0.84** | **1.53** | **149** | **10.32** |
| **CL0J095-4-6** | **USA** | **III** | **182.2** | **36.7** | **0.64** | **0.59** | **128.8** | **10.87** |
| **LD02-9050** | **USA** | **IV** | **128.6** | **30.73** | **0.76** | **0.59** | **83.4** | **4.45** |
| **LG00-3372** | **USA** | **III** | **169.8** | **42.34** | **0.78** | **0.85** | **88.6** | **5.87** |
| **LG03-2979** | **USA** | **III** | **223.7** | **57.74** | **0.83** | **1.2** | **114.2** | **8.5** |
| **LG03-3191** | **USA** | **IV** | **168.4** | **44.33** | **0.83** | **0.95** | **108** | **7.02** |
| **LG04-4717** | **USA** | **III** | **158.4** | **39.24** | **0.79** | **0.78** | **108** | **4.09** |
| **LG05-4292** | **USA** | **IV** | **166.5** | **42.09** | **0.82** | **0.85** | **99.5** | **6.81** |
| **LG05-4317** | **USA** | **IV** | **233.4** | **58.75** | **0.8** | **1.18** | **122.3** | **7** |
| **LG05-4464** | **USA** | **III** | **297.4** | **74.61** | **0.8** | **1.5** | **110.8** | **9.91** |
| **LG05-4832** | **USA** | **III** | **267.6** | **64.95** | **0.78** | **1.26** | **161.4** | **10.5** |
| **LG90-2550** | **USA** | **III** | **163.3** | **33.48** | **0.65** | **0.55** | **100** | **7.38** |
| **LG94-1906** | **USA** | **II** | **88.8** | **21.45** | **0.73** | **0.42** | **51.5** | **4.11** |
| **LG98-1605** | **USA** | **III** | **252.8** | **59.15** | **0.8** | **1.12** | **177.8** | **9.83** |
| **NE3001** | **USA** | **III** | **333** | **82.62** | **0.78** | **1.63** | **154** | **12.93** |
| **PI404188A** | **China** | **II** | **231.6** | **59.51** | **0.84** | **1.23** | **156.3** | **6.13** |
| **Prohio** | **USA** | **III** | **365.2** | **83.84** | **0.73** | **1.54** | **173.6** | **12.91** |
| **S06-13640** | **USA** | **IV** | **89.41** | **19.76** | **0.69** | **0.35** | **55.5** | **3.51** |
| **TN05-3027** | **USA** | **V** | **257.7** | **65.89** | **0.81** | **1.36** | **136.2** | **8.03** |
| **U03-100612** | **USA** | **I** | **357.6** | **94.22** | **0.84** | **1.98** | **147.3** | **12.88** |
| **PI200503** | **Japan** | **V** | **155** | **32.78** | **0.67** | **0.55** | **80.25** | **6.81** |
| **PI209334** | **Japan** | **III** | **206.4** | **55.18** | **0.86** | **1.18** | **105** | **7.58** |
| **PI342434** | **Japan** | **V** | **191.7** | **37.46** | **0.62** | **0.59** | **155.6** | **13.07** |
| **PI377574** | **Japan** | **IV** | **148** | **41.33** | **0.87** | **0.93** | **65.5** | **4.26** |
| **PI416838** | **Japan** | **V** | **409.2** | **95.79** | **0.74** | **1.79** | **226** | **18.38** |
| **PI417007** | **Japan** | **IV** | **261.4** | **46.88** | **0.57** | **0.67** | **176.2** | **18.4** |
| **PI417015** | **Japan** | **III** | **385.1** | **79.84** | **0.64** | **1.34** | **194.3** | **22.85** |
| **PI417077** | **Japan** | **III** | **262.2** | **59.42** | **0.72** | **1.07** | **141.5** | **12.12** |
| **PI417479** | **Japan** | **IV** | **320.9** | **75.92** | **0.76** | **1.45** | **164.5** | **17.15** |
| **PI504288** | **Japan** | **V** | **85.98** | **16.16** | **0.6** | **0.24** | **65.5** | **4.51** |
| **PI506933** | **Japan** | **IV** | **184.9** | **42.54** | **0.73** | **0.79** | **103** | **8.7** |
| **PI548364** | **Japan** | **IV** | **258** | **63.12** | **0.79** | **1.25** | **166.8** | **10.37** |
| **PI561387** | **Japan** | **V** | **90.63** | **19.02** | **0.67** | **0.32** | **65.33** | **4.3** |
| **PI080837** | **Japan** | **IV** | **213.1** | **43.89** | **0.65** | **0.72** | **142** | **10.02** |
| **PI081041** | **Japan** | **III** | **382.7** | **79.47** | **0.68** | **1.33** | **225** | **19.6** |
| **PI081785** | **Japan** | **III** | **370.2** | **68.14** | **0.58** | **1** | **189.8** | **26.14** |
| **PI084631** | **SK** | **III** | **402.4** | **95.6** | **0.75** | **1.84** | **278** | **17.47** |
| **PI084973** | **Japan** | **III** | **458.7** | **107.4** | **0.74** | **2** | **298.3** | **20.73** |
| **PI086084** | **Japan** | **V** | **236.8** | **53.53** | **0.73** | **0.97** | **148.8** | **10.51** |
| **PI086982** | **SK** | **V** | **178.3** | **42.69** | **0.76** | **0.82** | **111.3** | **6.78** |
| **PI091083** | **SK** | **III** | **285.2** | **70.17** | **0.79** | **1.38** | **178.8** | **10.07** |
| **Line/Accession** | **Country of origin** | **Maturity group** | **TSA3_8DAT** | **TRW (cm)_8DAT** | **TCA (cm2)_8DAT** | **RDI_8DAT** | **SOL_8DAT** | **COPM_8DAT** |
| **Lee** | **USA** | **VI** | **6.34** | **17.45** | **176.9** | **0.67** | **0.1** | **6.47** |
| **Magellan** | **USA** | **IV** | **11.49** | **16.67** | **185.3** | **0.43** | **0.13** | **6.05** |
| **Maverick** | **USA** | **III** | **8.96** | **15.16** | **146.3** | **0.47** | **0.12** | **5.04** |
| **PI091725** | **NK** | **V** | **10.98** | **18.45** | **237.8** | **0.48** | **0.11** | **6.18** |
| **PI398595** | **SK** | **V** | **6.37** | **16.51** | **192.3** | **0.53** | **0.09** | **6.24** |
| **PI408105A** | **SK** | **IV** | **15.36** | **23.21** | **298.8** | **0.61** | **0.12** | **7.35** |
| **PI417242** | **China** | **II** | **14.76** | **20.91** | **255.2** | **0.61** | **0.09** | **6.95** |
| **PI438500** | **USA** | **III** | **15.63** | **26.61** | **335.8** | **0.6** | **0.12** | **7.61** |
| **PI467347** | **China** | **II** | **3.81** | **11.34** | **129.5** | **0.52** | **0.09** | **5.22** |
| **PI518668** | **USA** | **IV** | **12.17** | **22.19** | **262.9** | **0.56** | **0.1** | **6.82** |
| **PI548316** | **China** | **III** | **9.52** | **16.93** | **194.3** | **0.57** | **0.13** | **6.53** |
| **PI561271** | **China** | **V** | **7.23** | **18.97** | **284.8** | **0.55** | **0.09** | **7.89** |
| **PI567305** | **China** | **IV** | **8.31** | **14.52** | **196.6** | **0.48** | **0.09** | **6.13** |
| **PI567343** | **China** | **V** | **8.21** | **13.76** | **156.8** | **0.54** | **0.13** | **6.67** |
| **PI567651** | **China** | **IV** | **8.99** | **20.23** | **213** | **0.6** | **0.1** | **5.88** |
| **PI092728** | **China** | **III** | **8.34** | **16.51** | **214.8** | **0.52** | **0.1** | **6.47** |
| **PI518671 (Williams 82)** | **USA** | **III** | **6.44** | **16.07** | **184.6** | **0.59** | **0.11** | **6.31** |
| **PI087617** | **NK** | **III** | **22.93** | **26.6** | **462.5** | **0.48** | **0.11** | **9.06** |
| **PI196175** | **SK** | **V** | **10.66** | **18.19** | **254.7** | **0.56** | **0.1** | **7.74** |
| **PI209332** | **Japan** | **IV** | **3.02** | **11.6** | **143.7** | **0.3** | **0.07** | **6.27** |
| **PI248515** | **Japan** | **IV** | **4.61** | **12.21** | **169.9** | **0.52** | **0.08** | **7.61** |
| **PI398593** | **SK** | **V** | **9.19** | **19.92** | **367.9** | **0.67** | **0.09** | **10.83** |
| **PI398610** | **SK** | **V** | **5.68** | **15.57** | **201.1** | **0.37** | **0.08** | **6.01** |
| **PI407788A** | **SK** | **IV** | **12.73** | **20.78** | **346.3** | **0.41** | **0.11** | **7.49** |
| **PI407965** | **SK** | **V** | **14.2** | **21.78** | **401.1** | **0.59** | **0.09** | **9.71** |
| **PI424608A** | **SK** | **IV** | **20.99** | **28.02** | **434.9** | **0.67** | **0.11** | **10.19** |
| **PI458515** | **China** | **IV** | **12.29** | **22.15** | **322.4** | **0.42** | **0.09** | **6.8** |
| **PI495017C** | **China** | **IV** | **13.23** | **12.64** | **246.7** | **0.44** | **0.09** | **8.86** |
| **PI603154** | **NK** | **V** | **10.83** | **14.92** | **234.8** | **0.42** | **0.1** | **7.45** |
| **PI603175** | **NK** | **IV** | **9.49** | **19.33** | **260.2** | **0.57** | **0.1** | **7.76** |
| **PI605869A** | **Vietnam** | **V** | **9.95** | **22.93** | **333.4** | **0.59** | **0.11** | **8.14** |
| **PI404166** | **China** | **III** | **4.17** | **15.91** | **264.7** | **0.36** | **0.07** | **7.49** |
| **PI417091** | **Japan** | **II** | **5.84** | **17.88** | **268.1** | **0.52** | **0.08** | **8.21** |
| **PI437169B** | **Russia** | **II** | **11.48** | **19.36** | **253** | **0.37** | **0.11** | **6.23** |
| **PI437655** | **China** | **III** | **10.51** | **19.58** | **279.9** | **0.33** | **0.07** | **6.99** |
| **PI437725** | **China** | **IV** | **5.06** | **16.14** | **233** | **0.45** | **0.08** | **6.94** |
| **PI468915** | **China** | **II** | **6.43** | **20.9** | **283.2** | **0.47** | **0.07** | **7.4** |
| **PI548359** | **China** | **IV** | **10.12** | **16.31** | **226.5** | **0.26** | **0.08** | **5.83** |
| **PI548402 (peking)** | **China** | **IV** | **6.83** | **13.93** | **137.5** | **0.52** | **0.12** | **5.3** |
| **PI548427** | **China** | **IV** | **10.83** | **18.81** | **232.7** | **0.51** | **0.12** | **6.31** |
| **PI548619** | **USA** | **IV** | **7.06** | **19.62** | **309** | **0.44** | **0.07** | **7.06** |
| **PI548633** | **Japan** | **IV** | **6.72** | **9.35** | **105.4** | **0.39** | **0.1** | **5.41** |
| **PI548696** | **USA** | **V** | **10.73** | **18.58** | **293.2** | **0.54** | **0.09** | **7.9** |
| **PI556511** | **Japan** | **III** | **7.39** | **14.48** | **146.1** | **0.35** | **0.1** | **4.81** |
| **PI567387** | **China** | **IV** | **7.22** | **18.65** | **234.3** | **0.54** | **0.11** | **6.49** |
| **PI437487** | **Russia** | **III** | **6.14** | **13.05** | **123.6** | **0.64** | **0.13** | **5.42** |
| **PI533654** | **USA** | **IV** | **5.74** | **14.56** | **141.2** | **0.52** | **0.11** | **5.26** |
| **PI539936** | **USA** | **IV** | **6.43** | **15.43** | **180.2** | **0.45** | **0.12** | **6.61** |
| **PI540555** | **USA** | **IV** | **5.08** | **16.33** | **175.7** | **0.52** | **0.09** | **5.89** |
| **PI548158** | **USA** | **IV** | **1.34** | **9.8** | **83.43** | **0.54** | **0.08** | **4.66** |
| **PI548200** | **USA** | **IV** | **7.23** | **14.65** | **210.4** | **0.6** | **0.09** | **8.1** |
| **PI561701** | **USA** | **VI** | **5.37** | **14.87** | **164.6** | **0.6** | **0.1** | **6.84** |
| **PI378663** | **Russia** | **I** | **9.76** | **21.54** | **257.8** | **0.44** | **0.09** | **5.7** |
| **PI417550** | **Russia** | **0** | **6.44** | **16.35** | **198.7** | **0.57** | **0.11** | **6.65** |
| **PI437123** | **Russia** | **I** | **3.25** | **11.62** | **163.7** | **0.48** | **0.08** | **6.72** |
| **PI437138** | **Russia** | **0** | **9.71** | **19.68** | **193.7** | **0.66** | **0.14** | **6.03** |
| **PI437366** | **Russia** | **I** | **7.81** | **23.54** | **290.4** | **0.42** | **0.08** | **6.54** |
| **PI437476** | **Russia** | **III** | **8.35** | **17.51** | **162.4** | **0.6** | **0.13** | **5.44** |
| **PI507678** | **Russia** | **I** | **6.7** | **21.15** | **193.8** | **0.67** | **0.12** | **6.75** |
| **PI548169** | **USA** | **IV** | **5.7** | **26.61** | **411.7** | **0.32** | **0.06** | **6.81** |
| **PI548178** | **USA** | **III** | **4.91** | **14.7** | **163.6** | **0.46** | **0.1** | **5.68** |
| **PI548313** | **Russia** | **III** | **9.79** | **15.65** | **151.4** | **0.56** | **0.15** | **5.07** |
| **PI548325** | **Russia** | **0** | **7.1** | **16.71** | **212.7** | **0.46** | **0.12** | **6.72** |
| **PI548336** | **Russia** | **I** | **6.79** | **18.33** | **212** | **0.47** | **0.11** | **5.88** |
| **PI556637** | **USA** | **I** | **10.05** | **23.26** | **299.3** | **0.58** | **0.11** | **7.46** |
| **PI567226** | **Russia** | **0** | **8.07** | **18.67** | **178.3** | **0.57** | **0.14** | **5.44** |
| **PI597402** | **Russia** | **0** | **6.61** | **19.44** | **246.7** | **0.36** | **0.1** | **5.78** |
| **PI062202-2** | **China** | **IV** | **12.61** | **15.88** | **196.2** | **0.63** | **0.17** | **7.41** |
| **PI063945** | **China** | **IV** | **5.34** | **11.46** | **128.9** | **0.61** | **0.13** | **6** |
| **PI079616** | **China** | **III** | **6.54** | **13.92** | **138.7** | **0.49** | **0.12** | **5.06** |
| **PI084509** | **Unknown** | **III** | **5.66** | **10.62** | **133** | **0.59** | **0.16** | **5.96** |
| **PI088306** | **China** | **III** | **4.32** | **10.5** | **106.7** | **0.48** | **0.12** | **5.29** |
| **PI090723** | **Unknown** | **III** | **7.36** | **12.01** | **129** | **0.44** | **0.15** | **5.25** |
| **PI091731-1** | **China** | **IV** | **8.8** | **15.35** | **180.7** | **0.3** | **0.1** | **5.15** |
| **PI504495** | **Taiwan** | **V** | **10.02** | **16.49** | **191.1** | **0.36** | **0.14** | **5.88** |
| **PI593258** | **USA** | **III** | **8.38** | **14.65** | **127.6** | **0.54** | **0.14** | **4.87** |
| **PI525454** | **USA** | **IV** | **6.98** | **21.21** | **213.8** | **0.57** | **0.08** | **5.91** |
| **PI548193** | **USA** | **IV** | **9.24** | **14.33** | **163.3** | **0.54** | **0.13** | **5.34** |
| **PI548511** | **USA** | **II** | **6.37** | **11.95** | **134.3** | **0.6** | **0.12** | **6.38** |
| **PI548547** | **USA** | **IV** | **10.8** | **16.27** | **170.6** | **0.55** | **0.13** | **5.57** |
| **PI559932** | **USA** | **IV** | **6.65** | **11.9** | **108.7** | **0.58** | **0.15** | **4.93** |
| **PI597384** | **USA** | **IV** | **4.65** | **10.84** | **107.3** | **0.62** | **0.12** | **5.49** |
| **PI060970** | **China** | **IV** | **9.17** | **18.18** | **227.5** | **0.59** | **0.1** | **8** |
| **PI070242-2** | **China** | **IV** | **7.54** | **28.2** | **397.7** | **0.51** | **0.07** | **7.43** |
| **PI079797** | **China** | **III** | **7.28** | **13.98** | **197.1** | **0.58** | **0.12** | **7.39** |
| **PI088448** | **China** | **III** | **7.14** | **13.82** | **182.8** | **0.54** | **0.12** | **7.57** |
| **PI090369** | **China** | **IV** | **6.17** | **14.07** | **158.3** | **0.6** | **0.12** | **6.78** |
| **PI180501** | **Germany** | **0** | **6.64** | **11.32** | **151.2** | **0.61** | **0.13** | **7.27** |
| **PI360957** | **Japan** | **0** | **8.24** | **12.73** | **200.4** | **0.56** | **0.12** | **7.46** |
| **PI404161** | **Georgia** | **IV** | **3.6** | **10.96** | **111.8** | **0.51** | **0.11** | **6.01** |
| **PI416751** | **Japan** | **I** | **5.53** | **16.31** | **219.7** | **0.6** | **0.1** | **7** |
| **PI417529** | **Germany** | **0** | **5.68** | **18.67** | **225.9** | **0.59** | **0.09** | **6.17** |
| **PI438312** | **Algeria** | **III** | **4.94** | **21.4** | **241.2** | **0.63** | **0.08** | **7.72** |
| **PI518751** | **Former Serbia and Montenegro** | **II** | **8.64** | **17.25** | **221.5** | **0.45** | **0.11** | **6.87** |
| **PI548414** | **Japan** | **0** | **4.16** | **14.19** | **159.4** | **0.49** | **0.1** | **6.22** |
| **PI068521_1** | **China** | **II** | **5.02** | **13.13** | **153.5** | **0.44** | **0.08** | **6.05** |
| **PI068679-2** | **China** | **IV** | **8.67** | **11.26** | **170.3** | **0.63** | **0.11** | **8.53** |
| **PI081042-2** | **Japan** | **IV** | **8.37** | **9.08** | **126.3** | **0.73** | **0.15** | **9.72** |
| **PI089772** | **China** | **IV** | **7.76** | **13.97** | **167.3** | **0.55** | **0.12** | **6.87** |
| **PI153281** | **Belgium** | **0** | **5.92** | **18.05** | **221.9** | **0.58** | **0.09** | **7.54** |
| **PI209331** | **Japan** | **III** | **9.33** | **6.58** | **92.52** | **0.58** | **0.16** | **7.19** |
| **PI290136** | **France** | **0** | **11.9** | **9.93** | **161.1** | **0.5** | **0.14** | **8.3** |
| **PI438323** | **France** | **I** | **7.3** | **14.61** | **260.5** | **0.57** | **0.09** | **9.01** |
| **PI438335** | **Algeria** | **III** | **5.5** | **12.95** | **186.9** | **0.56** | **0.09** | **7.94** |
| **PI507467** | **Japan** | **IV** | **10.86** | **10.85** | **134.7** | **0.55** | **0.14** | **6.8** |
| **PI087571** | **NK** | **IV** | **10.83** | **9.49** | **106.8** | **0.61** | **0.18** | **6.54** |
| **PI087618** | **NK** | **III** | **15.83** | **14.57** | **196.1** | **0.62** | **0.15** | **7.09** |
| **PI154189** | **Netherlands** | **0** | **7.63** | **10.71** | **119.9** | **0.64** | **0.11** | **7.35** |
| **PI198067** | **Sweden** | **0** | **11.08** | **15.18** | **188.2** | **0.7** | **0.15** | **7.93** |
| **PI361093** | **Serbia** | **I** | **12.04** | **10.24** | **115.7** | **0.63** | **0.17** | **6.97** |
| **PI372418** | **Serbia** | **I** | **7.95** | **13.88** | **173.1** | **0.63** | **0.12** | **7.12** |
| **PI398614** | **SK** | **V** | **5.66** | **11.11** | **116** | **0.54** | **0.14** | **5.51** |
| **PI398633** | **SK** | **V** | **7.3** | **12.25** | **155** | **0.46** | **0.12** | **6.26** |
| **PI398965** | **SK** | **IV** | **7.73** | **9.86** | **105.5** | **0.52** | **0.15** | **4.86** |
| **PI408088** | **SK** | **V** | **9.61** | **12.9** | **147.7** | **0.52** | **0.13** | **6.33** |
| **PI424005** | **SK** | **III** | **3.83** | **12.06** | **152.3** | **0.45** | **0.1** | **6.43** |
| **PI430595** | **China** | **IV** | **9.4** | **16.11** | **169.9** | **0.65** | **0.14** | **5.86** |
| **PI437287** | **Moldova** | **0** | **11.88** | **13.4** | **171.5** | **0.7** | **0.11** | **8.6** |
| **PI437296** | **Moldova** | **0** | **9.93** | **11.02** | **127.9** | **0.68** | **0.16** | **7.16** |
| **PI567225** | **Moldova** | **0** | **12.19** | **12.34** | **156.4** | **0.54** | **0.13** | **6.63** |
| **PI597476** | **SK** | **V** | **4.65** | **10.92** | **119.9** | **0.52** | **0.11** | **5.54** |
| **PI612611** | **NK** | **III** | **14.06** | **18.6** | **239.2** | **0.66** | **0.13** | **8.37** |
| **Holladay (S-100)** | **USA** | **V** | **9.54** | **11.2** | **103** | **0.56** | **0.15** | **5.12** |
| **IA3023** | **USA** | **III** | **7.72** | **15.14** | **125.9** | **0.6** | **0.14** | **4.63** |
| **PI088788** | **China** | **III** | **3.85** | **10.4** | **97.56** | **0.55** | **0.11** | **5.28** |
| **PI090763** | **China** | **IV** | **3.7** | **8.71** | **64.68** | **0.59** | **0.13** | **4.8** |
| **PI404198B** | **China** | **IV** | **3.43** | **10.8** | **94.31** | **0.3** | **0.1** | **4.16** |
| **PI407729** | **China** | **IV** | **7.21** | **12.09** | **121.6** | **0.45** | **0.12** | **5.35** |
| **PI437654** | **China** | **III** | **4.3** | **13.32** | **124.7** | **0.48** | **0.1** | **5.31** |
| **PI437679** | **China** | **IV** | **5.9** | **10.34** | **96.78** | **0.67** | **0.13** | **6.33** |
| **PI437690** | **China** | **III** | **2.49** | **10.11** | **90.76** | **0.49** | **0.1** | **5.03** |
| **PI438258** | **China** | **II** | **9.64** | **15.74** | **128.2** | **0.49** | **0.15** | **4.53** |
| **PI467312** | **China** | **II** | **3.15** | **9.72** | **97.16** | **0.57** | **0.1** | **5.53** |
| **PI475783B** | **China** | **III** | **7.87** | **12.3** | **122.4** | **0.43** | **0.13** | **5.1** |
| **PI547862** | **USA** | **III** | **11.54** | **11.63** | **116.2** | **0.45** | **0.15** | **4.92** |
| **PI548317** | **China** | **III** | **4.87** | **9.44** | **85.47** | **0.45** | **0.12** | **4.55** |
| **PI548415** | **China** | **IV** | **3.03** | **10.32** | **90.88** | **0.53** | **0.11** | **5.08** |
| **PI567516C** | **China** | **IV** | **2.51** | **10.12** | **90.04** | **0.46** | **0.09** | **4.56** |
| **FC033243** | **Algeria** | **IV** | **3.76** | **10.35** | **88.66** | **0.61** | **0.12** | **5.07** |
| **PI549018** | **China** | **V** | **2.28** | **10.02** | **81.8** | **0.45** | **0.09** | **4.23** |
| **PI549031** | **China** | **III** | **5.93** | **13.15** | **135.5** | **0.27** | **0.1** | **4.99** |
| **PI552538** | **USA** | **III** | **8.51** | **14** | **122.1** | **0.55** | **0.12** | **5.24** |
| **PI567230** | **China** | **V** | **2.33** | **8.87** | **78.79** | **0.42** | **0.09** | **4.54** |
| **PI567336B** | **China** | **IV** | **3.52** | **9.27** | **87.45** | **0.27** | **0.12** | **4.19** |
| **PI567354** | **China** | **IV** | **11.75** | **14.52** | **137.1** | **0.37** | **0.16** | **4.85** |
| **PI567357** | **China** | **III** | **12.59** | **16.8** | **155.1** | **0.53** | **0.15** | **5.11** |
| **PI567383** | **China** | **V** | **10.52** | **14.4** | **130.8** | **0.48** | **0.13** | **4.85** |
| **PI567519** | **China** | **III** | **6.17** | **11.7** | **90.86** | **0.48** | **0.12** | **4.64** |
| **PI567611** | **China** | **IV** | **2.88** | **10.76** | **113.7** | **0.61** | **0.1** | **5.98** |
| **PI567690** | **China** | **III** | **4.28** | **9.3** | **103.6** | **0.34** | **0.1** | **4.78** |
| **PI567731** | **China** | **III** | **8.41** | **13.47** | **111.2** | **0.56** | **0.11** | **4.9** |
| **PI594599** | **China** | **IV** | **12.86** | **14.57** | **136.6** | **0.33** | **0.14** | **4.75** |
| **PI597387** | **USA** | **III** | **8.09** | **12.87** | **122.5** | **0.42** | **0.1** | **4.53** |
| **PI603176A** | **NK** | **IV** | **4.9** | **12.17** | **98.86** | **0.64** | **0.14** | **4.75** |
| **PI639740 (LD00-3309)** | **USA** | **IV** | **7.38** | **14.53** | **139.3** | **0.41** | **0.11** | **4.71** |
| **PI092651** | **China** | **IV** | **9.68** | **20.13** | **156.7** | **0.58** | **0.13** | **5.06** |
| **PI103088** | **China** | **III** | **8.83** | **13.31** | **115.5** | **0.55** | **0.16** | **4.56** |
| **PI153231** | **China** | **III** | **10.9** | **16.25** | **143.8** | **0.47** | **0.14** | **4.61** |
| **PI171428** | **China** | **IV** | **9.64** | **17.4** | **142.9** | **0.64** | **0.16** | **5.1** |
| **PI253661B** | **China** | **III** | **6.08** | **12.64** | **122.8** | **0.5** | **0.13** | **4.82** |
| **PI379618** | **China** | **V** | **8.55** | **15.62** | **137.7** | **0.72** | **0.14** | **5.39** |
| **PI407742** | **China** | **V** | **6.16** | **14.29** | **144.1** | **0.47** | **0.12** | **4.81** |
| **PI417581** | **China** | **V** | **6.9** | **12.92** | **110.9** | **0.8** | **0.12** | **5.47** |
| **PI424038B** | **China** | **V** | **1.83** | **11.05** | **108** | **0.39** | **0.07** | **4.71** |
| **PI437110A** | **China** | **III** | **5.37** | **13.78** | **96.21** | **0.7** | **0.15** | **4.7** |
| **PI437127A** | **China** | **IV** | **8.23** | **16.78** | **151.4** | **0.65** | **0.14** | **5.27** |
| **PI437685D** | **China** | **III** | **8.66** | **13.07** | **115.3** | **0.57** | **0.14** | **4.87** |
| **PI437776** | **China** | **III** | **12.18** | **14.9** | **176** | **0.51** | **0.16** | **5.43** |
| **FC029333** | **China** | **III** | **11.16** | **20.11** | **161.9** | **0.65** | **0.15** | **5.21** |
| **FC031697** | **China** | **IV** | **5.58** | **13.86** | **131.2** | **0.47** | **0.09** | **4.46** |
| **PI054615_1** | **China** | **III** | **11.15** | **15.78** | **147.1** | **0.78** | **0.16** | **5.82** |
| **PI058955** | **China** | **IV** | **3.42** | **14.87** | **106.8** | **0.64** | **0.11** | **4.82** |
| **PI068604_1** | **China** | **III** | **6.16** | **13.14** | **116** | **0.47** | **0.14** | **4.88** |
| **PI070466_3** | **China** | **IV** | **8.01** | **16.72** | **155.2** | **0.57** | **0.12** | **5.37** |
| **PI071465** | **China** | **V** | **5.36** | **13.35** | **137.4** | **0.49** | **0.12** | **5.26** |
| **PI083925** | **SK** | **IV** | **4.82** | **16.25** | **149.6** | **0.53** | **0.1** | **4.79** |
| **PI084946_2** | **China** | **IV** | **19.47** | **24.1** | **237** | **0.75** | **0.19** | **6.68** |
| **PI086972_2** | **China** | **IV** | **9.91** | **17.52** | **201.4** | **0.41** | **0.13** | **5.4** |
| **PI090479P** | **China** | **IV** | **9.41** | **12.83** | **116.6** | **0.51** | **0.15** | **4.92** |
| **PI090486** | **China** | **III** | **5.64** | **13.17** | **107.6** | **0.49** | **0.13** | **4.6** |
| **PI567426** | **China** | **IV** | **10.21** | **18.71** | **199.9** | **0.53** | **0.14** | **6.11** |
| **PI567558** | **China** | **III** | **7.29** | **16.63** | **171.5** | **0.57** | **0.13** | **5.85** |
| **PI578495** | **China** | **IV** | **9.56** | **13.57** | **132.7** | **0.52** | **0.15** | **5.12** |
| **PI603442** | **China** | **III** | **10.2** | **14.87** | **124.9** | **0.57** | **0.17** | **4.73** |
| **PI468408B** | **China** | **III** | **9.29** | **11.73** | **100.5** | **0.4** | **0.16** | **3.93** |
| **PI479735** | **France** | **III** | **11.5** | **10.73** | **118.8** | **0.23** | **0.14** | **4.91** |
| **PI490766** | **Georgia** | **III** | **10.77** | **12.97** | **141.6** | **0.36** | **0.12** | **4.67** |
| **PI495020** | **Germany** | **IV** | **4.91** | **10.58** | **99.03** | **0.27** | **0.1** | **4.2** |
| **PI561371** | **Moldova** | **IV** | **7.01** | **9.34** | **93.27** | **0.52** | **0.14** | **5.62** |
| **PI567307** | **NK** | **IV** | **3.57** | **7.84** | **97.57** | **0.31** | **0.09** | **5.21** |
| **PI567415A** | **Russia** | **IV** | **6.75** | **9.29** | **82.47** | **0.7** | **0.15** | **4.93** |
| **PI567416** | **Russia** | **IV** | **6.41** | **7.62** | **88.86** | **0.45** | **0.14** | **6** |
| **PI567428** | **Russia** | **IV** | **5.98** | **9.85** | **119** | **0.31** | **0.1** | **5.63** |
| **PI567435B** | **Russia** | **III** | **7.52** | **11.92** | **111.3** | **0.44** | **0.12** | **4.26** |
| **PI567439** | **Russia** | **V** | **8.18** | **10.07** | **107.3** | **0.34** | **0.11** | **4.69** |
| **PI567488A** | **Russia** | **IV** | **7.56** | **10.36** | **104.2** | **0.51** | **0.16** | **4.57** |
| **PI567532** | **Russia** | **IV** | **5.03** | **11.87** | **99.57** | **0.54** | **0.13** | **4.51** |
| **PI567548** | **Russia** | **IV** | **4.68** | **7.47** | **77.33** | **0.41** | **0.14** | **5.29** |
| **PI567576** | **Russia** | **III** | **7.6** | **10.67** | **85.84** | **0.52** | **0.18** | **4.07** |
| **PI567780B** | **China** | **IV** | **3.83** | **10.45** | **116.7** | **0.31** | **0.1** | **5.18** |
| **PI538386A** | **Japan** | **III** | **6.56** | **12.43** | **154.3** | **0.3** | **0.1** | **5.75** |
| **PI548383** | **Japan** | **III** | **5.11** | **11.47** | **116.3** | **0.29** | **0.09** | **4.75** |
| **PI548400** | **Japan** | **IV** | **5.32** | **13.39** | **112.2** | **0.49** | **0.12** | **4.48** |
| **PI567346** | **NK** | **V** | **4.93** | **17.27** | **140.7** | **0.52** | **0.1** | **4.85** |
| **PI567352A** | **Peru** | **IV** | **14.94** | **14.13** | **138.2** | **0.39** | **0.19** | **4.82** |
| **PI567353** | **Philippines** | **IV** | **7.22** | **13.83** | **142.8** | **0.67** | **0.13** | **5.65** |
| **PI567361** | **Philippines** | **III** | **12.18** | **21.45** | **333** | **0.4** | **0.09** | **6.73** |
| **PI567407** | **Romania** | **V** | **8.53** | **14.98** | **180.5** | **0.33** | **0.11** | **5.81** |
| **PI567408** | **Russia** | **V** | **10.03** | **13.66** | **138.8** | **0.47** | **0.13** | **5.24** |
| **PI567675** | **Russia** | **IV** | **5.78** | **11.71** | **117.6** | **0.42** | **0.12** | **5.31** |
| **PI567685** | **Russia** | **IV** | **7** | **11.91** | **116.7** | **0.53** | **0.13** | **5.35** |
| **PI567698A** | **Russia** | **IV** | **5.24** | **12.74** | **118.3** | **0.39** | **0.11** | **4.55** |
| **PI567726** | **Russia** | **IV** | **5.52** | **11.29** | **130.8** | **0.36** | **0.11** | **5.58** |
| **PI567746** | **Russia** | **IV** | **5.57** | **11.16** | **90.41** | **0.42** | **0.14** | **4.18** |
| **PI574477** | **SK** | **IV** | **7.45** | **13.57** | **135.7** | **0.45** | **0.11** | **4.81** |
| **PI587588B** | **SK** | **V** | **6.99** | **13.47** | **151.4** | **0.34** | **0.11** | **5.35** |
| **PI587712B** | **SK** | **V** | **7.41** | **13.78** | **119.3** | **0.58** | **0.14** | **5.01** |
| **PI068423** | **China** | **III** | **4.78** | **11.61** | **136.2** | **0.36** | **0.1** | **5.52** |
| **PI068523** | **China** | **III** | **10.47** | **14.26** | **206.4** | **0.47** | **0.11** | **7.13** |
| **PI068732_1** | **China** | **II** | **5.62** | **11.01** | **107.7** | **0.52** | **0.13** | **5.32** |
| **PI297505** | **China** | **I** | **4.5** | **9.44** | **110.4** | **0.39** | **0.1** | **5.77** |
| **PI417381** | **Japan** | **0** | **6.13** | **13.73** | **125.7** | **0.54** | **0.11** | **5.22** |
| **PI603495B** | **China** | **V** | **9.79** | **14.09** | **109** | **0.5** | **0.19** | **4.08** |
| **PI603526** | **China** | **IV** | **9.37** | **16.33** | **160.9** | **0.43** | **0.13** | **5.05** |
| **PI603549** | **China** | **III** | **6.57** | **15.18** | **143.8** | **0.5** | **0.12** | **5.07** |
| **PI603556** | **China** | **III** | **6.29** | **17.13** | **182.5** | **0.46** | **0.09** | **5.26** |
| **PI070208** | **China** | **IV** | **2.94** | **12.6** | **128.6** | **0.29** | **0.09** | **4.69** |
| **PI079870-4** | **China** | **IV** | **1.76** | **8.88** | **66.29** | **0.7** | **0.09** | **4.61** |
| **PI430598B** | **China** | **IV** | **4.44** | **11.71** | **96.21** | **0.49** | **0.12** | **4.31** |
| **PI594456A** | **China** | **III** | **1.89** | **9.31** | **80.34** | **0.4** | **0.1** | **4.46** |
| **PI594880** | **China** | **V** | **2.19** | **8.54** | **65.2** | **0.61** | **0.11** | **4.67** |
| **PI594922** | **USA** | **V** | **5.91** | **12.55** | **111.7** | **0.38** | **0.12** | **4.76** |
| **PI597478B** | **SK** | **III** | **2.74** | **11.99** | **83.7** | **0.58** | **0.12** | **3.92** |
| **PI598358** | **Unknown** | **V** | **2.05** | **7.98** | **63.05** | **0.47** | **0.11** | **3.79** |
| **PI606374** | **Vietnam** | **III** | **1.69** | **6.83** | **40.68** | **0.77** | **0.11** | **3.88** |
| **PI632418** | **USA** | **V** | **3.84** | **9.48** | **78.6** | **0.38** | **0.1** | **3.82** |
| **PI093055S** | **China** | **V** | **5.13** | **13.63** | **158.3** | **0.51** | **0.11** | **6.57** |
| **PI093563** | **China** | **III** | **13.11** | **18.46** | **194.8** | **0.48** | **0.16** | **5.54** |
| **PI103079** | **China** | **V** | **5.75** | **8.69** | **89.02** | **0.5** | **0.14** | **4.78** |
| **PI123587** | **Belgium** | **V** | **7.14** | **10.45** | **130.9** | **0.64** | **0.14** | **7.13** |
| **PI235347** | **Japan** | **IV** | **3.5** | **13.61** | **206.9** | **0.49** | **0.09** | **8.44** |
| **PI253656B** | **China** | **IV** | **6.75** | **13.47** | **131.5** | **0.44** | **0.13** | **5.64** |
| **PI378682C** | **Japan** | **IV** | **8.06** | **15.45** | **164.4** | **0.49** | **0.13** | **5.69** |
| **PI603494** | **China** | **IV** | **2.19** | **9.96** | **106** | **0.47** | **0.09** | **5.67** |
| **4J105-3-4** | **USA** | **III** | **5.44** | **12.07** | **112.5** | **0.38** | **0.12** | **4.8** |
| **5M20-2-5-2** | **USA** | **III** | **12.17** | **17.25** | **149.1** | **0.37** | **0.16** | **4.2** |
| **CL0J095-4-6** | **USA** | **III** | **3.55** | **10.9** | **120** | **0.29** | **0.1** | **4.68** |
| **LD02-9050** | **USA** | **IV** | **5** | **9.2** | **89.88** | **0.34** | **0.11** | **4.03** |
| **LG00-3372** | **USA** | **III** | **6.57** | **12.95** | **103.7** | **0.49** | **0.13** | **4.28** |
| **LG03-2979** | **USA** | **III** | **9.48** | **13.71** | **142.9** | **0.37** | **0.13** | **4.78** |
| **LG03-3191** | **USA** | **IV** | **6.65** | **10.97** | **101** | **0.45** | **0.14** | **4.51** |
| **LG04-4717** | **USA** | **III** | **5.01** | **13.13** | **85.39** | **0.66** | **0.14** | **4.11** |
| **LG05-4292** | **USA** | **IV** | **6.55** | **10.84** | **84.79** | **0.47** | **0.16** | **3.87** |
| **LG05-4317** | **USA** | **IV** | **8.22** | **18.14** | **161.8** | **0.7** | **0.12** | **6.21** |
| **LG05-4464** | **USA** | **III** | **12.53** | **17.12** | **210** | **0.44** | **0.11** | **6.21** |
| **LG05-4832** | **USA** | **III** | **10.02** | **18.3** | **171** | **0.51** | **0.12** | **5.14** |
| **LG90-2550** | **USA** | **III** | **3.66** | **13.15** | **104.4** | **0.52** | **0.1** | **4.24** |
| **LG94-1906** | **USA** | **II** | **2.57** | **7.9** | **50.65** | **0.51** | **0.13** | **3.56** |
| **LG98-1605** | **USA** | **III** | **8.11** | **18.53** | **175.2** | **0.48** | **0.11** | **4.65** |
| **NE3001** | **USA** | **III** | **12.26** | **19.02** | **183.1** | **0.45** | **0.14** | **5.03** |
| **PI404188A** | **China** | **II** | **10.45** | **13.2** | **132.5** | **0.44** | **0.14** | **4.39** |
| **Prohio** | **USA** | **III** | **10.44** | **16.78** | **218.7** | **0.36** | **0.12** | **6.82** |
| **S06-13640** | **USA** | **IV** | **2.21** | **8.73** | **112.4** | **0.34** | **0.06** | **5.05** |
| **TN05-3027** | **USA** | **V** | **8.85** | **15.46** | **159.5** | **0.35** | **0.13** | **5.07** |
| **U03-100612** | **USA** | **I** | **15.47** | **16.78** | **180** | **0.38** | **0.17** | **5.11** |
| **PI200503** | **Japan** | **V** | **4.14** | **14.24** | **114.9** | **0.59** | **0.09** | **5.1** |
| **PI209334** | **Japan** | **III** | **9.47** | **11.86** | **129.3** | **0.58** | **0.14** | **5.68** |
| **PI342434** | **Japan** | **V** | **3.97** | **12.22** | **93.45** | **0.69** | **0.13** | **4.98** |
| **PI377574** | **Japan** | **IV** | **5.78** | **10.62** | **88.7** | **0.55** | **0.15** | **4.61** |
| **PI416838** | **Japan** | **V** | **13.15** | **17.27** | **188** | **0.59** | **0.17** | **5.84** |
| **PI417007** | **Japan** | **IV** | **3.04** | **14.5** | **191.9** | **0.35** | **0.08** | **6.56** |
| **PI417015** | **Japan** | **III** | **8.46** | **15.98** | **194** | **0.52** | **0.13** | **6.69** |
| **PI417077** | **Japan** | **III** | **7.8** | **16.22** | **143.3** | **0.52** | **0.14** | **5.06** |
| **PI417479** | **Japan** | **IV** | **9.12** | **14.94** | **150.4** | **0.55** | **0.17** | **5.75** |
| **PI504288** | **Japan** | **V** | **1.19** | **9.07** | **68.73** | **0.57** | **0.08** | **4.1** |
| **PI506933** | **Japan** | **IV** | **6.13** | **12.3** | **78.88** | **0.69** | **0.17** | **3.89** |
| **PI548364** | **Japan** | **IV** | **8.43** | **16.07** | **145.4** | **0.67** | **0.14** | **5.27** |
| **PI561387** | **Japan** | **V** | **2.59** | **9.62** | **82.61** | **0.55** | **0.07** | **5** |
| **PI080837** | **Japan** | **IV** | **3.32** | **16.03** | **218.2** | **0.44** | **0.07** | **6.69** |
| **PI081041** | **Japan** | **III** | **8.78** | **21.13** | **237.6** | **0.5** | **0.11** | **5.63** |
| **PI081785** | **Japan** | **III** | **4.45** | **16.08** | **222.7** | **0.44** | **0.1** | **7.22** |
| **PI084631** | **SK** | **III** | **12.18** | **21.14** | **322** | **0.42** | **0.11** | **7.21** |
| **PI084973** | **Japan** | **III** | **14.68** | **22.34** | **228.2** | **0.56** | **0.15** | **6.23** |
| **PI086084** | **Japan** | **V** | **6.59** | **16.68** | **179.3** | **0.42** | **0.1** | **5.92** |
| **PI086982** | **SK** | **V** | **5.31** | **10.54** | **128.3** | **0.35** | **0.11** | **5.43** |
| **PI091083** | **SK** | **III** | **9.73** | **20.71** | **196.1** | **0.53** | **0.11** | **5.16** |

**Table S2. TRL, TSA, TRV and TRT traits were measured for the root systems imaged in air and in water for 10 plants.** Paired t-test resulted in statistically significant (p<0.05) differences in computed root traits for the two different treatments (air and underwater imaging)

| **Plant** | **TRL (cm)_ Imaged in air** | **TSA (cm2)_ Imaged in air** | **TRV (cm3)_ Imaged in air** | **TRT_ Imaged in air** | **TRL (cm)_Imaged in water** | **TSA (cm2)_ Imaged in water** | **TRV (cm3)_ Imaged in water** | **Tips_ Imaged_in water** |
| --- | --- | --- | --- | --- | --- | --- | --- | --- |
| Image_02 | 49.93 | 13.05 | 64 | 40.89 | 41 | 9.53 | 0.18 | 22 |
| Image_01 | 109.14 | 27.39 | 165 | 79.65 | 80 | 19.20 | 0.37 | 76 |
| Image_06 | 117.23 | 26.83 | 197 | 99.90 | 100 | 21.76 | 0.38 | 72 |
| Image_10 | 142.83 | 34.14 | 202 | 131.54 | 132 | 30.74 | 0.57 | 101 |
| Image_08 | 106.15 | 26.88 | 224 | 88.99 | 89 | 21.25 | 0.40 | 93 |
| Image_05 | 173.12 | 43.10 | 237 | 157.00 | 157 | 38.47 | 0.75 | 102 |
| Image_09 | 150.24 | 34.96 | 243 | 134.21 | 134 | 28.10 | 0.47 | 120 |
| Image_03 | 127.17 | 28.57 | 257 | 118.35 | 118 | 26.01 | 0.46 | 140 |
| Image_04 | 150.64 | 34.26 | 258 | 139.97 | 140 | 30.20 | 0.52 | 102 |
| Image_07 | 155.98 | 34.93 | 272 | 129.36 | 129 | 26.89 | 0.45 | 94 |

**Table S3. TRT_8DAT measured manually and by WinRhizo where the background of the images were computationally cleaned of non-root objects or not cleaned.**

| **PI** | **TRT_8DAT_uncleaned** | **TRT_8DAT_cleaned** | **TRT_8DAT_Manual** |
| --- | --- | --- | --- |
| PI379618 | 108 | 94 | 84 |
| PI424038B | 169 | 98 | 80 |
| PI437110A | 262 | 222 | 199 |
| PI058955 | 130 | 89 | 87 |
| PI209332 | 143 | 90 | 88 |
| PI209332 | 201 | 173 | 189 |
| PI209332 | 147 | 102 | 89 |
| PI437654 | 258 | 213 | 180 |
| PI567558 | 294 | 248 | 245 |
| PI417091 | 198 | 166 | 177 |
| PI417091 | 258 | 183 | 181 |
| PI437169B | 240 | 209 | 202 |
| PI437725 | 249 | 176 | 155 |
| PI468915 | 214 | 173 | 178 |
| PI548619 | 279 | 256 | 246 |
| PI548633 | 138 | 116 | 114 |
| PI548619 | 144 | 144 | 145 |

**Table S4. Root morphological and RSA trait data**. Root morphology traits: Total root length (TRL), Total surface area (TSA), Average root diameter (DIM), Total root volume (TRV), Total number of root tips (TRT), Total surface area of thinner roots (root diameter class of 0 to 0.5 mm diameter [(TSA1)] and surface area of thicker roots (root diameter class of 1 to 1.5 mm diameter [(TSA3]). Root system architecture traits: Total root width (TRW), Total convex area (TCA), Root depth index (RDI), Solidity (SOL), and Center of projected mass (COPM). These two types of traits were calculated from root images at 5 and 8 DAT for the subset of 12 lines that represent a significant phenotypic diversity across the soybean panel

| Line/ Accession at 5DAT | Seq ID | TRL (cm) | TSA (cm2) | DIM (mm) | TRV (cm3) | TRT | TSA1 | TSA3 | TRW (cm) | TCA (cm2) | RDI | SOL | COPM(cm) |
| --- | --- | --- | --- | --- | --- | --- | --- | --- | --- | --- | --- | --- | --- |
| PI548158 | USB-623 | 67.79 | 12.41 | 0.59 | 0.18 | 42.4 | 3.89 | 0.76 | 6.7 | 54.3 | 0.43 | 0.08 | 4.08 |
| PI548313 | USB-023 | 102.7 | 21.17 | 0.65 | 0.35 | 67.3 | 3.45 | 2.34 | 8.79 | 73.33 | 0.27 | 0.09 | 3.67 |
| PI437138 | USB-546 | 157.2 | 34.98 | 0.71 | 0.62 | 83.8 | 4.3 | 4.74 | 12.4 | 91.42 | 0.47 | 0.12 | 4.13 |
| Lee | USB-001 | 144.3 | 28.98 | 0.64 | 0.46 | 72.5 | 4.5 | 2.87 | 11.67 | 97.07 | 0.6 | 0.1 | 4.93 |
| PI408105A | HN049 | 281.3 | 59.76 | 0.68 | 1.01 | 132 | 11.5 | 8.01 | 15.02 | 147.74 | 0.63 | 0.13 | 6.04 |
| PI438500 | USB-208 | 304.4 | 61.05 | 0.64 | 0.98 | 105 | 13.13 | 7.92 | 17.01 | 156.29 | 0.67 | 0.12 | 5.93 |
| PI548200 | USB-253 | 173.6 | 40.23 | 0.74 | 0.75 | 125 | 3.77 | 5.44 | 13.2 | 174.3 | 0.57 | 0.07 | 7.45 |
| PI548359 | USB-257 | 139.1 | 36.76 | 0.85 | 0.78 | 77.8 | 1.66 | 7.69 | 11.78 | 121.05 | 0.33 | 0.1 | 4.7 |
| PI209332 | HN021 | 95.08 | 18.75 | 0.64 | 0.3 | 76.6 | 4.33 | 1.76 | 8.88 | 99.51 | 0.37 | 0.07 | 5.2 |
| PI437169B | HN054 | 177.2 | 41.83 | 0.75 | 0.79 | 106 | 4.06 | 6.12 | 13.42 | 141.44 | 0.28 | 0.1 | 4.53 |
| PI398593 | HN041 | 257.6 | 48.9 | 0.61 | 0.74 | 91.2 | 11.25 | 4.64 | 12.51 | 153.4 | 0.66 | 0.1 | 7.67 |
| PI424608A | HN023 | 280.1 | 64.89 | 0.74 | 1.2 | 118 | 7.09 | 10.14 | 17.32 | 201.49 | 0.63 | 0.1 | 7.04 |
|  |  |  |  |  |  |  |  |  |  |  |  |  |  |
| Line/ Accession at 8DAT | **Seq ID** | **TRL (cm)** | **TSA (cm2)** | **DIM (mm)** | **TRV (cm3)** | **TRT** | **TSA1** | **TSA3** | **TRW (cm)** | **TCA (cm2)** | **RDI** | **SOL** | **COPM** |
| PI548158 | USB-623 | 119.4 | 22.03 | 0.6 | 0.33 | 75.8 | 8.72 | 1.34 | 9.8 | 83.43 | 0.54 | 0.08 | 4.66 |
| PI548313 | USB-023 | 311.5 | 69.8 | 0.71 | 1.25 | 168 | 15.83 | 9.79 | 15.65 | 151.35 | 0.56 | 0.15 | 5.07 |
| PI437138 | USB-546 | 392.6 | 83.07 | 0.67 | 1.4 | 224 | 19.69 | 9.71 | 19.68 | 193.71 | 0.66 | 0.14 | 6.03 |
| Lee | USB-001 | 250.6 | 53.51 | 0.68 | 0.92 | 171 | 9.76 | 6.34 | 17.45 | 176.92 | 0.67 | 0.1 | 6.47 |
| PI408105A | HN049 | 524.1 | 107.99 | 0.66 | 1.78 | 276 | 28.72 | 15.36 | 23.21 | 298.75 | 0.61 | 0.12 | 7.35 |
| PI438500 | USB-208 | 605.3 | 121.74 | 0.64 | 1.96 | 273 | 34.1 | 15.63 | 26.61 | 335.78 | 0.6 | 0.12 | 7.61 |
| PI548200 | USB-253 | 263.8 | 58.96 | 0.72 | 1.05 | 172 | 10.76 | 7.23 | 14.65 | 210.4 | 0.6 | 0.09 | 8.1 |
| PI548359 | USB-257 | 224 | 56.55 | 0.81 | 1.14 | 107 | 7.15 | 10.12 | 16.31 | 226.49 | 0.26 | 0.08 | 5.83 |
| PI209332 | HN021 | 144.5 | 28.22 | 0.62 | 0.44 | 107 | 9.35 | 3.02 | 11.6 | 143.74 | 0.3 | 0.07 | 6.27 |
| PI437169B | HN054 | 350.2 | 80.84 | 0.74 | 1.5 | 201 | 14.28 | 11.48 | 19.36 | 253.03 | 0.37 | 0.11 | 6.23 |
| PI398593 | HN041 | 564.3 | 105.91 | 0.6 | 1.59 | 185 | 37.37 | 9.19 | 19.92 | 367.86 | 0.67 | 0.09 | 10.83 |
| PI424608A | HN023 | 666.5 | 143.14 | 0.68 | 2.46 | 291 | 36.76 | 20.99 | 28.02 | 434.92 | 0.67 | 0.11 | 10.19 |

**Table S5. Pearson correlation coefficient among RSA traits at 5 and 8DAT**

Note (abbreviations for traits): Total root length (TRL), Total root volume (TRV), Average root diameter (DIM), Total surface area (TSA), Total surface area of thinner roots (or diameter class 0 to 0.5 mm diameter [(TSA1)] and surface area of thicker roots (or diameter class 1 to 1.5 mm diameter [(TSA3]), Total root width (TRW), Total convex area (TCA), Root depth index (RDI), and Solidity (SOL) and Center of projected mass (COPM) phenotype were calculated from root images at 8 DAT.

**Table S6. Significantly associated GWAS peaks for root morphological and architectural traits. Here, the abbreviations Chrom, Pos, Ref, Alt and Pval represent chromosome, position of SNP, reference allele, alternative allele, and p-value of association, respectively**.

| Trait | Name of QTL | Locus | Chrom | Pos | Ref | Alt | Effect | Pval |
| --- | --- | --- | --- | --- | --- | --- | --- | --- |
| COPM_8DAT | QCOPM_8DAT_01 | 4_48983360 | 4 | 48983360 | C | T | 0.680145 | 2.39E-08 |
|  | QCOPM_8DAT_02 | 4_48987529 | 4 | 48987529 | A | T | 0.663275 | 3.38E-08 |
|  | QCOPM_8DAT_03 | 4_48999800 | 4 | 48999800 | A | T | 0.636885 | 1.46E-07 |
|  | QCOPM_8DAT_04 | 7_11693371 | 7 | 11693371 | T | G | -0.75647 | 3.71E-08 |
|  | QCOPM_8DAT_05 | 9_45362184 | 9 | 45362184 | A | T | 1.344201 | 2.28E-08 |
|  | QCOPM_8DAT_06 | 13_5946151 | 13 | 5946151 | T | G | 0.913594 | 7.08E-08 |
|  | QCOPM_8DAT_07 | 20_30874231 | 20 | 30874231 | C | T | 1.140248 | 1.06E-07 |
|  | QCOPM_8DAT_08 | 20_31008509 | 20 | 31008509 | T | C | 1.07492 | 4.36E-08 |
|  | QCOPM_8DAT_09 | 20_31068498 | 20 | 31068498 | C | A | 1.140248 | 1.06E-07 |
|  | QCOPM_8DAT_10 | 20_31082519 | 20 | 31082519 | G | A | 1.133677 | 7.93E-08 |
|  | QCOPM_8DAT_11 | 20_31084832 | 20 | 31084832 | A | T | 1.25708 | 1.93E-08 |
|  | QCOPM_8DAT_12 | 20_31095132 | 20 | 31095132 | C | T | 1.499334 | 7.94E-09 |
|  | QCOPM_8DAT_13 | 20_31109667 | 20 | 31109667 | C | A | 1.140248 | 1.06E-07 |
|  | QCOPM_8DAT_14 | 20_31126763 | 20 | 31126763 | G | A | 1.140248 | 1.06E-07 |
|  | QCOPM_8DAT_15 | 20_42398067 | 20 | 42398067 | G | A | 0.573393 | 1.14E-07 |
|  | QCOPM_8DAT_16 | 20_42416517 | 20 | 42416517 | A | G | 0.555116 | 1.43E-07 |
| DIM_8DAT | QDIM_8DAT_01 | 1_49094594 | 1 | 49094594 | G | A | 0.047962 | 1.85E-07 |
|  | QDIM_8DAT_02 | 1_49130941 | 1 | 49130941 | T | C | 0.047962 | 1.85E-07 |
|  | QDIM_8DAT_03 | 1_49305802 | 1 | 49305802 | C | T | 0.044081 | 1.12E-07 |
|  | QDIM_8DAT_04 | 1_49408048 | 1 | 49408048 | C | T | 0.043585 | 1.06E-07 |
|  | QDIM_8DAT_05 | 5_34851234 | 5 | 34851234 | A | T | 0.039663 | 9.83E-08 |
|  | QDIM_8DAT_06 | 5_34954486 | 5 | 34954486 | G | T | 0.03861 | 1.20E-08 |
|  | QDIM_8DAT_07 | 12_23394150 | 12 | 23394150 | G | A | 0.055134 | 1.45E-07 |
|  | QDIM_8DAT_08 | 19_42651593 | 19 | 42651593 | C | T | 0.041165 | 1.55E-07 |
| SOL_5DAT | QSOL_5DAT_01 | 8_21632858 | 8 | 21632858 | G | A | 0.014492 | 1.73E-07 |
|  | QSOL_5DAT_02 | 20_2376802 | 20 | 2376802 | G | A | 0.015079 | 5.17E-08 |
|  | QSOL_5DAT_03 | 20_6775659 | 20 | 6775659 | G | T | 0.01625 | 1.07E-07 |
| TCA_8DAT | QTCA_8DAT_01 | 4_2927015 | 4 | 2927015 | C | A | 122.9242 | 1.92E-07 |
|  | QTCA_8DAT_02 | 4_46988148 | 4 | 46988148 | A | T | 89.17831 | 6.60E-08 |
|  | QTCA_8DAT_03 | 4_47003213 | 4 | 47003213 | C | T | 76.40829 | 1.56E-07 |
|  | QTCA_8DAT_04 | 4_47068983 | 4 | 47068983 | C | G | 67.46801 | 1.69E-08 |
|  | QTCA_8DAT_05 | 4_47196118 | 4 | 47196118 | T | C | 49.47637 | 2.05E-07 |
|  | QTCA_8DAT_06 | 4_47287834 | 4 | 47287834 | G | T | 47.49867 | 2.51E-08 |
|  | QTCA_8DAT_07 | 4_47296426 | 4 | 47296426 | A | T | 47.22484 | 9.15E-08 |
|  | QTCA_8DAT_08 | 4_47519634 | 4 | 47519634 | A | T | 83.18458 | 2.03E-07 |
|  | QTCA_8DAT_09 | 6_6108102 | 6 | 6108102 | G | A | 33.42475 | 9.48E-08 |
|  | QTCA_8DAT_10 | 6_48519879 | 6 | 48519879 | A | G | 50.07371 | 1.82E-07 |
|  | QTCA_8DAT_11 | 8_5449445 | 8 | 5449445 | G | A | 79.56402 | 1.51E-07 |
|  | QTCA_8DAT_12 | 18_48664810 | 18 | 48664810 | A | G | 81.03698 | 6.47E-08 |
|  | QTCA_8DAT_13 | 18_48669342 | 18 | 48669342 | G | A | 77.8466 | 2.53E-08 |
| TRL_8DAT | QTRL_8DAT_01 | 2_8678721 | 2 | 8678721 | T | A | 63.40509 | 9.90E-08 |
|  | QTRL_8DAT_02 | 4_5237891 | 4 | 5237891 | C | T | 194.8517 | 2.08E-08 |
|  | QTRL_8DAT_03 | 4_5747201 | 4 | 5747201 | T | C | 194.8517 | 2.08E-08 |
|  | QTRL_8DAT_04 | 4_47283354 | 4 | 47283354 | G | A | 70.58388 | 8.27E-08 |
|  | QTRL_8DAT_05 | 11_17101313 | 11 | 17101313 | T | C | 68.3233 | 9.69E-08 |
| TRT_5DAT | QTRT_5DAT_01 | 6_42364596 | 6 | 42364596 | T | A | 20.85535 | 1.20E-07 |
|  | QTRT_5DAT_02 | 8_37354009 | 8 | 37354009 | C | A | -23.9854 | 1.43E-07 |
|  | QTRT_5DAT_03 | 14_30234317 | 14 | 30234317 | T | C | -9.37199 | 1.16E-07 |
|  | QTRT_5DAT_04 | 14_30260028 | 14 | 30260028 | G | C | -9.28027 | 1.46E-07 |
|  | QTRT_5DAT_05 | 14_30266138 | 14 | 30266138 | A | G | -9.24846 | 1.25E-07 |
|  | QTRT_5DAT_06 | 14_30269261 | 14 | 30269261 | A | T | -9.28935 | 1.12E-07 |
|  | QTRT_5DAT_07 | 14_30271616 | 14 | 30271616 | C | T | -9.23488 | 1.69E-07 |
|  | QTRT_5DAT_08 | 14_30302634 | 14 | 30302634 | C | T | -9.30877 | 9.94E-08 |
|  | QTRT_5DAT_09 | 14_30304173 | 14 | 30304173 | A | G | -9.50248 | 1.24E-07 |
|  | QTRT_5DAT_10 | 14_30311444 | 14 | 30311444 | G | A | -9.29049 | 1.71E-07 |
|  | QTRT_5DAT_11 | 14_30314214 | 14 | 30314214 | A | G | -9.3055 | 1.10E-07 |
|  | QTRT_5DAT_12 | 14_30321217 | 14 | 30321217 | G | A | -10.0565 | 1.70E-07 |
|  | QTRT_5DAT_13 | 14_30385210 | 14 | 30385210 | A | G | -9.14064 | 1.66E-07 |
|  | QTRT_5DAT_14 | 14_30404378 | 14 | 30404378 | C | T | -9.26319 | 1.21E-07 |
|  | QTRT_5DAT_15 | 14_30407475 | 14 | 30407475 | A | G | -10.3288 | 1.43E-07 |
|  | QTRT_5DAT_16 | 14_30419829 | 14 | 30419829 | T | C | -9.14903 | 1.58E-07 |
|  | QTRT_5DAT_17 | 14_30437463 | 14 | 30437463 | C | T | -9.0775 | 1.91E-07 |
|  | QTRT_5DAT_18 | 14_30477150 | 14 | 30477150 | A | G | -9.5581 | 8.96E-08 |
|  | QTRT_5DAT_19 | 14_30520875 | 14 | 30520875 | C | T | -9.0767 | 1.93E-07 |
|  | QTRT_5DAT_20 | 14_30529821 | 14 | 30529821 | T | G | -9.07827 | 1.96E-07 |
|  | QTRT_5DAT_21 | 14_30539409 | 14 | 30539409 | C | A | -9.2711 | 1.28E-07 |
|  | QTRT_5DAT_22 | 14_30555465 | 14 | 30555465 | G | A | -12.1882 | 5.46E-08 |
|  | QTRT_5DAT_23 | 14_30570439 | 14 | 30570439 | G | A | -9.34055 | 1.47E-07 |
|  | QTRT_5DAT_24 | 14_30577855 | 14 | 30577855 | G | A | -9.12709 | 1.79E-07 |
|  | QTRT_5DAT_25 | 14_30592345 | 14 | 30592345 | G | C | -9.70898 | 1.29E-07 |
|  | QTRT_5DAT_26 | 14_30624825 | 14 | 30624825 | C | T | -9.34065 | 8.73E-08 |
|  | QTRT_5DAT_27 | 14_30630782 | 14 | 30630782 | C | G | -9.11867 | 1.85E-07 |
| TRT_8DAT | QTRT_8DAT_01 | 9_37109514 | 9 | 37109514 | T | A | -41.9438 | 1.44E-07 |
| TRV_5DAT | QTRV_5DAT_01 | 2_9964213 | 2 | 9964213 | A | G | 0.143404 | 1.35E-07 |
|  | QTRV_5DAT_02 | 5_38783033 | 5 | 38783033 | C | T | 0.63952 | 9.33E-08 |
|  | QTRV_5DAT_03 | 5_39210068 | 5 | 39210068 | T | A | 0.63952 | 9.33E-08 |
|  | QTRV_5DAT_04 | 7_4869847 | 7 | 4869847 | A | C | 0.63952 | 9.33E-08 |
|  | QTRV_5DAT_05 | 10_17091783 | 10 | 17091783 | G | A | -1.27904 | 9.33E-08 |
|  | QTRV_5DAT_06 | 11_9667534 | 11 | 9667534 | G | A | 0.63952 | 9.33E-08 |
|  | QTRV_5DAT_07 | 17_38507011 | 17 | 38507011 | A | G | 0.63952 | 9.33E-08 |
|  | QTRV_5DAT_08 | 18_3162542 | 18 | 3162542 | A | T | 0.197681 | 3.05E-08 |
|  | QTRV_5DAT_09 | 18_51917831 | 18 | 51917831 | G | A | 0.236272 | 1.48E-07 |
|  | QTRV_5DAT_10 | 20_44767970 | 20 | 44767970 | G | A | 0.149565 | 1.27E-07 |
|  | QTRV_5DAT_11 | 20_44824392 | 20 | 44824392 | T | C | 0.149565 | 1.27E-07 |
| TRV_8DAT | QTRV_8DAT_01 | 9_34781750 | 9 | 34781750 | T | C | 0.326209 | 1.48E-07 |
|  | QTRV_8DAT_02 | 11_7681279 | 11 | 7681279 | T | A | 0.437795 | 1.36E-07 |
|  | QTRV_8DAT_03 | 11_7717086 | 11 | 7717086 | A | C | 0.447129 | 1.21E-07 |
| TRW_5DAT | QTRW_5DAT_01 | 18_2256513 | 18 | 2256513 | G | A | 2.281548 | 1.19E-07 |
| TRW_8DAT | QTRW_8DAT_01 | 5_3700779 | 5 | 3700779 | C | T | 2.397477 | 1.24E-07 |
| TSA_5DAT | QTSA_5DAT_01 | 19_50434355 | 19 | 50434355 | A | C | -8.23461 | 1.88E-07 |
| TSA1_5DAT | QTSA1_5DAT_01 | 15_49535875 | 15 | 49535875 | T | A | 1.554863 | 1.06E-07 |
| TSA1_8DAT | QTSA1_8DAT_01 | 1_41808 | 1 | 41808 | T | C | -3.77116 | 3.38E-14 |
|  | QTSA1_8DAT_02 | 3_4703917 | 3 | 4703917 | G | A | 4.074945 | 6.69E-08 |
|  | QTSA1_8DAT_03 | 18_12434353 | 18 | 12434353 | G | A | -3.06732 | 5.32E-10 |
| TSA3_5DAT | QTSA3_5DAT_01 | 6_42440630 | 6 | 42440630 | G | A | 1.103929 | 1.21E-07 |
|  | QTSA3_5DAT_02 | 12_22382546 | 12 | 22382546 | C | T | 1.465376 | 1.49E-07 |
| TSA3_8DAT | QTSA3_8DAT_01 | 20_42427993 | 20 | 42427993 | A | G | 1.610244 | 9.06E-09 |
|  | QTSA3_8DAT_02 | 20_42455025 | 20 | 42455025 | T | A | 1.475215 | 4.87E-08 |

Note (abbreviations for traits): Total root length (TRL), Total root volume (TRV), Average root diameter (DIM), Total surface area (TSA), Total surface area of thinner roots (or diameter class 0 to 0.5 mm diameter [(TSA1)] and surface area of thicker roots (or diameter class 1 to 1.5 mm diameter [(TSA3]), Total root width (TRW), Total convex area (TCA), Root depth index (RDI), and Solidity (SOL) and Center of projected mass (COPM) phenotype were calculated from root images at 5 and 8 DAT.

**Table S7. GWAS peaks significantly associated with two or more than two traits.** Abbreviations for traits: Total root length (TRL), Total root volume (TRV), Average root diameter (DIM), Total surface area (TSA), Total surface area of thinner roots (for diameter class 0 to 0.5 mm in diameter [(TSA1)] and surface area of thicker roots (or diameter class 1 to 1.5 mm diameter [(TSA3]), Total root system width (TRW), Total convex area (TCA), Root depth index (RDI), and Solidity (SOL) and Center of projected mass (COPM) traits were calculated from root images taken at 5 and 8 DAT.

| Trait | Locus | Chrom | Pos | Ref | Alt | Pval |
| --- | --- | --- | --- | --- | --- | --- |
| TRL_5DAT, TSA_5DAT | 9_22018271 | 9 | 22018271 | A | C | 4.65E-08 |
| TRL_8DAT, TSA_8DAT | 2_9448766 | 2 | 9448766 | G | T | 1.52E-07 |
| TRL_8DAT, TSA_8DAT | 17_34890177 | 17 | 34890177 | C | T | 1.45E-08 |
| TRL_8DAT, TSA1_8DAT | 2_8593061 | 2 | 8593061 | G | A | 1.75E-07 |
| TRV_5DAT, TSA3_8DAT | 2_9970564 | 2 | 9970564 | A | T | 1.19E-07 |
| TSA_8DAT, TRV_8DAT | 2_9883206 | 2 | 9883206 | G | A | 5.00E-08 |
| TSA3_5DAT, TRV_5DAT | 2_9962542 | 2 | 9962542 | T | A | 1.27E-07 |
| TSA3_5DAT, TRV_5DAT | 20_42464855 | 20 | 42464855 | A | G | 1.67E-07 |
| TSA3_5DAT, TRV_5DAT, TSA_5DAT | 4_10814580 | 4 | 10814580 | T | A | 1.42E-08 |
| TSA3_8DAT, TRV_5DAT, TSA3_5DAT | 20_42450695 | 20 | 42450695 | C | T | 1.09E-07 |
| TSA3_8DAT, TRV_8DAT | 12_33823378 | 12 | 33823378 | T | C | 3.41E-08 |
| TCA_8DAT, TRL_8DAT | 4_47289825 | 4 | 47289825 | G | T | 1.31E-07 |
| TCA_8DAT, TRL_8DAT | 4_47367011 | 4 | 47367011 | G | A | 1.90E-07 |
| TCA_8DAT, TRL_8DAT | 6_48522645 | 6 | 48522645 | C | G | 1.97E-07 |
| TCA_8DAT, TRL_8DAT | 11_5464119 | 11 | 5464119 | G | A | 1.15E-07 |

**Table S8. Genomic hotspots for root morphological and architectural traits.** Here abbreviations for Chrom, Pos, Ref, Alt and Pval represent chromosome, position of SNP, reference allele, alternative allele and p-value of association respectively.

| Hotspot | QTL | Chrom | Position | Locus | Ref | Alt | Effect | Pval | |  |
| --- | --- | --- | --- | --- | --- | --- | --- | --- | --- | --- |
| Hotspot_1 | PPQ02(TRL_8DAT, TSA_8DAT) | 2 | 9448766 | 2_9448766 | G | T | 73.7811 | | 1.52E-07 | |
|  | PPQ06(TSA_8DAT, TRV_8DAT) | 2 | 9883206 | 2_9883206 | G | A | 0.33837 | | 5.00E-08 | |
|  | PPQ07(TSA3_5DAT, TRV_5DAT) | 2 | 9962542 | 2_9962542 | T | A | 1.2487 | | 1.27E-07 | |
|  | QTRV_5DAT_01 | 2 | 9964213 | 2_9964213 | A | G | 0.1434 | | 1.35E-07 | |
|  | PPQ05(TRV_5DAT, TSA3_8DAT) | 2 | 9970564 | 2_9970564 | A | T | 1.24107 | | 1.19E-07 | |
| Hotspot_2 | QTCA_8DAT_02 | 4 | 46988148 | 4_46988148 | A | T | 89.1783 | | 6.60E-08 | |
|  | QTCA_8DAT_03 | 4 | 47003213 | 4_47003213 | C | T | 76.4083 | | 1.56E-07 | |
|  | QTCA_8DAT_04 | 4 | 47068983 | 4_47068983 | C | G | 67.468 | | 1.69E-08 | |
|  | QTCA_8DAT_05 | 4 | 47196118 | 4_47196118 | T | C | 49.4764 | | 2.05E-07 | |
|  | QTRL_8DAT_04 | 4 | 47283354 | 4_47283354 | G | A | 70.5839 | | 8.27E-08 | |
|  | QTCA_8DAT_06 | 4 | 47287834 | 4_47287834 | G | T | 47.4987 | | 2.51E-08 | |
|  | PPQ13(TRL_8DAT, TCA_8DAT) | 4 | 47289825 | 4_47289825 | G | T | 68.1947 | | 1.31E-07 | |
|  | QTCA_8DAT_07 | 4 | 47296426 | 4_47296426 | A | T | 47.2248 | | 9.15E-08 | |
|  | PPQ14(TRL_8DAT, TCA_8DAT) | 4 | 47367011 | 4_47367011 | G | A | 76.3249 | | 1.90E-07 | |
| Hotspot_3 | QTRT_5DAT_03 | 14 | 30234317 | 14_30234317 | T | C | -9.37199 | | 1.16E-07 | |
|  | QTRT_5DAT_04 | 14 | 30260028 | 14_30260028 | G | C | -9.28027 | | 1.46E-07 | |
|  | QTRT_5DAT_05 | 14 | 30266138 | 14_30266138 | A | G | -9.24846 | | 1.25E-07 | |
|  | QTRT_5DAT_06 | 14 | 30269261 | 14_30269261 | A | T | -9.28935 | | 1.12E-07 | |
|  | QTRT_5DAT_07 | 14 | 30271616 | 14_30271616 | C | T | -9.23488 | | 1.69E-07 | |
|  | QTRT_5DAT_08 | 14 | 30302634 | 14_30302634 | C | T | -9.30877 | | 9.94E-08 | |
|  | QTRT_5DAT_09 | 14 | 30304173 | 14_30304173 | A | G | -9.50248 | | 1.24E-07 | |
|  | QTRT_5DAT_10 | 14 | 30311444 | 14_30311444 | G | A | -9.29049 | | 1.71E-07 | |
|  | QTRT_5DAT_11 | 14 | 30314214 | 14_30314214 | A | G | -9.3055 | | 1.10E-07 | |
|  | QTRT_5DAT_12 | 14 | 30321217 | 14_30321217 | G | A | -10.0565 | | 1.70E-07 | |
|  | QTRT_5DAT_13 | 14 | 30385210 | 14_30385210 | A | G | -9.14064 | | 1.66E-07 | |
|  | QTRT_5DAT_14 | 14 | 30404378 | 14_30404378 | C | T | -9.26319 | | 1.21E-07 | |
|  | QTRT_5DAT_15 | 14 | 30407475 | 14_30407475 | A | G | -10.3288 | | 1.43E-07 | |
|  | QTRT_5DAT_16 | 14 | 30419829 | 14_30419829 | T | C | -9.14903 | | 1.58E-07 | |
|  | QTRT_5DAT_17 | 14 | 30437463 | 14_30437463 | C | T | -9.0775 | | 1.91E-07 | |
|  | QTRT_5DAT_18 | 14 | 30477150 | 14_30477150 | A | G | -9.5581 | | 8.96E-08 | |
|  | QTRT_5DAT_19 | 14 | 30520875 | 14_30520875 | C | T | -9.0767 | | 1.93E-07 | |
|  | QTRT_5DAT_20 | 14 | 30529821 | 14_30529821 | T | G | -9.07827 | | 1.96E-07 | |
|  | QTRT_5DAT_21 | 14 | 30539409 | 14_30539409 | C | A | -9.2711 | | 1.28E-07 | |
|  | QTRT_5DAT_22 | 14 | 30555465 | 14_30555465 | G | A | -12.1882 | | 5.46E-08 | |
|  | QTRT_5DAT_23 | 14 | 30570439 | 14_30570439 | G | A | -9.34055 | | 1.47E-07 | |
|  | QTRT_5DAT_24 | 14 | 30577855 | 14_30577855 | G | A | -9.12709 | | 1.79E-07 | |
|  | QTRT_5DAT_25 | 14 | 30592345 | 14_30592345 | G | C | -9.70898 | | 1.29E-07 | |
|  | QTRT_5DAT_26 | 14 | 30624825 | 14_30624825 | C | T | -9.34065 | | 8.73E-08 | |
|  | QTRT_5DAT_27 | 14 | 30630782 | 14_30630782 | C | G | -9.11867 | | 1.85E-07 | |
| Hotspot_4 | QCOPM_8DAT_07 | 20 | 30874231 | 20_30874231 | C | T | 1.14025 | | 1.06E-07 | |
|  | QCOPM_8DAT_08 | 20 | 31008509 | 20_31008509 | T | C | 1.07492 | | 4.36E-08 | |
|  | QCOPM_8DAT_09 | 20 | 31068498 | 20_31068498 | C | A | 1.14025 | | 1.06E-07 | |
|  | QCOPM_8DAT_10 | 20 | 31082519 | 20_31082519 | G | A | 1.13368 | | 7.93E-08 | |
|  | QCOPM_8DAT_11 | 20 | 31084832 | 20_31084832 | A | T | 1.25708 | | 1.93E-08 | |
|  | QCOPM_8DAT_12 | 20 | 31095132 | 20_31095132 | C | T | 1.49933 | | 7.94E-09 | |
|  | QCOPM_8DAT_13 | 20 | 31109667 | 20_31109667 | C | A | 1.14025 | | 1.06E-07 | |
|  | QCOPM_8DAT_14 | 20 | 31126763 | 20_31126763 | G | A | 1.14025 | | 1.06E-07 | |
| Hotspot_5 | QCOPM_8DAT_15 | 20 | 42398067 | 20_42398067 | G | A | 0.57339 | | 1.14E-07 | |
|  | QCOPM_8DAT_16 | 20 | 42416517 | 20_42416517 | A | G | 0.55512 | | 1.43E-07 | |
|  | QTSA3_8DAT_01 | 20 | 42427993 | 20_42427993 | A | G | 1.61024 | | 9.06E-09 | |
|  | PPQ11(TSA3_8DAT, TRV_5DAT, TSA3_5DAT) | 20 | 42450695 | 20_42450695 | C | T | 1.4431 | | 1.09E-07 | |
|  | QTSA3_8DAT_02 | 20 | 42455025 | 20_42455025 | T | A | 1.47521 | | 4.87E-08 | |
|  | PPQ09(TSA3_5DAT, TRV_5DAT) | 20 | 42463128 | 20_42463128 | T | G | 0.72419 | | 6.10E-08 | |

Note (abbreviations for traits): Total root length (TRL), Total root volume (TRV), Average root diameter (DIM), Total surface area (TSA), Total surface area of thinner roots (or diameter class 0 to 0.5 mm diameter [(TSA1)] and surface area of thicker roots (or diameter class 1 to 1.5 mm diameter [(TSA3]), Total root width (TRW), Total convex area (TCA), Root depth index (RDI), and Solidity (SOL) and Center of projected mass (COPM) phenotype were calculated from root images at 5 and 8 DAT.

**Table S9. Significantly associated GWAS peaks co-localized with root QTLs identified in previous studies. Here abbreviations for Chrom, Pos, Ref, Alt and Pval represent chromosome, position of SNP, reference allele, alternative allele and p-value of association respectively.**

| Name of QTL | Locus | Chrom | Pos | Ref | Alt | Effect | QTL overlap with previous studies |
| --- | --- | --- | --- | --- | --- | --- | --- |
| QCOPM_8DAT_01 | 4_48983360 | 4 | 48983360 | C | T | 0.680145 | Dry root weight (Zhang et al. 2016) |
| QCOPM_8DAT_02 | 4_48987529 | 4 | 48987529 | A | T | 0.663275 | Dry root weight (Zhang et al. 2016) |
| QCOPM_8DAT_03 | 4_48999800 | 4 | 48999800 | A | T | 0.636885 | Dry root weight (Zhang et al. 2016) |
| QCOPM_8DAT_04 | 7_11693371 | 7 | 11693371 | T | G | -0.75647 | Root volume (Liang et al. 2014) |
| QCOPM_8DAT_07 | 20_30874231 | 20 | 30874231 | C | T | 1.140248 | Lateral root density (Liang et al. 2014) |
| QCOPM_8DAT_08 | 20_31008509 | 20 | 31008509 | T | C | 1.07492 | Lateral root density (Liang et al. 2014) |
| QCOPM_8DAT_09 | 20_31068498 | 20 | 31068498 | C | A | 1.140248 | Lateral root density (Liang et al. 2014) |
| QCOPM_8DAT_10 | 20_31082519 | 20 | 31082519 | G | A | 1.133677 | Lateral root density (Liang et al. 2014) |
| QCOPM_8DAT_11 | 20_31084832 | 20 | 31084832 | A | T | 1.25708 | Lateral root density (Liang et al. 2014) |
| QCOPM_8DAT_12 | 20_31095132 | 20 | 31095132 | C | T | 1.499334 | Lateral root density (Liang et al. 2014) |
| QCOPM_8DAT_13 | 20_31109667 | 20 | 31109667 | C | A | 1.140248 | Lateral root density (Liang et al. 2014) |
| QCOPM_8DAT_14 | 20_31126763 | 20 | 31126763 | G | A | 1.140248 | Lateral root density (Liang et al. 2014) |
| QDIM_8DAT_01 | 1_49094594 | 1 | 49094594 | G | A | 0.047962 | Primary root length (Wu et al. 2012) |
| QDIM_8DAT_02 | 1_49130941 | 1 | 49130941 | T | C | 0.047962 | Primary root length (Wu et al. 2012) |
| QDIM_8DAT_03 | 1_49305802 | 1 | 49305802 | C | T | 0.044081 | Primary root length (Wu et al. 2012) |
| QDIM_8DAT_04 | 1_49408048 | 1 | 49408048 | C | T | 0.043585 | Primary root length (Wu et al. 2012) |
| QDIM_8DAT_07 | 12_23394150 | 12 | 23394150 | G | A | 0.055134 | Root area , root length (Nguyen et al. 2017) |
| QSOL_5DAT_01 | 8_21632858 | 8 | 21632858 | G | A | 0.014492 | Dry root weight (Zhang et al. 2009) |
| QTCA_8DAT_03 | 4_47003213 | 4 | 47003213 | C | T | 76.40829 | Root bushiness (Abdel-Haleem et al. 2010) |
| QTCA_8DAT_04 | 4_47068983 | 4 | 47068983 | C | G | 67.46801 | Root bushiness (Abdel-Haleem et al. 2010) |
| QTCA_8DAT_05 | 4_47196118 | 4 | 47196118 | T | C | 49.47637 | Root bushiness (Abdel-Haleem et al. 2010) |
| QTCA_8DAT_06 | 4_47287834 | 4 | 47287834 | G | T | 47.49867 | Root bushiness (Abdel-Haleem et al. 2010) |
| QTCA_8DAT_07 | 4_47296426 | 4 | 47296426 | A | T | 47.22484 | Root bushiness (Abdel-Haleem et al. 2010) |
| QTCA_8DAT_08 | 4_47519634 | 4 | 47519634 | A | T | 83.18458 | Root bushiness (Abdel-Haleem et al. 2010) |
| QTCA_8DAT_10 | 6_48519879 | 6 | 48519879 | A | G | 50.07371 | Primary root length (Brensha et al. 2012) |
| QTRL_8DAT_04 | 4_47283354 | 4 | 47283354 | G | A | 70.58388 | Root bushiness (Abdel-Haleem et al. 2010) |
| QTRL_8DAT_05 | 11_17101313 | 11 | 17101313 | T | C | 68.3233 | Lateral root density (Liang et al. 2010), Root area (Liang et al., 2010) Root diameter (Nguyen et al. 2017), root length (Liang et al. 2010), root weight (Liang et al. 2010) |
| QTRT_5DAT_01 | 6_42364596 | 6 | 42364596 | T | A | 20.85535 | Dry root weight (Zhang et al. 2016) |
| QTRT_5DAT_02 | 8_37354009 | 8 | 37354009 | C | A | -23.9854 | Dry root weight (Zhang et al. 2009) |
| QTRT_5DAT_03 | 14_30234317 | 14 | 30234317 | T | C | -9.37199 | Dry root weight (Zhang et al. 2016) |
| QTRT_5DAT_04 | 14_30260028 | 14 | 30260028 | G | C | -9.28027 | Dry root weight (Zhang et al. 2016) |
| QTRT_5DAT_05 | 14_30266138 | 14 | 30266138 | A | G | -9.24846 | Dry root weight (Zhang et al. 2016) |
| QTRT_5DAT_06 | 14_30269261 | 14 | 30269261 | A | T | -9.28935 | Dry root weight (Zhang et al. 2016) |
| QTRT_5DAT_07 | 14_30271616 | 14 | 30271616 | C | T | -9.23488 | Dry root weight (Zhang et al. 2016) |
| QTRT_5DAT_08 | 14_30302634 | 14 | 30302634 | C | T | -9.30877 | Dry root weight (Zhang et al. 2016) |
| QTRT_5DAT_09 | 14_30304173 | 14 | 30304173 | A | G | -9.50248 | Dry root weight (Zhang et al. 2016) |
| QTRT_5DAT_10 | 14_30311444 | 14 | 30311444 | G | A | -9.29049 | Dry root weight (Zhang et al. 2016) |
| QTRT_5DAT_11 | 14_30314214 | 14 | 30314214 | A | G | -9.3055 | Dry root weight (Zhang et al. 2016) |
| QTRT_5DAT_12 | 14_30321217 | 14 | 30321217 | G | A | -10.0565 | Dry root weight (Zhang et al. 2016) |
| QTRT_5DAT_13 | 14_30385210 | 14 | 30385210 | A | G | -9.14064 | Dry root weight (Zhang et al. 2016) |
| QTRT_5DAT_14 | 14_30404378 | 14 | 30404378 | C | T | -9.26319 | Dry root weight (Zhang et al. 2016) |
| QTRT_5DAT_15 | 14_30407475 | 14 | 30407475 | A | G | -10.3288 | Dry root weight (Zhang et al. 2016) |
| QTRT_5DAT_16 | 14_30419829 | 14 | 30419829 | T | C | -9.14903 | Dry root weight (Zhang et al. 2016) |
| QTRT_5DAT_17 | 14_30437463 | 14 | 30437463 | C | T | -9.0775 | Dry root weight (Zhang et al. 2016) |
| QTRT_5DAT_18 | 14_30477150 | 14 | 30477150 | A | G | -9.5581 | Dry root weight (Zhang et al. 2016) |
| QTRT_5DAT_19 | 14_30520875 | 14 | 30520875 | C | T | -9.0767 | Dry root weight (Zhang et al. 2016) |
| QTRT_5DAT_20 | 14_30529821 | 14 | 30529821 | T | G | -9.07827 | Dry root weight (Zhang et al. 2016) |
| QTRT_5DAT_21 | 14_30539409 | 14 | 30539409 | C | A | -9.2711 | Dry root weight (Zhang et al. 2016) |
| QTRT_5DAT_22 | 14_30555465 | 14 | 30555465 | G | A | -12.1882 | Dry root weight (Zhang et al. 2016) |
| QTRT_5DAT_23 | 14_30570439 | 14 | 30570439 | G | A | -9.34055 | Dry root weight (Zhang et al. 2016) |
| QTRT_5DAT_24 | 14_30577855 | 14 | 30577855 | G | A | -9.12709 | Dry root weight (Zhang et al. 2016) |
| QTRT_5DAT_25 | 14_30592345 | 14 | 30592345 | G | C | -9.70898 | Dry root weight (Zhang et al. 2016) |
| QTRT_5DAT_26 | 14_30624825 | 14 | 30624825 | C | T | -9.34065 | Dry root weight (Zhang et al. 2016) |
| QTRT_5DAT_27 | 14_30630782 | 14 | 30630782 | C | G | -9.11867 | Dry root weight (Zhang et al. 2016) |
| QTRT_8DAT_01 | 9_37109514 | 9 | 37109514 | T | A | -41.9438 | Dry root weight (Zhang et al. 2016) |
| QTRV_5DAT_05 | 10_17091783 | 10 | 17091783 | G | A | -1.27904 | Primary root length (Liang et al. 2014), root volume (Prince et al. 2019) |
| QTRV_5DAT_06 | 11_9667534 | 11 | 9667534 | G | A | 0.63952 | Root volume (Prince et al. 2019) |
| QTRV_5DAT_08 | 18_3162542 | 18 | 3162542 | A | T | 0.197681 | primary root width (Brensha et al. 2012), Lateral root Density (Brensha et al. 2012), dry root weight (Liang 2014), root volume (prince et al. 2019) |
| QTRV_8DAT_01 | 9_34781750 | 9 | 34781750 | T | C | 0.326209 | Dry root weight (Zhang et al. 2016) |
| QTSA3_5DAT_01 | 6_42440630 | 6 | 42440630 | G | A | 1.103929 | Dry root weight (Zhang et al. 2016) |
| QTSA3_5DAT_02 | 12_22382546 | 12 | 22382546 | C | T | 1.465376 | Root diameter , root length (Nguyen et al. 2017) |
| PPQ08(TSA3_5DAT, TRV_5DAT) | 20_42464855 | 20 | 42464855 | A | G | 0.083488 | Root bushiness (Abdel-Haleem et al. 2010) |
| PPQ12(TSA3_8DAT, TRV_8DAT) | 12_33823378 | 12 | 33823378 | T | C | -2.05494 | Root diameter , root length (Nguyen et al. 2017) |
| PPQ13(TRL_8DAT, TCA_8DAT) | 4_47289825 | 4 | 47289825 | G | T | 68.19465 | Root bushiness (Abdel-Haleem et al. 2010) |
| PPQ14(TRL_8DAT, TCA_8DAT) | 4_47367011 | 4 | 47367011 | G | A | 76.32488 | Root bushiness (Abdel-Haleem et al. 2010) |
| PPQ15(TRL_8DAT, TCA_8DAT) | 6_48522645 | 6 | 48522645 | C | G | 87.00374 | Primary root length (Brensha et al. 2012) |

Note (abbreviations for traits): Total root length (TRL), Total root volume (TRV), Average root diameter (DIM), Total surface area (TSA), Total surface area of thinner roots (or diameter class 0 to 0.5 mm diameter [(TSA1)] and surface area of thicker roots (or diameter class 1 to 1.5 mm diameter [(TSA3]), Total root width (TRW), Total convex area (TCA), Root depth index (RDI), and Solidity (SOL) and Center of projected mass (COPM) phenotype were calculated from root images at 5 and 8 DAT.

| **Table S10 Genomic position, allelic variation and effect of SNPs significantly associated with the four candidate genes for which we quantified variation in gene expression in lines carrying contrasting alleles for associated SNPs** | | | | | | | |
| --- | --- | --- | --- | --- | --- | --- | --- |
|  |  |  |  |  |  |  |  |
| **QTL** | **SNP** | **Chr** | **Pos** | **Ref** | **Alt** | **Effect** | **% Improvement** |
| QCOPM_8DAT_01 | 4_48983360 | 4 | 48983360 | C | T | 0.680145 | 12.14544 |
| QTRT_5DAT_27 | 14_30630782 | 14 | 30630782 | C | G | -9.11867 | -12.2596 |
| PPQ04(TRL_8DAT, TSA1_8DAT) | 2_8593061 | 2 | 8593061 | G | A | 34.25406 | 15.59413 |
| PPQ08(TSA3_5DAT, TRV_5DAT) | 20_42464855 | 20 | 42464855 | A | G | 0.083488 | 15.40363 |

Note: Trait abbreviations are COPM (center of projected mass), TRT (total number of root tips), TRL (total root length), Total surface area of thinner roots (or diameter class 0 to 0.5 mm diameter [(TSA1)] and surface area of thicker roots (or diameter class 1 to 1.5 mm diameter [(TSA3])), TRV (total root volume)
